# CD8^+^ T cells specific for conserved coronavirus epitopes correlate with milder disease in patients with COVID-19

**DOI:** 10.1126/sciimmunol.abg5669

**Published:** 2021-07-01

**Authors:** Vamsee Mallajosyula, Conner Ganjavi, Saborni Chakraborty, Alana M. McSween, Ana Jimena Pavlovitch-Bedzyk, Julie Wilhelmy, Allison Nau, Monali Manohar, Kari C. Nadeau, Mark M. Davis

**Affiliations:** 1 Institute for Immunity, Transplantation, and Infection, Stanford University School of Medicine, Stanford, CA 94305, USA.; 2 Department of Biology, Stanford University School of Humanities and Sciences, Stanford, CA 94305, USA.; 3 Division of Infectious Diseases, Department of Medicine, Stanford University, Stanford, CA 94305, USA.; 4 Computational and Systems Immunology Program, Stanford University School of Medicine, Stanford, CA 94305, USA.; 5 Sean N. Parker Center for Allergy and Asthma Research, Stanford University and Division of Pulmonary, Allergy, Critical Care Medicine, Stanford University School of Medicine, Stanford, CA 94305, USA.; 6 Department of Microbiology and Immunology, Stanford University School of Medicine, Stanford, CA 94305, USA.; 7 Howard Hughes Medical Institute, Stanford University School of Medicine, Stanford, CA 94305, USA.

## Abstract

A central feature of the SARS-CoV-2 pandemic is that some individuals become severely ill or die, whereas others have only a mild disease course or are asymptomatic. Here, we report the development of an improved multimeric αβ T cell staining reagent platform, with each maxi-ferritin “spheromer” displaying 12 peptide-MHC complexes. Spheromers stain specific T cells more efficiently than peptide-MHC tetramers and capture a broader portion of the sequence repertoire for a given peptide-MHC. Analyzing the response in unexposed individuals, we find that T cells recognizing peptides conserved among coronaviruses are more abundant and tend to have a “memory” phenotype compared with those unique to SARS-CoV-2. Notably, CD8^+^ T cells with these conserved specificities are much more abundant in patients with mild COVID-19 versus those with a more severe illness, suggesting a protective role.

## Teaser

T cells specific for conserved coronavirus antigens are preferentially expanded in patients with mild COVID-19.

## INTRODUCTION

Severe acute respiratory syndrome coronavirus 2 (SARS-CoV-2), the virus causing coronavirus disease 2019 (COVID-19), has infected ~120 million individuals worldwide, displaying a spectrum of disease severities that ranges from asymptomatic to life-threatening pneumonia and multiorgan failure (*1*). Addressing this global pandemic, many pharmaceutical companies and research laboratories have raced to develop effective coronavirus vaccines, of which more than a hundred are in development (*2*). The primary goal of most vaccine development efforts is the generation of neutralizing antibodies targeting the SARS-CoV-2 spike (S) protein. However, the variable magnitude and durability of these antibody responses in patients with COVID-19 highlights the importance of studying T cell–mediated immunity to better understand disease pathogenesis and to develop benchmarks for an effective T cell response (*3*–*6*). Many studies have shown that T cells are involved in a SARS-CoV-2 infection (*7*–*13*), but what types of responses are efficacious and which are not is unclear.

The majority of T cells in most mammals, including human beings, express the αβ T cell receptor (TCR) and recognize a particular peptide bound to a major histocompatibility complex molecule (pMHC) expressed on target cells (*14*). The weak equilibrium dissociation constant (*K*_d_ ~ 1 to 200 μM) between the TCR and monomeric pMHC results in a transient complex that impedes easy detection (*15*). The development of the pMHC tetramer (“tetramer”) technology, wherein the conjugation of four pMHC molecules to streptavidin (SAv) results in the increased avidity of TCR binding, laid the foundation to circumvent this problem (*16*). Since then, several studies have increased the valency of pMHC multimers to improve these reagents’ ability to detect T cells with marginal affinity (*17*–*19*), such as pMHC dextramers that use dextran polymers to increase the number of pMHC. However, the detection of low-affinity TCRs still remains challenging, partly because of an increased background from nonspecific staining using higher valency platforms, thus negatively affecting the signal-to-noise ratio (*19*–*21*).

To improve upon these limitations, we engineered a biotinylation site on maxi-ferritin to create a 24-subunit, self-assembling protein scaffold for the multivalent display of pMHC. This spheromer platform offers several advantages: ease of production, defined site-specific conjugation of pMHC molecules that significantly reduces interbatch variation, and compatibility with currently available pMHC molecules and SAv reagents allowing for facile translation. We show that the spheromer binds both MHC-I– and MHC-II–restricted T cells with excellent specificity for pMHC and at a significantly higher avidity than the tetramer. Furthermore, this reagent provides a better signal-to-noise ratio and detects a much more diverse antigen-specific TCR repertoire in comparison with equivalent tetramers or dextramers. Last, using the spheromer for direct ex vivo study of SARS-CoV-2–specific CD8^+^ T cells, we show that the T cells predicted to cross-react with seasonal human coronaviruses (hCoV) are significantly enriched in COVID-19 patients with mild symptoms in comparison with individuals with severe disease. Because there is evidence that antibodies to SARS-CoV-2 begin to wane not long after infection (*3*, *5*), these robust T cells to conserved epitopes detected in SARS-CoV-2 unexposed individuals and in those with mild disease could be the key determinant in a successful adaptive immune response and could help to explain the disparity in COVID-19 outcomes. Furthermore, following these T cells using spheromer technology could help in tracking SARS-CoV-2 immunity in vaccinated individuals, especially in the context of emerging SARS-CoV-2 mutant strains that, in some cases, escape vaccine-induced antibody responses (*22*).

## RESULTS

**Fig. 1.**
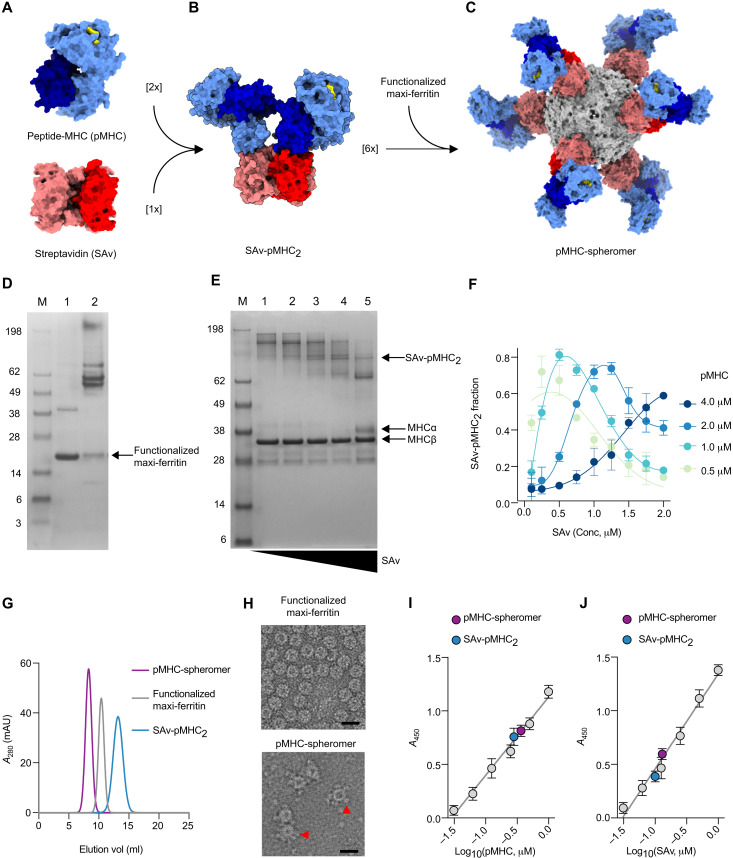
Assembly and characterization of the spheromer. (**A**) Molecular surface representation of pMHC [Protein Data Bank (PDB) ID: 3TO2; α chain in light blue, β2m in dark blue, and peptide in yellow] and SAv (PDB ID: 2RTG; monomer in red and the rest in coral). (**B**) Model of a semisaturated SAv-pMHC_2_ intermediate that has two unoccupied biotin binding sites. A single orientation is shown for simplicity. (**C**) Model of spheromer that is assembled by the conjugation of six semisaturated SAv-pMHC_2_ molecules onto a functionalized maxi-ferritin scaffold (PDB ID:2JD6, gray). UCSF Chimera was used for molecular graphics. (**D**) SAv gel-shift assay to evaluate the functionalization of maxi-ferritin. Lane 1: Biotinylated maxi-ferritin scaffold. The protein dissociates into monomers (23.4 kDa) after boiling and migrates at the corresponding size on a denaturing gel. Lane 2: The flexible tether engineered at the N terminus of each monomer has one biotin binding site. Upon incubation with SAv, migration of the biotinylated maxi-ferritin monomers is retarded because of the formation of a complex. (**E**) Formation of semisaturated SAv-pMHC_2_ monitored by SAv gel-shift assay. The MHC α chain is biotinylated and shifts upon binding SAv. The pMHC was incubated with limiting concentrations of SAv resulting in the formation of oligomers with incremental increase in valency. SDS-PAGE is shown for the titration of an MHC-II molecule with SAv. (**F**) Quantification of SAv-pMHC_2_ formation as a function of pMHC and SAv reactant concentrations. The mean ± SD of the measurements from three experiments is shown. (**G**) Size exclusion chromatogram of the spheromer and its components. mAU, milli-absorbance unit. (**H**) Representative electron micrographs of negatively stained maxi-ferritin and spheromer. SAv-pMHC_2_ conjugated to the surface of the functionalized scaffold is indicated by red arrows. Scale bars, 20 nm. Validation of SAv-pMHC_2_ conjugation to the spheromer using (**I**) anti-MHC and (**J**) anti-SAv antibodies by ELISA (mean ± SD). The experiment was performed with each sample in triplicate and repeated at least twice.

In the search for a protein scaffold that could increase the valency of displayed pMHC and that would hopefully capture more αβ T cells of a given specificity, we focused on self-assembling homo-oligomers (fig. S1A) (*23*, *24*). On the basis of the yield and homogeneity of the recombinantly expressed proteins (fig. S1, B and C), we chose maxi-ferritin for further optimization. Ferritins are naturally occurring cage proteins that participate in biomineral synthesis and are found across almost all living organisms (*23*). Studies have shown that thermophilic proteins denature at a much higher temperature than their mesophilic homologs (*25*). Therefore, we used ferritin derived from the hyperthermophilic archaeal anaerobe *Pyrococcus furiosus* to develop a stable scaffold. Maxi-ferritin forms a 24-subunit nanoparticle with an external diameter of ~120 Å. To develop a platform that is widely accessible, we functionalized the maxi-ferritin scaffold to be compatible with components of the existing tetramer technology that uses biotinylated pMHC monomers and SAv conjugates. We inserted a biotinylation signal sequence (*26*) at the N terminus of each maxi-ferritin subunit (~23-kDa monomer) and used SAv as a “molecular glue” to bring together pMHC monomers and the scaffold (Fig. 1, A to C). We optimized the tethers for SAv on the maxi-ferritin scaffold by testing a set of linkers that spanned a diverse range of lengths and molecular rigidities (fig. S2, A and B) (*27*). As shown, the optimized scaffold with radially projecting tethers could be purified easily and functionalized with biotin (Fig. 1D). We then bound the biotinylated scaffold to SAv conjugated to two peptide-MHC molecules (SAv-pMHC_2_: semisaturated SAv) (Fig. 1, E and F). The SAv-pMHC_2_ precursor formation is not affected substantially by different fluorophores conjugated to SAv, although phycoerythrin (PE) and PE/cyanine7, for instance, are much larger molecules than Alexa 488, eFluor 450, and Alexa 647 (fig. S3A). The semisaturated SAv has two biotin-binding sites available to bind the scaffold. Upon saturation, we observed the display of 12 pMHC molecules as determined by size exclusion chromatography (SEC) and blue native polyacrylamide gel electrophoresis (BN-PAGE) (fig. S4, A and B). The current iteration does not allow the conjugation of more pMHC molecules, presumably because of steric hindrance. It is also possible that two adjacent biotinylated linkers on the scaffold are being occupied by a single SAv-pMHC_2_ molecule. We further purified the homogeneous spheromer by SEC to exclude the contribution from any unreacted SAv-pMHC_2_ (Fig. 1G). We also validated the conjugation of SAv-pMHC_2_ onto the functionalized maxi-ferritin scaffold using negative-stain electron microscopy (EM) (Fig. 1H) and enzyme-linked immunosorbent assay (ELISA) (Fig. 1, I and J). Another objective during the extensive linker (L1-L19) design phase was to optimize the radial projection of the biotin tethers from the maxi-ferritin scaffold to identify a construct [L6: (SG_2_P)_2_SG_2_] that is least affected by different fluorophores. As shown, all the spheromers assembled using the optimized maxi-ferritin scaffold (displaying the L6-linker) and five different fluorophore-conjugated SAv formed a homogeneous complex in solution (fig. S3, B to E).

**Fig. 2.**
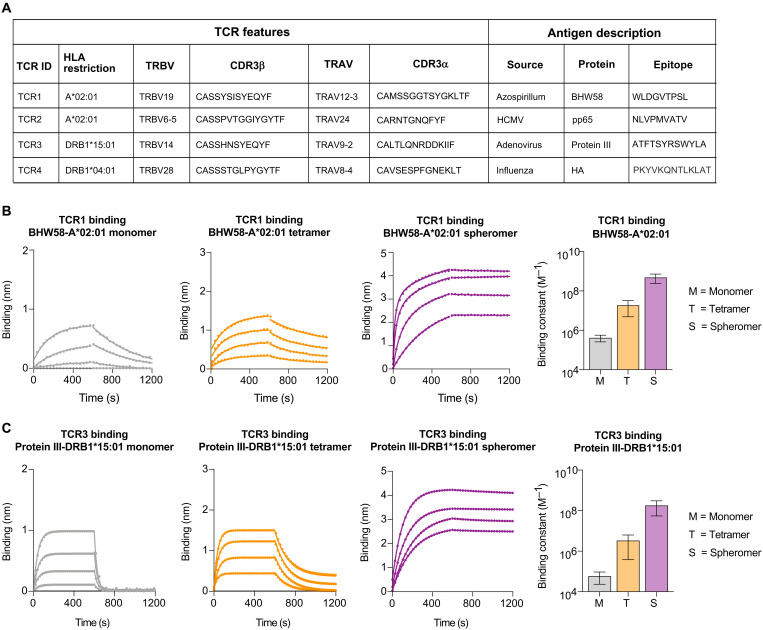
Spheromer binds both MHC-I– and MHC-II–restricted TCRs with high avidity. (**A**) List of evaluated pMHC-TCR pairs. The binding of (**B**) TCR1 and (**C**) TCR3 to different formulations of BHW58-A*02:01 and protein III-DRB1*15:01, respectively, was determined by BLI. An overlay of binding traces over a concentration series of the indicated pMHC formulation from one representative experiment is shown. Each binding experiment was repeated at least three times. The mean ± SD of the binding constant has been graphed.

**Fig. 3.**
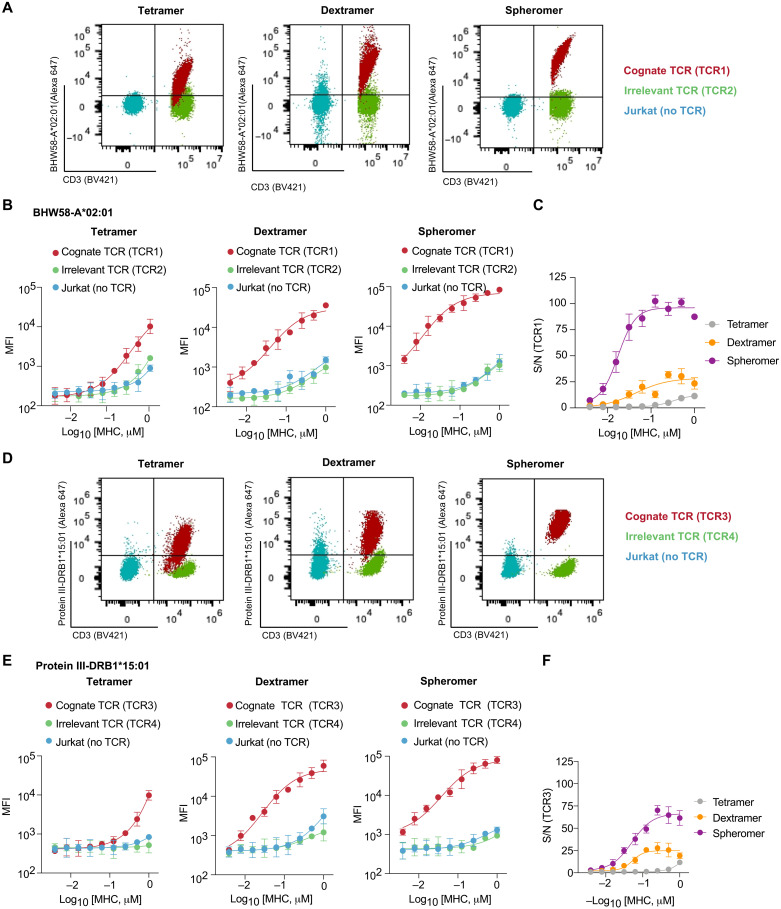
Spheromer binds T cell lines expressing MHC-I– or MHC-II–restricted TCRs with high specificity. (**A**) Representative flow cytometry plots showing the binding of the indicated BHW58-A*02:01 formulations with equivalent pMHC concentration to a T cell line expressing TCR1. The nonspecific binding of the different formulations was measured using untransduced Jurkat cells and a cell line expressing an irrelevant TCR. CD3 was measured as proxy for TCR expression. (**B**) Quantification of BHW58-A*02:01 binding measured by flow cytometry (mean ± SD). The experiment was performed with each sample processed in duplicates and repeated at least twice. (**C**) Signal-to-noise ratio (S/N) of TCR1 binding to distinct BHW58-A*02:01 multivalent formulations. Mean ± SD of the measurements from two independent experiments has been plotted. (**D**) Representative flow cytometry plots showing the binding of the indicated protein III-DRB1*15:01 formulations with equivalent pMHC concentration to a T cell line expressing TCR3. The nonspecific binding to Jurkat cells and an irrelevant TCR was also measured. CD3 was measured as proxy for TCR expression. (**E**) Quantification of protein III-DRB1*15:01 binding (mean ± SD) measured by flow cytometry. (**F**) The signal-to-noise ratio (S/N) of TCR3 binding to distinct protein III-DRB1*15:01 multivalent formulations (mean ± SD).

We characterized the general applicability of the spheromer using a set of TCR-pMHC pairs with distinct TRBV (T cell receptor β variable) usage, antigen sources, and examples representing both MHC-I and MHC-II molecules (Fig. 2A). The binding of TCR with different formulations of their cognate pMHC (monomer, tetramer, and spheromer) was determined using biolayer interferometry (BLI) (Fig. 2, B and C, and fig. S5, A and B). Encouragingly, the spheromer bound all the evaluated TCRs significantly better than the other formulations (monomer and tetramer). On average, for MHC-I restriction, the spheromer bound TCRs with >250-fold (monomer) and >50-fold (tetramer) greater net affinity. For MHC-II–restricted TCRs, the spheromer bound with >200-fold (monomer) and >20-fold (tetramer) greater net affinity across the tested pairs. We also generated stable T cell lines to compare the binding of different pMHC formulations (tetramer, dextramer, and spheromer) using flow cytometry (Fig. 3, A to F, and fig. S6, A to F). As shown for all the evaluated pMHC-TCR pairs, consistent with the increased avidity, the signal from spheromer staining was significantly better (~10-fold) than the tetramer. We included negative controls (TCR^−/−^ Jurkat cells and a cell line expressing irrelevant TCR) to determine background staining because higher valency can result in noise amplification due to nonspecific interactions (*19*, *20*). We observed that although there was an increase in staining intensity with dextramer staining (about sixfold) in comparison with the tetramer, the background staining was also higher. In contrast, the background staining with the spheromer did not increase substantially, resulting in a better signal-to-noise ratio compared with other pMHC formulations (Fig. 3, C and F, and fig. S6, C and F). This difference is likely because the spheromer is a discrete, homogenous structure versus a mix of dextran polymers in the dextramer reagents. The spheromer stains better than the tetramer irrespective of the conjugated fluorophore (fig. S7, A and B). A fluorophore-conjugated maxi-ferritin scaffold can also be alternatively used for assembling the spheromer with unlabeled SAv-pMHC_2_ (fig. S7C).

**Fig. 4.**
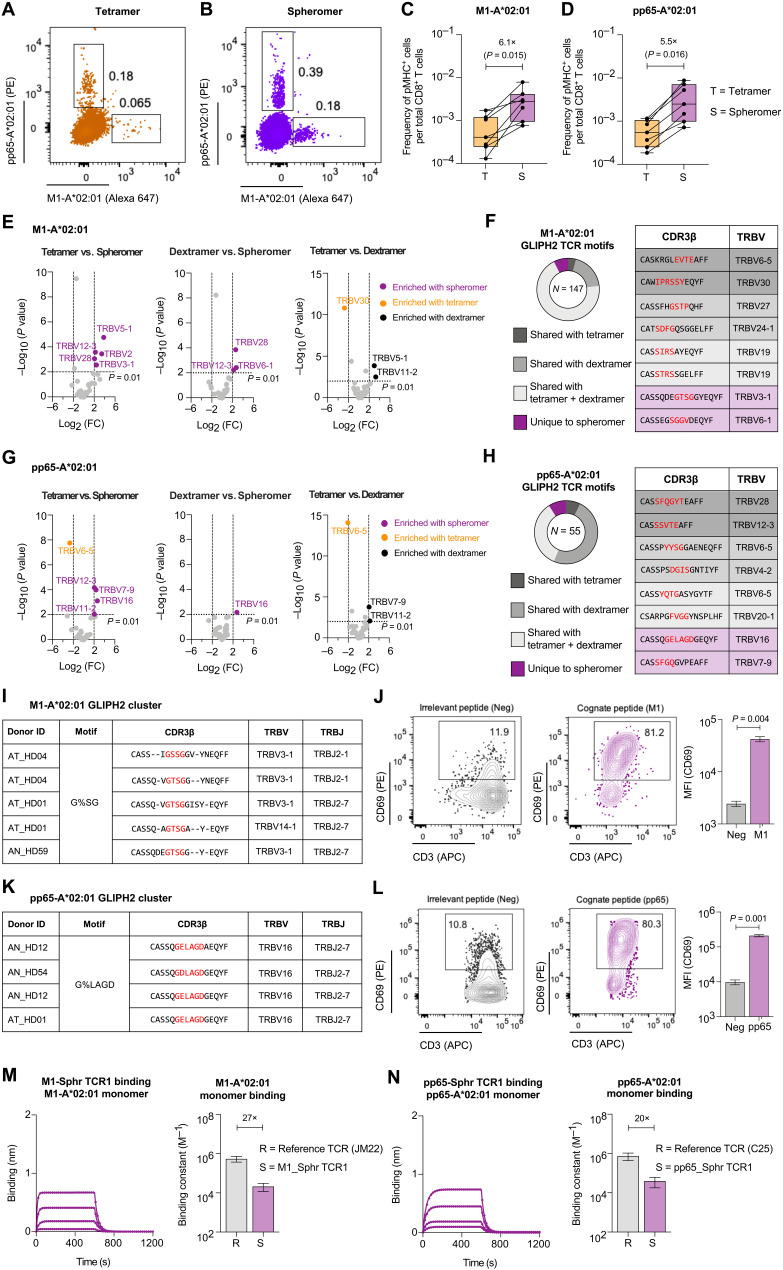
Spheromer detects a higher frequency of antigen-specific T cells with a more diverse TCR repertoire. Representative flow cytometry plots of CD8^+^ T cells isolated from HLA-A*02:01 individuals stained with influenza-M1 and HCMV-pp65 (**A**) tetramers or (**B**) spheromers. Enumeration of epitope-specific (**C**) M1 and (**D**) pp65 CD8^+^ T cells detected in healthy individuals using either tetramer or spheromer. Data from each donor (*n* = 7) are represented by a point. A two-tailed, matched-pairs Wilcoxon signed-rank test was performed to determine the significance levels. (**E**) Volcano plots showing the variance in TRBV usage of M1-A*02:01–specific CD8^+^ T cells detected using the spheromer and other pMHC multimers. The TRBV genes enriched significantly (*P* ≤ 0.01, Fisher’s exact test) are listed; the spheromers are highlighted in purple. (**F**) The distribution of spheromer-derived influenza-M1–specific TCR motifs identified by GLIPH2 and representative examples from each category. (**G**) Volcano plots representing the variance in TRBV usage of pp65-A*02:01–specific CD8^+^ T cells detected with distinct pMHC multimers. The TRBV genes enriched significantly (*P* ≤ 0.01, Fisher’s exact test) are listed; the spheromers are highlighted in purple. (**H**) The distribution of spheromer-derived, HCMV-pp65–specific TCR motifs identified by GLIPH2 and representative examples from each category. (**I**) Representative GLIPH2 cluster with specificity for influenza-M1 composed of TCR sequences identified exclusively using the spheromer. (**J**) Representative flow cytometry plots showing the activation of a T cell line (expressing a TCR with “G%SG” motif) stimulated with an irrelevant or cognate (influenza-M1) peptide. The activation was measured by CD69 expression. The significance level was determined by a two-tailed, paired *t* test. (**K**) Representative GLIPH2 cluster with specificity for HCMV-pp65 that is composed of spheromer-derived TCR sequences exclusively. (**L**) Representative flow cytometry plots showing the activation of a T cell line (expressing a TCR with “G%LAGD” motif) stimulated with an irrelevant or cognate (HCMV-pp65) peptide. The activation was measured by CD69 expression. A two-tailed, paired *t* test was performed to determine significance. The binding of TCR corresponding to clones from GLIPH2 clusters composed exclusively of spheromer-derived sequences to their cognate pMHC monomers (**M**) M1-A*02:01 and (**N**) pp65-A*02:01 determined by BLI. Each binding experiment was repeated at least thrice. The mean ± SD of the binding constant has been graphed and compared with a reference influenza-M1 (JM22)– and HCMV-pp65 (C25)–specific TCR.

Next, we evaluated viral-specific CD8^+^ T cells in healthy individuals to address the following questions: (i) Does the spheromer detect a higher frequency of antigen-specific T cells than tetramer ex vivo? (ii) How do the TCR repertoires detected by the spheromer and tetramer compare? We used immunodominant HLA-A*02:01–restricted epitopes [influenza-M1 and human cytomegalovirus (HCMV)–pp65] for characterizing the spheromer because there are considerable data available for benchmarking (*28*). CD8^+^ T cells isolated from each donor (*n* = 7) were divided evenly for tetramer or spheromer staining (Fig. 4, A and B, and fig. S8, A to C). The frequencies of antigen-specific T cells detected using tetramer are consistent with previous studies (*29*–*32*). As shown, a significantly higher frequency of antigen-specific CD8^+^ T cells could be detected for both M1 (*P* = 0.015) and pp65 (*P* = 0.016) viral specificities (Fig. 4, C and D, and fig. S8D). As expected, the frequency of antigen-specific CD8^+^ T cells in HCMV-negative donors was significantly lower than those in HCMV-positive donors (fig. S8E). We also validated spheromer staining using biotinylated A*02:01 pMHC monomers procured from the National Institutes of Health tetramer core facility, which is a major source of tetramer reagents to the research community worldwide (fig. S9, A to D). Next, we single cell–sorted spheromer^+^ CD8^+^ T cells and performed paired αβ-TCR sequencing to study the repertoire (*33*). The spheromer-derived TCR sequences were analyzed against TCR entries in VDJdb, a curated database of TCRs with known antigen specificities (*28*). We compared the TRBV usage of TCR sequences obtained using distinct pMHC formulations (Fig. 4, E and G). Overall, we observed that the spheromer detected a much more diverse repertoire in comparison with either the tetramer or dextramer. As shown, the M1-specific TCR sequences detected with the spheromer had a significantly (*P* < 0.01, Fisher’s test) higher usage of five and three TRBV genes in comparison with the tetramer- and dextramer-derived sequences, respectively, with two overlapping genes (TRBV12-3 and TRBV28) across them (Fig. 4E). Similarly, spheromer^+^ pp65 TCR sequences showed an enrichment of four TRBV genes in comparison with the tetramer and one TRBV gene with the dextramer (Fig. 4G). TRBV6-5 is significantly enriched in tetramer^+^ pp65^+^ TCR sequences when compared with both the dextramer- and spheromer-derived sequences. We further analyzed the specificity of spheromer-derived TCR sequences using GLIPH2 (grouping of lymphocyte interaction by paratope hotspots), an algorithm that clusters TCRs based on shared antigen specificity (Fig. 4, F and H) (*34*). Globally, we observed a significant overlap (~91%) between the TCR “motifs” identified using spheromer- and antigen-specific TCR entries in VDJdb. The recovery of previously characterized antigen-specific TCR motifs using the spheromer provides further confirmation that our designed platform is detecting relevant T cells. The spheromer could detect previously described public TCRs for both M1 (CDR3b: CASSIRSSYEQYF, CASSIRSAYEQYF) and pp65 (CDR3b: CASSYQTGASYGYTF) viral specificities shown to have a significant association with HLA-A*02:01 (*35*, *36*). The spheromer identified a set of TCR motifs that did not cluster with sequences previously reported in VDJdb (8% for M1 and 9% for pp65). To test whether these TCRs could confer reactivity to the pMHCs they were selected with, we generated T cell lines with TCRs from these previously unidentified GLIPH2 clusters (Fig. 4, I and K, and fig. S10, A and C). As shown using CD69 expression, these T cell lines could be activated specifically using the cognate peptide (Fig. 4, J and L, and fig. S10, B and D). We also measured the TCR binding of these clones to their cognate pMHC monomers by BLI. As shown, TCRs detected exclusively using the spheromer on average bound the pMHC monomer with ~30-fold lower affinity in comparison with previously reported reference TCRs (Fig. 4, M and N, and fig. S10, E and F) (*37*, *38*). These results demonstrate that spheromer reagents are not just more efficient at staining the relevant T cells but can also identify low-affinity antigen-specific T cells that may not be detected with other multimer reagents.

**Fig. 5.**
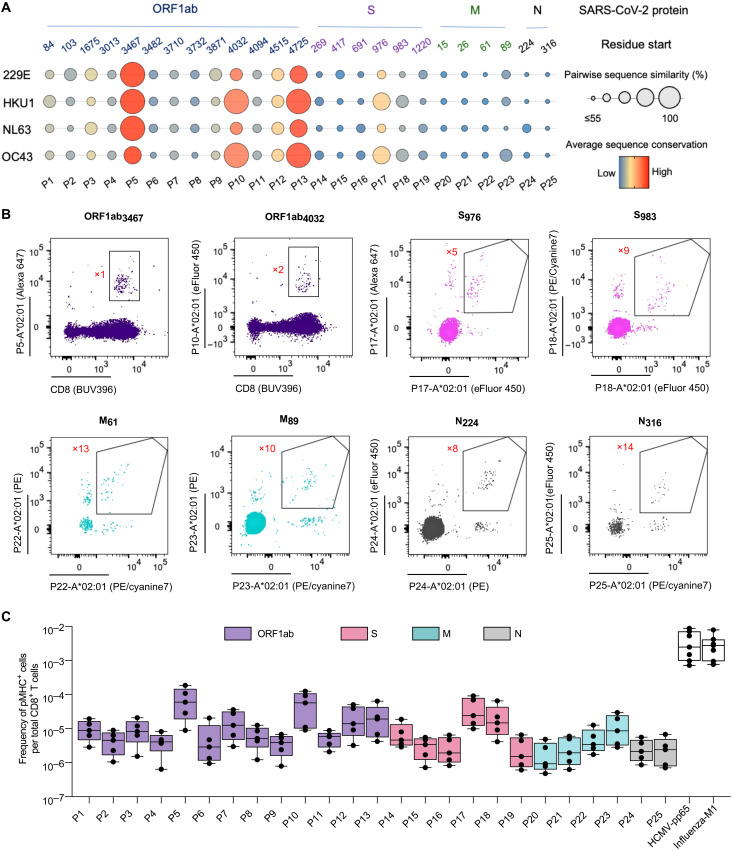
The frequency of CD8^+^ T cells against SARS-CoV-2 epitopes conserved across seasonal hCoVs is elevated in unexposed individuals. (**A**) Sequence conservation of SARS-CoV-2 epitopes across seasonal hCoVs. The epitopes were selected on the basis of their biochemical properties and binding to HLA-A*02:01. These peptides span multiple SARS-CoV-2 coding regions (ORF1ab, S, M, and N) and display varying degrees of sequence similarity. The pairwise conservation score between SARS-CoV-2 and any given hCoV is indicated by the size of the bubble. The color represents the average conservation score across all hCoVs. (**B**) Representative flow cytometry plots of combinatorial, antigen-specific staining of PBMCs from an unexposed individual using HLA-A*02:01 spheromer pools after magnetic enrichment. The fluorophore barcode as shown in the supplementary information used to determine antigen specificity is labeled in red next to the gated population. (**C**) Enumeration of SARS-CoV-2 epitope–specific CD8^+^ T cells in unexposed, prepandemic PBMC samples collected between April 2018 and February 2019. Data from each donor (*n* = 5) are represented by a dot. The frequency of SARS-CoV-2–specific T cells in unexposed individuals is lower than HCMV-pp65– and influenza-M1–specific T cells.

**Fig. 6.**
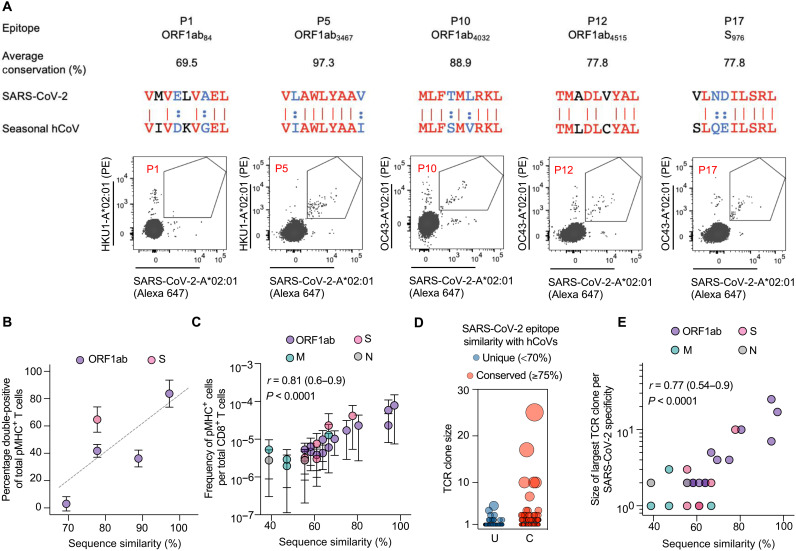
Cross-reactivity between SARS-CoV-2 and seasonal hCoV CD8^+^ T cell epitopes. (**A**) Representative flow cytometry plots showing the costaining of CD8^+^ T cells from unexposed individuals using spheromers displaying the indicated SARS-CoV-2 and seasonal hCoV A*02:01 bound peptides after magnetic enrichment. The average conservation score across all hCoVs for each epitope is listed. For the pairwise sequence comparison: identical residues (red), synonymous residues defined in our substitution matrix (blue), and the rest (black). (**B**) Correlation between the fraction of costained CD8^+^ T cells and the average sequence similarity of SARS-CoV-2 epitopes with hCoVs in healthy, unexposed individuals (*n* = 3). (**C**) A positive correlation was observed between the average sequence similarity of SARS-CoV-2 epitopes with hCoVs and the baseline frequency of SARS-CoV-2 epitope–specific CD8^+^ T cells in healthy, unexposed individuals. (**D**) Evaluation of clonal expansion in unexposed individuals using single-cell TCR sequencing of SARS-CoV-2–specific CD8^+^ T cells identified using spheromer. A summary plot of TCR clonality across all SARS-CoV-2 epitopes tested in this study. The data were divided into two groups (unique or conserved) based on a threshold of ≥75% (allowing for two mismatches in a given 9-mer). Each individual dot represents a distinct TCR clone. (**E**) Correlation between the average sequence similarity of SARS-CoV-2 epitopes with hCoVs and size of the largest TCR clone of the corresponding specificity.

**Fig. 7.**
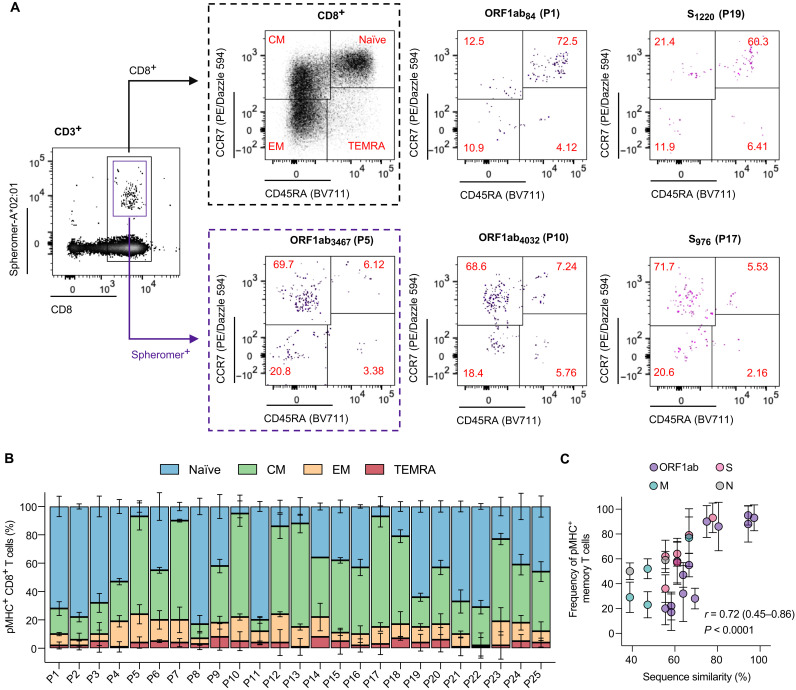
CD8^+^ T cells in unexposed individuals against conserved SARS-CoV-2 epitopes exhibit a predominant memory phenotype. (**A**) Representative flow cytometry plots showing the distribution of SARS-CoV-2–specific CD8^+^ T cells across the naïve and memory subsets defined on the basis of the expression of CD45RA and CCR7 markers: naïve (CD45RA^+^CCR7^+^), central memory (CM, CD45RA^−^CCR7^+^), effector memory (EM, CD45RA^−^CCR7^−^), and effector memory expressing CD45RA (TEMRA, CD45RA^+^CCR7^−^) in healthy, unexposed individuals. The antigen-specific CD8^+^ T cells were enriched using magnetic beads. (**B**) Quantification of SARS-CoV-2–specific CD8^+^ T cells across the naïve and memory subsets in healthy, unexposed individuals. (**C**) Correlation between the average sequence similarity of SARS-CoV-2 epitopes across hCoVs and the frequency of memory (nonnaïve) CD8^+^ T cells.

**Fig. 8.**
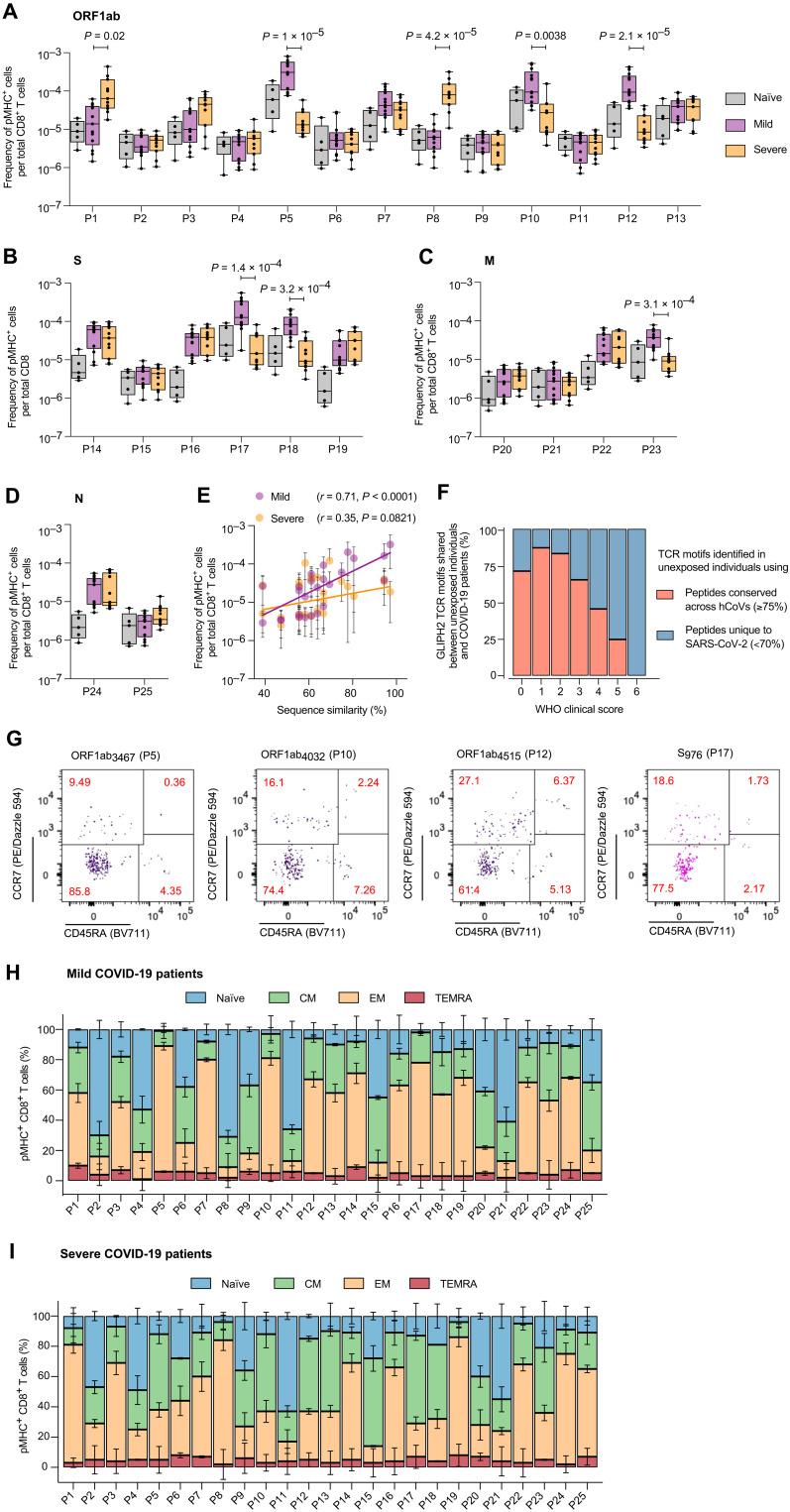
COVID-19 patients with divergent clinical outcomes exhibit distinct SARS-CoV-2 epitope–specific CD8^+^ T cell responses. The frequency of SARS-CoV-2 epitope–specific CD8^+^ T cells across unexposed individuals and COVID-19 patients with mild (*n* = 13) and severe (*n* = 11) infections: (**A**) ORF1ab, (**B**) S, (**C**) M, and (**D**) N. The adjusted *P* value as determined by Dunn’s test corrected for multiple comparisons is reported for specificities with a significant difference between patients with mild and severe COVID-19. (**E**) Correlation between the average sequence similarity of SARS-CoV-2 epitopes across hCoVs and the frequency of antigen-specific CD8^+^ T cells in patients with COVID-19. (**F**) The distribution of SARS-CoV-2–specific TCR motifs shared between unexposed individuals and patients with COVID-19. TCR motifs were identified using GLIPH2. A lower WHO score indicates milder symptoms. TCR motifs shared between unexposed individuals and patients with mild COVID-19 were identified by conserved SARS-CoV-2 epitopes. In contrast, TCR motifs characterizing patients with severe COVID-19 were detected in unexposed individuals using peptides that were primarily unique to SARS-CoV-2 (adjusted *P* = 0.00019, Fisher’s test). (**G**) Representative flow cytometry plots showing the distribution of SARS-CoV-2–specific CD8^+^ T cells across the naïve and memory subsets in patients with COVID-19. The antigen-specific CD8^+^ T cells were enriched using magnetic beads. Quantification of SARS-CoV-2–specific CD8^+^ T cells across the naïve and memory subsets in patients with (**H**) mild and (**I**) severe COVID-19.

To address the immune response to SARS-CoV-2, we made spheromer reagents to evaluate CD8^+^ T cell responses in unexposed individuals and in patients with COVID-19 (tables S1 and S2). We have previously shown that T cells to viral epitopes can be detected in the peripheral blood of naïve individuals (*39*, *40*). Significantly, a large fraction (~50%) of these T cells in adults (28 to 80 years) exhibited a memory phenotype, possibly because of higher TCR cross-reactivity or environmental exposures (*39*). The rapid recruitment of these T cells in an immune response could offer a survival advantage because clonal expansion and the induction of memory lymphocytes are a key goal of vaccination efforts and strongly correlate with protection against particular infectious diseases. Previous studies have also shown that T cell precursor frequencies correlate with the magnitude of antiviral responses (*41*–*43*). Therefore, we determined the frequency of CD8^+^ T cells against a panel of SARS-CoV-2 epitopes (Fig. 5A) in naïve, unexposed individuals using the spheromer. The peptides were selected from multiple SARS-CoV-2 open reading frames (ORFs) spanning ORF1ab, S, M, and N proteins (table S3). The peptides (9-mers) evaluated in this study were chosen on the basis of the predicted binding affinity to HLA-A*02:01 determined using the immune epitope database and analysis resource (IEDB) recommendations (http://tools.iedb.org/mhci/) (*44*) and cross-validated using the SYFPEITHI algorithms (*45*). Furthermore, the biochemical properties of amino acids at positions P2, P5, and P9 were given higher weights (*40*, *46*). We used an MHC stabilization assay to further validate the binding of peptides to A*02:01 MHC-I molecules expressed on the antigen processing (TAP)–deficient T2 cell line (fig. S11). We also designed our peptide panel to represent a diverse range of sequence similarities with peptides from common cold–causing hCoV (hCoV-OC43, HKU1, 229E, and NL63) to evaluate cross-reactive responses. The amino acid substitution matrix to determine sequence conservation was chosen on the basis of previous studies (*47*, *48*) to prioritize SARS-CoV-2 T cell epitopes, but it must be noted that exceptions defined by an idiosyncratic TCR cross-reactivity profile will exist. We used a combinatorial staining approach as described previously to simultaneously probe for multiple specificities in a single sample followed by magnetic enrichment of antigen-specific CD8^+^ T cells (fig. S12) (*49*). In unexposed individuals, we observed that a few SARS-CoV-2 epitopes (P5, P10, P12, P13, P17, and P18) had an elevated CD8^+^ T cell frequency (2.07 × 10^−4^ ± 1.16 × 10^−4^) when compared with other peptides (2.96 × 10^−5^ ± 2.01 × 10^−5^) in the panel (Fig. 5, B and C), albeit at lower levels than the frequency of T cells against immunodominant epitopes of other viruses (HCMV and influenza) (Fig. 5C). We determined the limit of detection after magnetic enrichment to be ~2 × 10^−7^. We experimentally validated the cross-reactivity between a subset of SARS-CoV-2 and seasonal hCoV epitopes (Fig. 6, A and B). Generally, the epitopes to which we observed elevated T cell frequencies in unexposed individuals were characterized by high sequence similarity with hCoVs (Fig. 6C). TCR sequencing of CD8^+^ T cells from unexposed individuals identified using spheromers presenting SARS-CoV-2 epitopes showed that T cells against peptides conserved across coronaviruses are relatively expanded in comparison with T cells against peptides unique to SARS-CoV-2 (Fig. 6, D and E). Phenotypic characterization of these antigen-specific T cells using CCR7 and CD45RA markers showed a distinct distribution between the naïve/memory compartments for the tested peptides (Fig. 7, A to C). T cells detected with peptides having low hCoV sequence similarity demonstrated a predominantly naïve phenotype. In contrast, peptides against which relatively elevated T cell frequencies were observed in unexposed individuals showed a memory phenotype (~80%) and correlated with high hCoV sequence similarity. This suggests that exposure to seasonal hCoVs among other cross-reactive environmental exposures could contribute to the observed expansion of these T cells. Next, we determined the CD8^+^ T cell frequencies against these SARS-CoV-2 epitopes in patients with COVID-19 presenting mild or severe symptoms. We observed that in addition to the spike protein (S, *n* = 4/6), CD8^+^ T cells against epitopes from other SARS-CoV-2 proteins (ORF1ab, *n* = 3/13; M, *n* = 2/4; and N, *n* = 1/2) were also present at a significantly higher frequency in patients with COVID-19 (mild/severe) when compared with unexposed individuals (Fig. 8, A to D). We observed that CD8^+^ T cell frequencies to specific epitopes were significantly different comparing patients with mild and severe COVID-19. In general, the peptides that showed a higher response in severe patients had a lower similarity to other hCoVs. In contrast, patients exhibiting mild symptoms showed an elevated response to peptides with relatively higher sequence similarity to other hCoVs (Fig. 8E). Using GLIPH2, we could identify TCR motifs shared between unexposed individuals and patients with COVID-19 (Fig. 8F). TCR motifs against conserved epitopes are enriched in COVID-19 patients with mild symptoms. In contrast, TCR motifs characterizing patients with severe COVID-19 were detected using peptides that were primarily unique to SARS-CoV-2 (adjusted *P* = 0.00019, Fisher’s test). A high fraction of these antigen-specific CD8^+^ T cells enriched in patients with mild COVID-19 displayed an effector phenotype indicating recent antigen activation (Fig. 8, G to I). This suggests that T cells found in unexposed individuals that bind SARS-CoV-2 epitopes could be actively recruited during infection. Overall, our data suggest a preferential recruitment of memory CD8^+^ T cells specific for conserved epitopes, which are likely the result of previous hCoV exposures in patients with COVID-19 developing mild symptoms.

## DISCUSSION

Antigen-specific T cell responses are known to be essential for an effective immune response against many infectious diseases, but defining specific benchmarks for what is protective versus what is not has been challenging, especially in human studies (*50*, *51*). This is due to many factors, including the low frequency of disease-relevant T cells, particularly when clinical samples are limiting as they typically are. Consequently, some methods used to investigate T cells necessitate expansion of cells in culture, which may alter the relative abundance and phenotype of some T cell clonotypes. Also, the TCR repertoire cannot be studied with some of these methods because of their incompatibility with sequencing techniques. The development of tetramer technology partially addressed this limitation and enabled the direct measurement and characterization of T cells ex vivo. Subsequent advances, in terms of both reagents and methods, have widened the scope of applications (*17*–*19*, *49*, *52*–*56*). However, the detection of low-affinity T cells is still lacking in many cases (*18*).

Here, we report the development of a multivalent spheromer system built on the scaffold of a self-assembling maxi-ferritin nanoparticle. As shown, the system has been engineered to be compatible with current pMHC (both MHC-I and MHC-II molecules) and SAv reagents that allows ease of use. The optimized spheromer assembly pipeline resulted in a very consistent reagent across multiple batches of synthesis with a relative ease of production, unlike the dodecamer (*19*). The defined geometry of the scaffold facilitated precise site-directed conjugation of pMHC, leading to a relatively homogenous reagent as assessed using a size exclusion column. The spheromer bound cognate TCRs with a significantly higher avidity when compared with the tetramer for both MHC-I (>50-fold) and MHC-II (>20-fold) molecules. Also, the low background contributed to the better signal-to-noise ratio observed in comparison with other pMHC formulations tested. The improved TCR-binding properties of the spheromer may also be, in part, due to better two-dimensional binding kinetics owing to its larger diameter. This may provide a better surrogate than either the tetramer or dextramer for membrane-embedded pMHC molecules that engage TCRs in vivo. This increased avidity and specificity can potentially enable the detection of more disease-relevant, low-affinity T cells. Using the HLA-A*02:01–restricted influenza-M1 and HCMV-pp65 epitopes, we demonstrated that a significantly higher frequency of antigen-specific CD8^+^ T cells with a much more diverse TCR repertoire could be detected with the spheromer. These results demonstrate that our engineered scaffold can be readily adapted with currently available reagents without a time-consuming systemic overhaul.

We further applied the spheromer technology to delineate the CD8^+^ T cell response to SARS-CoV-2 using a panel of peptides derived from multiple proteins (ORF1ab, S, M, and N) that were validated for HLA-A*02:01 binding. Studies have shown that a T cell response can be generated against multiple SARS-CoV-2 proteins (*7*–*13*). We observed a relatively higher frequency of T cells against a few epitopes in the ORF1ab (P5, P10, P12, and P13) and S (P17 and P18) proteins in naïve, unexposed individuals. The high sequence similarity of these epitopes to hCoVs and the predominant memory phenotype of these T cells suggest that exposure to seasonal coronaviruses could contribute to the expansion of potentially cross-reactive T cells. The frequency of T cells against a subset of these cross-reactive peptides (P5, P10, P12, and P17) was significantly higher in COVID-19 patients with mild symptoms. In contrast, T cells to unique ORF1ab-derived peptides (P1 and P8) were higher in severely ill patients with COVID-19. These peptides (P1 and P8) have low sequence similarity to hCoVs. Overall, our data indicate that patients with mild and severe COVID-19 elicit distinct T cell responses to particular SARS-CoV-2 epitopes. Also, the preferential recruitment of memory CD8^+^ T cells to cross-reactive epitopes likely contributes to their mild symptoms. These cross-reactive T cell responses need to be investigated in children as they may contribute to their milder clinical symptoms when compared with adults (*57*) because seasonal hCoV infections are more frequent in children than adults (*58*). This study suggests that, in addition to preexisting cross-reactive memory CD4^+^ T cells reported previously (*10*), dissimilar SARS-CoV-2 epitope–specific CD8^+^ T cell responses could also contribute to divergent COVID-19 clinical outcomes. The observation of CD8^+^ T cell responses to multiple SARS-CoV-2 proteins is consistent with previous studies. Accordingly, the data presented here suggest that the incorporation of additional nonspike epitopes into a vaccine could further bolster antiviral T cell immunity. This can be important given the emergence of several SARS-CoV-2 variants of concern (https://cdc.gov/coronavirus/2019-ncov/cases-updates/variant-surveillance/variant-info.html). Sequence analysis of SARS-CoV-2 epitopes found to be associated with mild symptoms in our study across variants indicates that one of the two spike protein epitopes (P17: VLNDILSRL) has mutated (S→A) in the B.1.1.7 lineage variants circulating in Europe. In contrast, none of the nonspike protein epitopes associated with mild symptoms was mutated across the analyzed variants (*59*, *60*).

Overall, this study demonstrates the potential of the spheromer technology but is limited in terms of the specificities and samples used for comparing the different pMHC-multimer platforms. Extending these results to other class I and class II human leukocyte antigen (HLA) alleles will be important in the future, but the results shown here are consistent across different antigens complexed to HLA-A*02:01, and in our experience, it would be surprising if it was not advantageous to use this platform for other HLA alleles as well.

## MATERIALS AND METHODS

### Study design

The objective of this study was to measure cross-reactive CD8^+^ T cell immunity between seasonal coronaviruses that cause the common cold and SARS-CoV-2. We measured the frequency of antigen-specific T cells in unexposed prepandemic donors and in patients with COVID-19 presenting mild or severe symptoms to evaluate the contribution of preexisting immunity to seasonal coronaviruses in disease resolution. For direct, ex vivo detection of antigen-specific T cells at single epitope resolution, we developed an improved multimeric αβ T cell staining spheromer reagent.

### Design, expression, and characterization of multimeric protein scaffolds

To develop an optimized self-assembling protein scaffold for the multivalent presentation of pMHC molecules, we designed and tested several (*n* > 30) protein constructs. All constructs were codon-optimized for expression in mammalian cells. Gene blocks (Integrated DNA Technologies) corresponding to individual constructs were cloned into a vector with a CMV/R promoter by Gibson assembly (New England Biolabs) and sequence-confirmed (Elim Biopharm).

We first evaluated the heterologous recombinant expression of self-assembling proteins with different oligomeric states (*n* = 12, 24, and 60). The sequences corresponding to mini-ferritin (12-nucleotide oligomer, UniProt accession ID: P0ABT2), maxi-ferritin (24-nucleotide oligomer, UniProt accession ID: Q8U2T8), and lumazine synthase (60-nucleotide oligomer, UniProt accession ID: E6PLJ8) were cloned and expressed in Expi293F cells (Thermo Fisher Scientific) as per the manufacturer’s recommendations. Briefly, 100 ml of Expi293F cells subcultured at a density of 3 × 10^6^ viable cells/ml in Expi293 expression medium (Thermo Fisher Scientific) was transfected with the expression plasmids complexed with ExpiFectamine 293 transfection reagent. The next day (~18 to 22 hours after transfection), the cells were supplemented with a cocktail of enhancers. The cell cultures were further incubated for 4 days. Subsequently, the culture supernatants were harvested by centrifugation (2000*g*, 30 min, 4°C) for protein purification. The supernatants were filtered [0.45-mm polyethersulfone (PES) membrane filters, Thermo Fisher Scientific] and diluted with 20 mM tris-HCl (pH 8). The proteins were bound to a HiTrap Q FF anion exchange column (Cytiva) using an ÄKTA pure 25 L1 system (Cytiva). An NaCl gradient (in 20 mM tris-HCl, pH 8) was used to elute the bound proteins. The yield and purity of the multimeric protein scaffolds were estimated using a NuPAGE bis-tris 4 to 12% gradient gel system (Thermo Fisher Scientific). The homogeneity of the purified proteins was assessed using size exclusion columns [Superdex 200 Increase 10/300 GL, Superose 6 Increase 10/300 GL (Cytiva)] that were calibrated using a wide range of molecular weight standards (Bio-Rad).

On the basis of protein yield and homogeneity, we further optimized the maxi-ferritin scaffold for pMHC display by testing multiple linkers varying in length and rigidity. A list of all the evaluated linkers is given in fig. S2A. Each construct was expressed in mammalian cells and purified as described above. The protein construct with linker (SG_2_P)_2_SG_2_ (L6) was chosen for spheromer assembly based on yield and optimal radial projection from the scaffold. The sequence of the optimized maxi-ferritin scaffold is given in fig. S2B. Site-directed functionalization (biotinylation) of the scaffold was performed using BirA biotin-protein ligase. The purified scaffold was incubated with components of the biotinylation reaction as per the manufacturer’s recommendation (Avidity). The functionalized scaffold was subsequently separated from free biotin using a Superdex 200 Increase 10/300 GL (Cytiva) size exclusion column. Next, the efficiency of protein biotinylation was assessed using a SAv gel-shift assay. Briefly, the protein was boiled at 90°C for 7 min before incubation on ice for 10 min. Subsequently, a twofold molar excess of SAv (Agilent) was added to the protein and incubated further for an additional 10 min on ice. The shift in mobility of the scaffold resulting from SAv binding was evaluated using the NuPAGE bis-tris 4 to 12% gradient gel system (Thermo Fisher Scientific).

### Spheromer assembly and characterization

The spheromer assembly is a two-step process: (i) generation of a semisaturated SAv-pMHC_2_ complex and (ii) conjugation of SAv-pMHC_2_ to the functionalized maxi-ferritin scaffold. We optimized the reaction conditions for getting the maximum yield of SAv-pMHC_2_ by varying the reactant concentrations, incubation time, agitation conditions, and reaction temperature. We evaluated the formation of SAv-pMHC_2_ by SEC (Cytiva) and NuPAGE bis-tris 4 to 12% gradient gel system (Thermo Fisher Scientific). The maximum yield of SAv-pMHC_2_ was obtained by incubating 1 μM pMHC (monomer) with 0.45 μM SAv at 25°C for 30 min without agitation. Subsequently, the spheromer complex was assembled by incubating SAv-pMHC_2_ with the functionalized scaffold for 1 hour at room temperature with mild rotation. The unconjugated and fluorophore-conjugated SAv were sourced from Agilent and Invitrogen, respectively. We determined the stoichiometry of pMHC saturation on the spheromer by incubating the functionalized scaffold with increasing concentrations of SAv-pMHC_2_ and analyzing the resulting product on size exclusion columns calibrated using a broad range of molecular weight standards. The complexes were also assessed by BN-PAGE as per the manufacturer’s recommendations (Thermo Fisher Scientific). We further purified the spheromer assembly using a size exclusion column to mitigate the confounding effects from any unreacted SAv-pMHC_2_.

We also validated the conjugation of pMHC onto the functionalized scaffold by negative-stain EM. Five microliters of the purified samples (0.005 to 0.5 mg/ml) was applied on glow-discharged carbon-coated grids, blotted, and stained with 1% uranyl formate according to standard protocols (*61*). Negatively stained grids were imaged on an FEI Morgagni at 100 kV.

The number of pMHC molecules conjugated to the engineered maxi-ferritin scaffold was also quantified by ELISA using standard curves generated for pMHC and SAv. Briefly, test samples were coated on 96-well Nunc plates (Thermo Fisher Scientific) at 2 mg/ml in 50 μl of phosphate-buffered saline (PBS, pH 7.4) at 37°C for 1 hour. Plates were then washed with PBS containing 0.05% Tween 20 (PBST) and blocked with 3% skim milk in PBST for 1 hour. The plates were washed and incubated at room temperature with 50 μl of horseradish peroxidase (HRP)–conjugated anti-SAv immunoglobulin G (Abcam) in blocking buffer at a predetermined dilution (1:5000) for 1 hour for the detection of SAv. Alternatively, MHC-I and MHC-II molecules were detected using HRP-conjugated anti-human HLA-A2 antibody (LSBio) or HRP-conjugated anti-human HLA-DR antibody (LSBio). Plates were washed with PBST and developed with 75 μl per well of the substrate 3,3′,5,5′-tetramethylbenzidine (TMB) solution (MilliporeSigma). The reaction was stopped with 100 μl per well of ELISA stop solution for TMB (Thermo Fisher Scientific). The optical density at 450 nm was measured using the FlexStation 3 Multi-Mode Microplate Reader (Molecular Devices) and corrected for any nonspecific background signal from ovalbumin-coated wells.

### Cloning, expression, and purification of soluble TCRs

The soluble TCRs were expressed and purified as described previously (*62*). Briefly, for each TCR, the extracellular domains corresponding to the TCRα and TCRβ chains were codon-optimized for expression in insect cells and cloned independently into a baculovirus expression vector optimized for TCR expression by Gibson assembly (New England Biolabs). The sequence-confirmed (Elim Biopharm) plasmids were amplified in *Escherichia coli* (New England Biolabs). Each plasmid was cotransfected with BestBac Linearized Baculovirus DNA (Expression Systems) into Sf9 insect cells (Expression Systems) using Cellfectin II for the production of baculoviruses. The P1 stocks of TCRα and TCRβ baculoviruses of a given TCRαβ pair were titrated to ensure a 1:1 TCRαβ heterodimer formation and then cotransduced into High Five cells (Thermo Fisher Scientific). After 3 days, the supernatant was collected by centrifugation. A precipitation mix [50 mM tris-HCl (pH 8), 1 mM NiCl_2_, and 5 mM CaCl_2_] was added to the supernatant while stirring for 15 min at 25°C. The precipitate was subsequently removed by centrifugation, and the supernatant was incubated with buffer-equilibrated nickel-nitrilotriacetic acid (Ni-NTA) beads (Qiagen) for 4 hours at 25°C under mild mixing conditions. Then, the Ni-NTA beads were collected and washed with 20 mM imidazole in Hepes-buffered saline (HBS; pH 7.2). The bound protein was eluted using 200 mM imidazole in HBS (pH 7.2). The TCRαβ heterodimer was further purified by a size exclusion column [Superdex 200 Increase 10/300 GL (Cytiva)] using an ÄKTA pure 25 L1 system (Cytiva) equilibrated with HBS (pH 7.2). The eluted fractions were analyzed for purity using SDS-PAGE and subsequently pooled.

### MHC-I protein purification and peptide exchange

To generate HLA-A*02:01 (MHC-I) monomers, the corresponding α-chain and β2m protein constructs were overexpressed separately in *E. coli*. The protein was refolded from the inclusion bodies in the presence of an ultraviolet (UV)–cleavable peptide and biotinylated for downstream applications as described previously (*63*). After purification, the protein was concentrated and stored with 20% glycerol at −80°C. For each epitope specificity tested in this study, peptide exchange reactions were set up in a volume of 100 μl containing 0.2 mM peptide and HLA-A*02:01 protein (100 μg/ml) in PBS (pH 7.4). The reaction mixture was exposed to 365 nm UV light irradiation for 20 min using a Stratagene UV Stratalinker 2400 in 96-well U-shaped bottom microplates (Corning). The plate was then transferred to 4°C overnight to complete the exchange. The protein was subsequently buffer-exchanged against PBS (pH 7.4) using Microcon centrifugal filters (10-kDa cutoff, MilliporeSigma) to remove the excess free peptide and subsequently spun at 13,000*g* for 15 min at 4°C to remove aggregates. The protein was filtered and stored at 4°C until further use.

### Purification of MHC-II heterodimers and peptide exchange

The ectodomains of HLA-DRA, HLA-DRB1*04:01, and HLA-DRB1*15:01 were cloned into a CMV/R promoter–based vector by Gibson assembly (New England Biolabs). The gene constructs were codon-optimized for mammalian expression. The sequence-confirmed (Elim Biopharm) plasmids were amplified in *E. coli*. Plasmids encoding the MHCα and MHCβ chains of a given MHCαβ heterodimer were cotransfected into Expi293F cells (Thermo Fisher Scientific) following the manufacturer’s recommendations. The transfected cells were enhanced ~18 to 20 hours after transfection with ExpiFectamine 293 transfection enhancers 1 and 2 (Thermo Fisher Scientific). The supernatant was harvested 5 days after transfection and incubated with buffer-equilibrated Ni-NTA beads (Qiagen) for 5 hours at 4°C. The Ni-NTA beads were then collected and washed [20 mM imidazole in HBS (pH 7.2)], and the bound protein was eluted under gravity flow with 200 mM imidazole in HBS (pH 7.2). The protein was buffer-exchanged to remove the imidazole and biotinylated using the BirA biotin-protein ligase reaction kit (Avidity) as per the manufacturer’s recommendations. The MHC-II heterodimer was subsequently purified via SEC [Superdex 200 Increase 10/300 GL (Cytiva)] using an ÄKTA pure 25 L1 system (Cytiva) equilibrated with HBS (pH 7.2). The eluted fractions were analyzed for purity, pooled, and also assessed for biotinylation efficiency using SDS-PAGE. Thrombin (Novagen) was used to cleave the invariant CLIP peptide from the purified MHC-II molecules to enable exchange with the test peptide. After 2-hour incubation of MHC-II molecules with thrombin at room temperature, the reaction was stopped by the addition of a protease inhibitor cocktail (MilliporeSigma). The cleaved MHC-II protein was incubated at 30°C overnight in an aqueous solution of 1% octyl β-d-glucopyranoside, 0.1 M NaCl, 50 mM citrate (pH 5.2), 1 mM EDTA, and test peptide (0.4 mg/ml) for completion of exchange. The next day, the reaction was neutralized with 1 M tris-HCl (pH 8). The excess peptide was removed during buffer exchange against PBS (pH 7.4) using Microcon centrifugal filters (10-kDa cutoff, MilliporeSigma). The protein was further spun at 13,000*g* for 15 min at 4°C to remove aggregates and filtered before storing at 4°C until further use.

### Generation of pMHC multimer reagents

Here, we generated different multivalent formulations of a given pMHC specificity to enable comparative analysis. To ascribe the observed differences to the multimerization scaffold, all the multivalent pMHC formulations (tetramer, dextramer, and spheromer) were made using the same stock of purified MHC molecules. The pMHC tetramers were generated as described previously (*63*). Briefly, fluorophore-conjugated SAv (Invitrogen) was added to each pMHC monomer incrementally to achieve a 4:1 (pMHC:SAv) molar ratio. Next, SAv agarose was added to each tetramer for quenching any unbound, biotinylated pMHC. After filtration, biotinylated agarose beads were added to remove any unsaturated SAv molecules. The protein was filtered and stored at 4°C until further use. We also used a previously described protocol for generating the pMHC dextramers (*19*). The biotinylated pMHC molecules were incubated with fluorophore-conjugated SAv (Invitrogen) at a molar ratio of ~3.5:1 (pMHC:SAv) for 30 min at room temperature. To this mixture, biotin-dextran (*M*_W_ = 70 kDa, Thermo Fisher Scientific) was added at a molar ratio of ~30:1 (pMHC:dextran) and incubated further for another 30 min at room temperature. The spheromer assembly has already been described above.

### Binding affinity measurements using BLI

Binding affinity for the cognate TCR-pMHC pairs was determined by BLI using an Octet QK instrument (ForteBio). The purified, soluble TCRs were captured onto amine-reactive second-generation (AR2G) biosensors using the amine reactive second-generation reagent kit. The ligand-bound biosensors were then dipped into a decreasing concentration series (50 μM followed by twofold dilutions) of the indicated analytes in PBST to determine the binding kinetics. A series of unliganded biosensors dipped into the analytes served as controls for referencing. In addition, signals from analyte binding to an irrelevant TCR were used for nonspecific binding correction. The traces were processed using ForteBio Data Analysis Software.

### Lentiviral transduction for generating T cell lines

The T cell lines were generated as described previously (*34*). Briefly, gene blocks (Integrated DNA Technologies) corresponding to the TCRα and TCRβ chains of a given TCRαβ pair were cloned into the EF1a-MCS-GFP-PGK-puro lentiviral vector. Each sequence-confirmed (Elim Biopharm) lentiviral plasmid was separately cotransfected with the gag-pol and VSV-G envelope plasmids into Lenti-X 293T cells (Takara Bio) cultured in Dulbecco’s modified Eagle’s medium (Thermo Fisher Scientific) supplemented with 10% fetal bovine serum (FBS, R&D Systems) and penicillin-streptomycin (100 U/ml) using FuGENE (Promega) transfection reagent. After 72 hours, lentiviruses for both TCRα and TCRβ constructs were harvested by collecting the culture supernatant. TCR-deficient Jurkat cells (α^−^β^−^) [American Type Culture Collection (ATCC)] were transduced with the viral supernatant. TCR and CD3 expression was assessed by flow cytometry after staining the cells with anti-TCR α/β (PE, clone 3C10, BioLegend) and anti-CD3 (BV421, clone OKT3, BioLegend) antibodies for 30 min on ice. The cells were washed, resuspended in fluorescence-activated cell sorting (FACS) buffer (PBS with 1% bovine serum albumin and 2 mM EDTA), and acquired on a BD LSRII flow cytometer. The data were analyzed using FlowJo (v10) software. If TCR expression after lentiviral transduction was <80%, enrichment for TCR expression was performed using anti-TCR α/β [allophycocyanin (APC), clone 3C10, BioLegend] antibody in conjunction with anti-APC microbeads (Miltenyi Biotec).

### Binding of T cell lines with pMHC multimers

The binding of pMHC to T cell lines was monitored by flow cytometry. pMHC multimers with Alexa 647–conjugated SAv (Invitrogen) were generated as described above. Binding curves [mean fluorescence intensity (MFI)] were determined using a concentration series of the pMHC multimer reagents. The cells were stained with pMHC multimers (tetramer, dextramer, and spheromer) for 1 hour in FACS buffer. The pMHC multimer staining was done at 4°C or 25°C for MHC-I– and MHC-II–restricted T cell specificities, respectively. The cells were washed and subsequently stained with anti-CD3 (BV421, clone OKT3, BioLegend) antibody for 20 min on ice. The cells were then washed twice, resuspended in FACS buffer, and acquired on the Attune NxT Flow Cytometer (Thermo Fisher Scientific). The data were analyzed using FlowJo (v10) software.

### Human biological sample collection

Peripheral blood mononuclear cells (PBMCs) from healthy donors were obtained from the Stanford Blood Center according to our Institutional Review Board (IRB)–approved protocol. All healthy donor samples used in the current study were confirmed to be HLA-A*02:01^+^ and were collected between April 2018 and February 2019 before the SARS-CoV-2 pandemic. The Epstein-Barr virus (EBV) and HCMV infection status for these donors was also determined by the Stanford Blood Center.

The COVID-19 patient sample collection for this study was conducted at the Stanford Occupational Health under an IRB-approved protocol (protocol director, Kari C. Nadeau). We obtained samples from all COVID-19–positive adults who had a positive test result for the SARS-CoV-2 virus from analysis of nasopharyngeal swab specimens obtained at any point from March to June 2020. Stanford Health Care clinical laboratory developed internal testing capability with a reverse transcriptase–based polymerase chain reaction assay. All participants consented before enrolling in the study. We obtained clinical data from Stanford clinical data electronic medical record system as per consented participant permission. This database contains all the clinical data available on all inpatient and outpatient visits to Stanford facilities. The data obtained included patients’ demographic details, vital signs, laboratory test results, medication administration data, historical and current medication lists, historical and current diagnoses, clinical notes, and radiological results. Participants were excluded if they were taking any experimental medications (i.e., those medications not approved by a regulatory agency for use in COVID-19). The severity of COVID-19 illness was defined on the basis of the symptom score described by Chen *et al.* (*64*).

### PBMC staining and flow cytometry

PBMCs were thawed in a water bath set at 37°C, and the cells were immediately transferred to warm RPMI 1640 medium (Thermo Fisher Scientific) supplemented with 10% FBS (R&D Systems) and penicillin-streptomycin (100 U/ml). After washing, the cells were filtered (70-μm cell strainer) and rested for 1 hour at 37°C. CD8^+^ T cells were enriched from PBMCs by negative selection using a fluorescein isothiocyanate (FITC)–conjugated antibody cocktail against non-CD8^+^ T cells [anti-CD14 (clone HCD14, BioLegend), anti-CD19 (clone HIB19, BioLegend), anti-CD33 (clone HIM3-4, BioLegend), and anti-γδ TCR (clone 5A6.E9, Thermo Fisher Scientific)] followed by magnetic bead depletion using anti-FITC microbeads (Miltenyi Biotec). The enriched CD8^+^ T cells were washed and resuspended in FACS buffer for staining. All pMHC-multimer staining was done for 1 hour at 4°C after incubating the cells with Human TruStain FcX (BioLegend) for 15 min. To compare the frequency of viral (influenza and HCMV) antigen-specific T cells detected using tetramer or spheromer, each sample was divided equally after CD8^+^ T cell enrichment and stained with M1-A*02:01 (Alexa 647) and pp65-A*02:01 (PE) formulated as tetramer or spheromer. The pMHC-multimer formulations were used at a monomeric concentration of 100 nM. The gag-A*02:01 (Alexa 488) pMHC-multimer (200 nM) was used as an irrelevant specificity control. The cells were subsequently stained with anti-CD19 (BV510, clone HIB19), anti-γδ TCR (BV510, clone B1), anti-CD33 (BV510, clone HIM3-4), anti-CD3 (PE/cyanine7, clone OKT3), anti-CD8 (BUV396, clone RPA-T8, BD Biosciences), anti-CD4 (BV785, clone RPA-T4), anti-CCR7 (PE/Dazzle 594, clone G043H7), anti-CD45RA (BV711, clone HI100), and an amine-reactive viability stain (LIVE/DEAD Fixable Aqua Dead Cell Stain Kit; Invitrogen) for 30 min on ice, washed, resuspended in FACS buffer, and acquired on a BD LSRII flow cytometer. All the antibodies for flow cytometry were purchased from BioLegend unless mentioned otherwise. The data were analyzed using FlowJo (v10) software.

For the simultaneous detection of multiple SARS-CoV-2 epitopes (described below) using the spheromer technology, we adapted a combinatorial staining approach developed previously (*49*). Briefly, each peptide was assigned a unique fluorophore barcode that allows the simultaneous detection of 2*^n^* − 1 specificities in a sample, where *n* is the number of distinct fluorophore labels. The relative concentrations for pMHC monomers associated with each fluorophore label (Alexa 647, eFluor 450, PE, and PE/cyanine7) were experimentally determined. Four T cell lines with distinct antigen specificities (M1-A*02:01, pp65-A*02:01, BMLF1-A*02:01, and BHW58-A*02:01) were mixed at a predetermined ratio with TCR-deficient Jurkat cells (α^−^β^−^) and stained with a pool of spheromers, wherein each cognate pMHC was associated with a unique fluorescent tag. The cells were further labeled with anti-CD3 (FITC, clone OKT3, BioLegend) for 30 min, washed, resuspended in flow cytometry buffer, and acquired on a BD LSRII flow cytometer. The data were analyzed to determine the optimal concentration for pMHC monomers associated with each fluorophore label (Alexa 647, 100 nM; eFluor 450, 125 nM; PE, 75 nM; and PE/cyanine7, 50 nM) that provided the maximum separation between the distinct T cell lines. The gag-A*02:01 pMHC-spheromer defined by the fluorophore barcode (Alexa 647 + eFluor 450 + PE + PE/cyanine7) was used as irrelevant specificity control. After staining the PBMC samples with spheromer pools displaying SARS-CoV-2 epitopes, magnetic enrichment of spheromer-positive population was performed using superparamagnetic beads conjugated to an anti–c-myc monoclonal antibody (Miltenyi Biotec). The b2m is engineered to contain an exposed, N-terminal c-myc tag. The cells were subsequently stained with anti-CD19 (BV510, clone HIB19), anti-γδ TCR (BV510, clone B1), anti-CD33 (BV510, clone HIM3-4), anti-CD3 (FITC, clone OKT3), anti-CD8 (BUV396, clone RPA-T8, BD Biosciences), anti-CD4 (BV785, clone RPA-T4), anti-CCR7 (PE/Dazzle 594, clone G043H7), anti-CD45RA (BV711, clone HI100), and an amine-reactive viability stain (LIVE/DEAD Fixable Aqua Dead Cell Stain Kit; Invitrogen) for 30 min. The antigen-specific T cells were enumerated as described previously (*29*, *39*). Briefly, the frequency was calculated on the basis of the total number of pMHC multimer^+^ cells divided by the total CD8^+^ T cells. The absolute counts of the desired cell populations were determined using BD Trucount beads as per the manufacturer’s recommendation (BD Biosciences) by measuring the number of bead events in ^1^/_10_ of the initial staining reaction (pre-enriched) and the eluted fraction after magnetic enrichment. The % recovery after enrichment is estimated by bead count in the eluted fraction. In experiments wherein magnetic enrichment of the pMHC multimer–stained cells was not performed, the entire sample was recorded, and the total cell count of the desired populations determined using BD Trucount beads (BD Biosciences) was used for calculating the frequency of antigen-specific T cells. The sensitivity of pMHC multimer staining after magnetic enrichment was determined by comparing the expected versus the actual numbers of TCR1 cells (BHW58-A*02:01 specificity) recovered from a serial dilution of TCR1 cells into TCR-deficient Jurkat cells (α^−^β^−^). The sensitivity of multimer staining was also determined independently by calculating the recovery of TCR1 cells spiked into PBMCs from a healthy HLA-A*02:01 donor. The TCR1 cells were labeled with a viability dye before spiking them into a PBMC sample. The limit of detection after magnetic enrichment was determined to be ~2 × 10^−7^ (i.e., one antigen-specific T cell in several million total CD8^+^ T cells). About 0.1 × 10^6^ cells from each COVID-19 patient sample were also separately stained (without spheromer pools) with anti-CD19 (BV510, clone HIB19), anti-γδ TCR (BV510, clone B1), anti-CD33 (BV510, clone HIM3-4), anti-CD3 (FITC, clone OKT3), anti-CD8 (BUV396, clone RPA-T8, BD Biosciences), anti-CD4 (BV785, clone RPA-T4), anti-CCR7 (PE/Dazzle 594, clone G043H7), anti-CD45RA (BV711, clone HI100), and anti-HLA-A2 (Alexa 700, clone BB7.2) antibodies and an amine-reactive viability stain (LIVE/DEAD Fixable Aqua Dead Cell Stain Kit; Invitrogen) for 30 min on ice. All the antibodies for flow cytometry were purchased from BioLegend unless mentioned otherwise. The cells were washed, resuspended in FACS buffer, and processed using a BD LSRII flow cytometer. The data were analyzed using FlowJo (v10) software.

### Selection of SARS-CoV-2 peptides and sequence conservation analysis

The complete genome sequence for SARS-CoV-2 isolate SARS-CoV-2/USA/WA-CDC-WA1/2020 (GenBank accession ID: MN985325) was obtained from the National Center for Biotechnology Information (NCBI) database. The binding of all possible 9-nucleotide oligomers from SARS-CoV-2 ORF1ab, S, M, and N proteins to HLA-A*02:01 was predicted following the IEDB recommendations (http://tools.iedb.org/mhci/) (*44*). The peptide binding predictions were cross-validated using the SYFPEITHI algorithms (*45*). We further prioritized peptides based on the biochemical properties of amino acids at positions P2, P5, and P9 (*40*, *46*). The binding of selected peptides to HLA-A*02:01 was further experimentally validated by an MHC stabilization assay using the TAP-deficient T2 cell line (ATCC) expressing HLA-A*02:01. Briefly, T2 cells were incubated with a concentration series of the test peptide (GenScript) in AIM V serum-free medium (Thermo Fisher Scientific) for 1 hour at 37°C. The cells were then transferred to a lower temperature (26°C) for another 14 hours, before returning them to 37°C for 3 hours prior to antibody staining. The cells were washed free of any unbound peptide and incubated with anti–HLA-A2 (PE, clone BB7.2) antibody and an amine-reactive viability stain (LIVE/DEAD Fixable Aqua Dead Cell Stain Kit; Invitrogen) for 30 min on ice. Subsequently, cells were washed, resuspended in FACS buffer, and acquired on a BD LSRII flow cytometer. T2 cells incubated in AIM V serum-free medium alone (no peptide) served as a negative control. The list of SARS-CoV-2 peptides evaluated using the spheromer technology in this study is listed in table S3.

To perform a sequence conservation analysis of the peptides selected from SARS-CoV-2 across other seasonal hCoVs, we obtained representative whole-genome sequences for 229E (HCoV_229E/Seattle/USA/SC0865/2019, GenBank accession ID: MN306046), HKU1 (HCoV_HKU1/SC2628/2017, GenBank accession ID: KY983584), NL63 (HCoV_NL63/UF-2/2015, GenBank accession ID: KX179500), and OC43 (HCoV_OC43/Seattle/USA/SC9430/2018, GenBank accession ID: MN306053) from the NCBI database. The binding of all possible 9-mers from ORF1ab, S, M, and N proteins to HLA-A*02:01 for each of the seasonal hCoV reference strains listed above was predicted following the Immune Epitope Database and Analysis Resource (IEDB) recommendations (http://tools.iedb.org/mhci/). We then filtered the peptides based on percentile rank (<5.0). A lower percentile rank indicates higher affinity. This was done to restrict the search for cross-reactive peptides in hCoVs that are potentially functional owing to their ability to bind HLA-A*02:01, a prerequisite to activate T cells. We then calculated the pairwise sequence similarity score for each of the selected SARS-CoV-2 peptides against all filtered seasonal hCoV peptides using the sequence manipulation suite (*65*). The sequence similarity score was calculated allowing for amino acid substitutions (GA, VLI, FYW, ST, KR, DE, and NQ) with similar biochemical properties (*47*, *48*). The list of seasonal hCoV peptides identified on the basis of the similarity score is given in table S3. The sequence similarity (%) and the percentile rank are also mentioned. The sequences of the SARS-CoV-2 variants of concern for conservation analysis were obtained from the global initiative on sharing avian influenza data (GISAID) database.

### Single-cell paired αβ-TCR sequencing

Multiplexed αβ-TCR sequencing was done following previously established protocols (*33*). In brief, single spheromer^+^ CD8^+^ T cells (for influenza-M1, HCMV-pp65, and SARS-CoV-2 specificities) were sorted into 96-well plates containing 12 μl of OneStep RT-PCR buffer (Qiagen). Reverse transcription was done using the OneStep RT-PCR kit (Qiagen), and the resulting complementary DNA (cDNA) was used for TCRα and TCRβ amplification using multiplex primers. DNA barcodes were also incorporated within the amplified sequences before processing the samples in a single MiSeq2 × 300–base pair sequencing run. The paired sequencing reads were joined, demultiplexed, and mapped to the human TCR reference dataset available at the international ImMunoGeneTics information system as reported previously (*33*).

### Identification of TCR motifs with shared antigen specificity using GLIPH2

We benchmarked the TCR repertoire of antigen-specific (influenza-M1 and HCMV-pp65) CD8^+^ T cells detected using the spheromer by comparing them with tetramer- or dextramer-derived sequences retrieved from the VDJdb (*28*). For each antigen specificity, we implemented the GLIPH2 algorithm to quantify the number of clusters (characterized by a distinct TCR CDR3β motif) that were unique to the spheromer or had an overlap with TCR sequences reported using the tetramer or dextramer. Briefly, the GLIPH2 algorithm compared the antigen-specific TCRs (input dataset) against a reference dataset of 273,920 distinct TCR CDR3β sequences from 12 healthy individuals to generate clusters with unique TCR CDR3β motifs that are significantly enriched (*P* ≤ 0.05, Fisher’s exact test) in the input dataset as previously described (*34*).

We also analyzed the SARS-CoV-2 epitope–specific TCR sequences identified from unexposed, healthy individuals using the spheromer by implementing the GLIPH2 algorithm. The TCR sequences from COVID-19 patient samples for this analysis were obtained from a published dataset (*66*). The inclusion of multiple statistical measurements in the GLIPH2 output accounting for Vβ gene usage biases, CDR3β length distribution (relevant only for local motifs), cluster size, HLA allele usage, and clonal expansion facilitates the calling of high-confidence specificity groups.

### In vitro stimulation of T cell lines

The stimulation assay was done as previously described (*62*). The assay was set up in 96-well clear round bottom microplates (Corning) with a volume of 200 μl during all incubation steps. T2 cells expressing HLA-A*02:01 were plated at a density of 50,000 cells per well in Iscove’s modified Dulbecco’s medium (Thermo Fisher Scientific) supplemented with 10% FBS (R&D Systems) and penicillin-streptomycin (100 U/ml) and pulsed with 100 mM of the test peptide for 3 hours at 37°C. The cells were then washed and cocultured with Jurkat cells expressing an exogenous TCR of interest (100,000 cells per well) in RPMI 1640 medium (Thermo Fisher Scientific) supplemented with 10% FBS (R&D Systems) and penicillin-streptomycin (100 U/ml) for 16 hours. The next day, the cells were washed with FACS buffer and stained with anti-CD3 (APC, clone OKT3) and anti-CD69 (PE, clone FN50) antibodies for 20 min at 4°C. Cells were washed, resuspended in FACS buffer, and analyzed on the Attune NxT Flow Cytometer (Thermo Fisher Scientific). The data were analyzed using FlowJo (v10) software.

### Statistical analysis

R statistical package was used to perform the Fisher’s exact test to compute TRBV gene enrichment across different pMHC formulations using the fisher.test function. Fisher’s exact test was also used to determine the significance levels of the distribution of GLIPH2 TCR motifs at different World Health Organization (WHO) scores identified using peptides either unique to SARS-CoV-2 or conserved across hCoVs. Next, we performed a meta-analysis to combine the *P* values from individual hypothesis tests to assess the significance of the overall distribution. Dimensionality reduction analysis was also performed in R. Uniform Manifold Approximation and Projection (UMAP) to visualize multiparametric flow cytometry data was generated using the “umap” package. Additional data and statistical analyses were done in GraphPad Prism. The statistical details for each experiment are provided in the associated figure legends.

## Supplementary Material

20210701-1

## Supplementary Materials

### Other Supplementary Material for this manuscript includes the following:

Data file S1

MDAR Checklist

## Notes

View/request a protocol for this paper from *Bio-protocol*.
