# Supplementary material for: CD8+ T cells specific for conserved coronavirus epitopes correlate with milder disease in patients with COVID-19
Source: Sci Immunol. 2021 Jul 1;6(61):eabg5669. doi: 10.1126/sciimmunol.abg5669 (PMC8975171; doi:10.1126/sciimmunol.abg5669)
Supplement: 20210701-1 [file sciimmunol.abg5669.v1.pdf]

Cite as: V. Mallajosyula et al., Sci. Immunol.
10.1126/sciimmunol.abg5669 (2021).

CORONAVIRUS

CD8+ T cells specific for conserved coronavirus epitopes correlate with milder disease in COVID-19 patients

Vamsee Mallajosyula[1], Conner Ganjavi[2], Saborni Chakraborty[3], Alana M. McSween[1], Ana Jimena Pavlovitch-Bedzyk[4], Julie Wilhelmy[1], Allison Nau[1], Monali Manohar[5], Kari C. Nadeau[1,5], Mark M. Davis[1,6,7]*

[1]Institute for Immunity, Transplantation, and Infection, Stanford University School of Medicine, Stanford, CA 94305, USA. [2]Department of Biology, Stanford University School of Humanities and Sciences, Stanford, CA 94305, USA. [3]Department of Medicine, Division of Infectious Diseases, Stanford University, Stanford, CA 94305, USA. [4]Computational and Systems Immunology Program, Stanford University School of Medicine, Stanford, CA 94305, USA. [5]Sean N. Parker Center for Allergy and Asthma Research, Stanford University and Division of Pulmonary, Allergy, Critical Care Medicine, Stanford University School of Medicine, Stanford, CA 94305, USA. [6]Department of Microbiology and Immunology, Stanford University School of Medicine, Stanford, CA 94305, USA. [7]Howard Hughes Medical Institute, Stanford University School of Medicine, Stanford, CA, 94305, USA.

*Correspondence to: mmdavis@stanford.edu

A central feature of the SARS-CoV-2 pandemic is that some individuals become severely ill or die, whereas others have only a mild disease course or are asymptomatic. Here we report development of an improved multimeric αβ T cell staining reagent platform, with each maxi-ferritin "spheromer" displaying 12 peptide-MHC complexes. Spheromers stain specific T cells more efficiently than peptide-MHC tetramers and capture a broader portion of the sequence repertoire for a given peptide-MHC. Analyzing the response in unexposed individuals, we find that T cells recognizing peptides conserved amongst coronaviruses are more abundant and tend to have a "memory" phenotype, compared to those unique to SARS-CoV-2. Significantly, CD8+ T cells with these conserved specificities are much more abundant in COVID-19 patients with mild disease versus those with a more severe illness, suggesting a protective role.

INTRODUCTION

Severe acute respiratory syndrome coronavirus 2 (SARS-CoV-2), the virus causing COVID-19, has infected ~120 million individuals worldwide, displaying a spectrum of disease severities that ranges from asymptomatic to life-threatening pneumonia and multi-organ failure (*1*). Addressing this global pandemic, many pharmaceutical companies and research laboratories have raced to develop effective coronavirus vaccines, of which over a hundred are in development (*2*). The primary goal of most vaccine development efforts is the generation of neutralizing antibodies targeting the SARS-CoV-2 spike (S) protein. However, the variable magnitude and durability of these antibody responses in COVID-19 patients highlights the importance of studying T cell mediated immunity to better understand disease pathogenesis and to develop benchmarks for an effective T cell response (*3*–*6*). Many studies have shown that T cells are involved in a SARS-CoV-2 infection (*7*–*13*), but what types of responses are efficacious, and which are not is unclear.

The majority of T cells in most mammals, including human beings, express the αβ T cell receptor (TCR) and recognize a particular peptide bound to a major histocompatibility complex molecule (pMHC) expressed on target cells (*14*). The weak equilibrium dissociation constant (K_D ~1-200μM) between the TCR and monomeric pMHC results in a transient complex that impedes easy detection (*15*). The development of the pMHC-tetramer ("tetramer") technology, wherein conjugation of four pMHC molecules to streptavidin (SAv) results in the increased avidity of TCR binding, laid the foundation to circumvent this problem (*16*). Since then, several studies have increased the valency of pMHC multimers to improve these reagents' ability to detect T cells with marginal affinity (*17*–*19*), such as pMHC dextramers that use dextran polymers to increase the number of pMHC. However, the detection of low affinity TCRs still remains challenging, partly due to an increased background from non-specific staining using higher valency platforms, thus negatively impacting the signal-to-noise ratio (*19*–*21*).

In order to improve upon these limitations, we engineered a biotinylation site on maxi-ferritin to create a 24-subunit, self-assembling protein scaffold for the multivalent display of pMHC. This "spheromer" platform offers several advantages: ease of production, defined site-specific conjugation of pMHC molecules that significantly reduces inter-batch variation, and compatibility with currently available pMHC molecules and streptavidin reagents allowing for facile translation. We show that the spheromer binds both MHC-I and MHC-II restricted T cells with excellent specificity for pMHC, and at a significantly higher avidity than the tetramer. Furthermore, this reagent provides a better signal-to-noise ratio and

detects a much more diverse antigen-specific TCR repertoire in comparison to equivalent tetramers or dextramers. Finally, using the spheromer for direct ex vivo study of SARS-CoV-2 specific $CD8^+$ T cells, we show that T cells predicted to cross-react with seasonal human coronaviruses are significantly enriched in COVID-19 patients with mild symptoms in comparison to individuals with severe disease. Since there is evidence that antibodies to SARS-CoV-2 begin to wane not long after infection (*3*, *5*), these robust T cells to conserved epitopes detected in SARS-CoV-2 unexposed individuals and in those with mild disease could be the key determinant in a successful adaptive immune response and could help to explain the disparity in COVID-19 outcomes. Furthermore, following these T cells using spheromer technology could help in tracking SARS-CoV-2 immunity in vaccinated individuals, especially in the context of emerging SARS-CoV-2 mutant strains that in some cases escape vaccine-induced antibody responses (*22*).

RESULTS

In the search for a protein scaffold that could increase the valency of displayed pMHC and that would hopefully capture more αβ T cells of a given specificity, we focused on self-assembling homo-oligomers **(Fig. S1A)** (*23*, *24*). Based on the yield and homogeneity of the recombinantly expressed proteins **(Fig. S1B-C)**, we chose maxi-ferritin for further optimization. Ferritins are naturally occurring cage proteins that participate in biomineral synthesis and are found across almost all living organisms (*23*). Studies have shown that thermophilic proteins denature at a much higher temperature than their mesophilic homologs (*25*). Therefore, we used ferritin derived from the hyperthermophilic archaeal anaerobe *Pyrococcus furiosus* to develop a stable scaffold. Maxi-ferritin forms a 24-subunit nanoparticle with an external diameter of ~120Å. In order to develop a platform that is widely accessible, we functionalized the maxi-ferritin scaffold to be compatible with components of the existing tetramer technology that uses biotinylated pMHC monomers and SAv conjugates. We inserted a biotinylation signal sequence (*26*) at the N terminus of each maxi-ferritin subunit (~23kDa monomer) and utilized SAv as a ‘molecular glue’ to bring together pMHC monomers and the scaffold **(Fig. 1A-C)**. We optimized the tethers for SAv on the maxi-ferritin scaffold by testing a set of linkers that spanned a diverse range of lengths and molecular rigidities **(Fig. S2A-B)** (*27*). As shown, the optimized scaffold with radially projecting tethers could be purified easily and functionalized with biotin **(Fig. 1D)**. We then bound the biotinylated scaffold to SAv conjugated to two peptide-MHC molecules ($SAv\text{-}pMHC_2$: semi-saturated SAv) **(Fig. 1E-F)**. The $SAv\text{-}pMHC_2$ precursor formation is not impacted significantly by different fluorophores conjugated to SAv, even though PE and PE/Cyanine7 for instance are much larger molecules than Alexa 488, eFluor 450 and Alexa 647 **(Fig. S3A)**. The semi-saturated SAv has two biotin binding sites available to bind the scaffold. Upon saturation, we observed the display of 12 pMHC molecules as determined by size-exclusion chromatography (SEC) and Blue native PAGE (BN-PAGE) **(Fig. S4A-B)**. The current iteration does not allow the conjugation of more pMHC molecules, presumably because of steric hindrance. It is also possible that two adjacent biotinylated linkers on the scaffold are being occupied by a single $SAv\text{-}pMHC_2$ molecule. We further purified the homogeneous spheromer by size-exclusion chromatography to exclude the contribution from any unreacted $SAv\text{-}pMHC_2$ **(Fig. 1G)**. We also validated the conjugation of $SAv\text{-}pMHC_2$ onto the functionalized maxi-ferritin scaffold using negative-stain EM **(Fig. 1H)** and ELISA **(Fig. 1I-J)**. Another objective during the extensive linker (L1-L19) design phase was to optimize the radial projection of the biotin tethers from the maxi-ferritin scaffold to identify a construct (L6: $(SG_2P)_2SG_2$) that is least impacted by different fluorophores. As shown, all the spheromers assembled using the optimized maxi-ferritin scaffold (displaying the L6-linker) and five different fluorophore-conjugated SAv formed a homogeneous complex in solution **(Fig. S3B-E)**.

We characterized the general applicability of the spheromer using a set of TCR-pMHC pairs with distinct TRBV usage, antigen sources, and examples representing both MHC-I and MHC-II molecules **(Fig. 2A)**. The binding of TCR with different formulations of their cognate pMHC (monomer, tetramer and spheromer) was determined using biolayer interferometry **(Figs. 2B-C and S5A-B)**. Encouragingly, the spheromer bound all the evaluated TCRs significantly better than the other formulations (monomer and tetramer). On average, for MHC-I restriction, the spheromer bound TCRs with >250 (monomer) and >50-fold (tetramer) greater net-affinity. For MHC-II restricted TCRs, the spheromer bound with >200 (monomer) and >20-fold (tetramer) greater net-affinity across the tested pairs. We also generated stable T cell lines to compare the binding of different pMHC formulations (tetramer, dextramer and spheromer) using flow cytometry **(Figs. 3A-F and S6A-F)**. As shown, for all the evaluated pMHC-TCR pairs, consistent with the increased avidity, the signal from spheromer staining was significantly better (~10-fold) than the tetramer. We included negative controls ($TCR^{-/-}$ Jurkat cells and a cell line expressing irrelevant TCR) to determine background staining since higher valency can result in noise amplification due to non-specific interactions (*19*, *20*). We observed that while there was an increase in staining intensity with dextramer staining (~6-fold) in comparison to the tetramer, the background staining was also higher. In contrast, the background staining with the spheromer did not increase substantially, resulting in a better signal-to-noise ratio compared to other pMHC-formulations **(Figs. 3C, F and S6C, F)**. This

difference is likely because the spheromer is a discrete, homogenous structure versus a mix of dextran polymers in the dextramer reagents. The spheromer stains better than the tetramer irrespective of the conjugated fluorophore **(Fig. S7A-B)**. A fluorophore conjugated maxi-ferritin scaffold can also be alternatively used for assembling the spheromer with unlabeled SAv-pMHC$_2$ **(Fig. S7C)**.

Next, we evaluated viral-specific CD8$^+$ T cells in healthy individuals to address the following questions: i) Does the spheromer detect a higher frequency of antigen-specific T cells than tetramer ex vivo? ii) How do the TCR repertoires detected by the spheromer and tetramer compare? We used immunodominant HLA-A*02:01 restricted epitopes (influenza-M1 and HCMV-pp65) for characterizing the spheromer since there is considerable data available for benchmarking (*28*). CD8$^+$ T cells isolated from each donor (n=7) were divided evenly for tetramer or spheromer staining **(Figs. 4A-B and S8A-C)**. The frequencies of antigen-specific T cells detected using tetramer are consistent with previous studies (*29*–*32*). As shown, a significantly higher frequency of antigen-specific CD8$^+$ T cells could be detected for both M1 ($p = 0.015$) and pp65 ($p = 0.016$) viral specificities **(Figs. 4C-D and S8D)**. As expected, the frequency of antigen-specific CD8$^+$ T cells in HCMV-negative donors was significantly lower than those in HCMV-positive donors **(Fig. S8E)**. We also validated spheromer staining using biotinylated A*02:01 pMHC monomers procured from the NIH tetramer core facility, which is a major source of tetramer reagents to the research community worldwide **(Fig. S9A-D)**. Next, we single-cell sorted spheromer$^+$ CD8$^+$ T cells and performed paired $\alpha\beta$-TCR sequencing to study the repertoire (*33*). The spheromer-derived TCR sequences were analyzed against TCR entries in VDJdb, a curated database of TCRs with known antigen specificities (*28*). We compared the TRBV usage of TCR sequences obtained using distinct pMHC formulations **(Fig. 4E, G)**. Overall, we observed that the spheromer detected a much more diverse repertoire in comparison to either the tetramer or dextramer. As shown, the M1-specific TCR sequences detected with the spheromer had a significantly (p-value < 0.01, Fisher's test) higher usage of 5 and 3 TRBV genes in comparison to the tetramer- and dextramer-derived sequences, respectively, with 2 overlapping genes (TRBV12-3 and TRBV28) across them **(Fig. 4E)**. Similarly, spheromer$^+$ pp65 TCR sequences showed an enrichment of 4 TRBV genes in comparison to the tetramer and 1 TRBV gene with the dextramer **(Fig. 4G)**. Intriguingly, TRBV6-5 is significantly enriched in tetramer$^+$ pp65$^+$ TCR sequences when compared to both the dextramer and spheromer-derived sequences. We further analyzed the specificity of spheromer-derived TCR sequences using GLIPH2 (grouping of lymphocyte interaction by paratope hotspots), an algorithm that clusters TCRs based on shared antigen specificity **(Fig. 4F, H)** (*34*). Globally, we observed a significant overlap (~91%) between the TCR 'motifs' identified using spheromer and antigen-specific TCR entries in VDJdb. The recovery of previously characterized antigen-specific TCR motifs using the spheromer provides further confirmation that our designed platform is indeed detecting relevant T cells. The spheromer could detect previously described public TCRs for both M1 (CDR3b: CASSIRSSYEQYF, CASSIRSAYEQYF) and pp65 (CDR3b: CASSYQTGASYGYTF) viral specificities shown to have a significant association with HLA-A*02:01 (*35*, *36*). Interestingly, the spheromer identified a set of TCR motifs that did not cluster with sequences previously reported in VDJdb (8% for M1 and 9% for pp65). In order to test whether these TCRs could confer reactivity to the pMHCs they were selected with, we generated T cell lines with TCRs from these previously unidentified GLIPH2 clusters **(Figs. 4I, K and S10A, C)**. As shown using CD69 expression, these T cell lines could be activated specifically using the cognate peptide **(Figs. 4J, L and S10B, D)**. We also measured the TCR binding of these clones to their cognate pMHC monomers by biolayer interferometry. As shown, TCRs detected exclusively using the spheromer on average bound the pMHC monomer with ~30-fold lower affinity in comparison to previously reported reference TCRs (*37*, *38*) **(Figs. 4M-N and S10E-F)**. These results demonstrate that spheromer reagents are not just more efficient at staining the relevant T cells but can also identify low-affinity antigen-specific T cells that may not be detected with other multimer reagents.

To address the immune response to SARS-CoV-2, we made spheromer reagents to evaluate CD8$^+$ T cell responses in unexposed individuals and COVID-19 patients. We have previously shown that T cells to viral epitopes can be detected in the peripheral blood of naïve individuals (*39*, *40*). Significantly, a large fraction (~50%) of these T cells in adults (28-80y) exhibited a memory phenotype, possibly due to higher TCR cross-reactivity or environmental exposures (*39*). The rapid recruitment of these T cells in an immune response could offer a survival advantage, since clonal expansion and the induction of memory lymphocytes is a key goal of vaccination efforts and strongly correlates with protection against particular infectious diseases. Previous studies have also shown that T cell precursor frequencies correlate with the magnitude of anti-viral responses (*41*–*43*). Therefore, we determined the frequency of CD8$^+$ T cells against a panel of SARS-CoV-2 epitopes **(Fig. 5A)** in naïve, unexposed individuals using the spheromer. The peptides were selected from multiple SARS-CoV-2 open reading frames (ORFs) spanning ORF1ab, S, M and N proteins **(Table S1)**. The peptides (9-mers) evaluated in this study were chosen based on the predicted binding affinity to HLA-A*02:01 determined using the immune epitope database and analysis resource (IEDB) recommendations (http://tools.iedb.org/mhci/) (*44*) and cross-

validated using the SYFPEITHI algorithms (*45*). Furthermore, the biochemical properties of amino acids at positions P2, P5 and P9 were given higher weights (*40*, *46*). We used an MHC stabilization assay to further validate the binding of peptides to A*02:01 MHC-I molecules expressed on the antigen processing (TAP) deficient T2 cell line (**Fig. S11**). We also designed our peptide panel to represent a diverse range of sequence similarities with peptides from common cold-causing human coronaviruses (hCoV-OC43, HKU1, 229E, NL63) to evaluate cross-reactive responses. The amino acid substitution matrix to determine sequence conservation was chosen based on previous studies (*47*, *48*) to prioritize SARS-CoV-2 T cell epitopes, but it must be noted that exceptions defined by an idiosyncratic TCR cross-reactivity profile will exist. We used a combinatorial staining approach as described previously to simultaneously probe for multiple specificities in a single sample followed by magnetic enrichment of antigen-specific $CD8^+$ T cells (**Fig. S12**) (*49*). In unexposed individuals, we observed that a few SARS-CoV-2 epitopes (P5, P10, P12, P13, P17 and P18) had an elevated $CD8^+$ T cell frequency ($2.07\times10^{-4} \pm 1.16\times10^{-4}$) when compared to other peptides ($2.96\times10^{-5} \pm 2.01\times10^{-5}$) in the panel (**Fig. 5B-C**), albeit at lower levels than the frequency of T cells against "immunodominant" epitopes of other viruses (HCMV and influenza) (**Fig. 5C**). We determined the limit of detection after magnetic enrichment to be ~2´10^{-7}. We experimentally validated the cross-reactivity between a subset of SARS-CoV-2 and seasonal hCoV epitopes (**Fig. 6A-B**). Generally, the epitopes to which we observed elevated T cell frequencies in unexposed individuals were characterized by high sequence similarity with hCoVs (**Fig. 6C**). TCR sequencing of $CD8^+$ T cells from unexposed individuals identified using spheromers presenting SARS-CoV-2 epitopes showed that T cells against peptides conserved across coronaviruses are relatively expanded in comparison to T cells against peptides unique to SARS-CoV-2 (**Fig. 6D-E**). Phenotypic characterization of these antigen-specific T cells using CCR7 and CD45RA markers showed a distinct distribution between the naïve/memory compartments for the tested peptides (**Fig. 7A-C**). T cells detected with peptides having low hCoV sequence similarity demonstrated a predominantly naïve phenotype. In contrast, peptides against which relatively elevated T cell frequencies were observed in unexposed individuals showed a memory phenotype (~80%) and correlated with high hCoV sequence similarity. This suggests that exposure to seasonal hCoVs among other cross-reactive environmental exposures could contribute to the observed expansion of these T cells. Next, we determined the $CD8^+$ T cell frequencies against these SARS-CoV-2 epitopes in COVID-19 patients presenting mild or severe symptoms. We observed that in addition to the spike protein (S: n = 4/6), $CD8^+$ T cells against epitopes from other SARS-CoV-2 proteins (ORF1ab: n = 3/13, M: n = 2/4 and N: n = 1/2) were also present at a significantly higher frequency in COVID-19 patients (mild/severe) when compared to unexposed individuals (**Fig. 8A-D**). Intriguingly, we observed that $CD8^+$ T cell frequencies to specific epitopes were significantly different comparing mild and severe COVID-19 patients. In general, the peptides which showed a higher response in severe patients had a lower similarity to other hCoVs. In contrast, patients exhibiting mild symptoms showed an elevated response to peptides with relatively higher sequence similarity to other hCoVs (**Fig. 8E**). Using GLIPH2, we could identify TCR motifs shared between unexposed individuals and COVID-19 patients (**Fig. 8F**). TCR motifs against conserved epitopes are enriched in COVID-19 patients with mild symptoms. In contrast, TCR motifs characterizing severe COVID-19 patients were detected using peptides that were primarily unique to SARS-CoV-2 (adjusted p-value = 0.00019, Fisher's test). A high fraction of these antigen-specific $CD8^+$ T cells enriched in mild COVID-19 patients displayed an effector phenotype indicating recent antigen activation (**Fig. 8G-I**). This suggests that T cells found in unexposed individuals that bind SARS-CoV-2 epitopes could be actively recruited during infection. Overall, our data suggest a preferential recruitment of memory $CD8^+$ T cells specific for conserved epitopes, that are likely the result of previous hCoV exposures in COVID-19 patients developing mild symptoms.

DISCUSSION

Antigen-specific T cell responses are known to be essential for an effective immune response against many infectious diseases but defining specific benchmarks for what is protective versus what is not has been challenging, especially in human studies (*50*, *51*). This is due to many factors, including the low frequency of disease-relevant T cells, particularly when clinical samples are limiting, as they typically are. Consequently, some methods used to investigate T cells necessitate expansion of cells in culture which may alter the relative abundance and phenotype of some T cell clonotypes. Also, the TCR repertoire cannot be studied with some of these methods due to their incompatibility with sequencing techniques. The development of tetramer technology partially addressed this limitation and enabled the direct measurement and characterization of T cells ex vivo. Subsequent advances, both in terms of reagents and methods, have widened the scope of applications (*17*–*19*, *49*, *52*–*56*). However, the detection of low-affinity T cells is still lacking in many cases (*18*).

Here, we report the development of a multivalent 'spheromer' system built on the scaffold of a self-assembling maxi-ferritin nanoparticle. As shown, the system has been engineered to be compatible with current pMHC (both MHC-I and MHC-II molecules) and SAv reagents that allows ease-of-use. The optimized spheromer assembly pipeline resulted in a very consistent reagent across multiple batches of synthesis with a relative ease of production, unlike the dodecamer (*19*).

The defined geometry of the scaffold facilitated precise site-directed conjugation of pMHC, leading to a relatively homogenous reagent as assessed using a size-exclusion column. The spheromer bound cognate TCRs with a significantly higher avidity when compared to the tetramer, for both MHC-I (>50-fold) and MHC-II (>20-fold) molecules. Also, the low background contributed to the better signal-to-noise ratio observed in comparison to other pMHC-formulations tested. The improved TCR-binding properties of the spheromer may also be in part due to better 2D binding kinetics owing to its larger diameter. This may provide a better surrogate than either the tetramer or dextramer for membrane-embedded pMHC molecules that engage TCRs in vivo. This increased avidity and specificity can potentially enable the detection of more disease relevant, low-affinity T cells. Using the HLA-A*02:01-restricted influenza-M1 and HCMV-pp65 epitopes, we demonstrated that a significantly higher frequency of antigen-specific CD8+ T cells with a much more diverse TCR repertoire could indeed be detected with the spheromer. These results demonstrate that our engineered scaffold can be readily adapted with currently available reagents without a time-consuming systemic overhaul.

We further applied the spheromer technology to delineate the CD8+ T cell response to SARS-CoV-2 using a panel of peptides derived from multiple proteins (ORF1ab, S, M and N) that were validated for HLA-A*02:01 binding. Studies have shown that a T cell response can indeed be generated against multiple SARS-CoV-2 proteins (*7–13*). We observed a relatively higher frequency of T cells against a few epitopes in the ORF1ab (P5, P10, P12, and P13) and S (P17 and P18) proteins in naïve, unexposed individuals. The high sequence similarity of these epitopes to hCoVs and the predominant memory phenotype of these T cells suggests that exposure to seasonal coronaviruses could contribute to the expansion of potentially cross-reactive T cells. Importantly, the frequency of T cells against a subset of these cross-reactive peptides (P5, P10, P12 and P17) was significantly higher in COVID-19 patients with mild symptoms. In contrast, T cells to unique ORF1ab derived peptides (P1 and P8) were higher in severely ill COVID-19 patients. These peptides (P1 and P8) have low sequence similarity to hCoVs. Overall, our data indicate that mild and severe COVID-19 patients elicit distinct T cell responses to particular SARS-CoV-2 epitopes. Also, the preferential recruitment of memory CD8+ T cells to cross-reactive epitopes likely contributes to their mild symptoms. These cross-reactive T cell responses need to be investigated in children as they may contribute to their milder clinical symptoms when compared to adults (*57*) since seasonal hCoVs infections are more frequent in children than adults (*58*). This study suggests that in addition to pre-existing cross-reactive memory CD4+ T cells reported previously (*10*), dissimilar SARS-CoV-2 epitope-specific CD8+ T cell responses could also contribute to divergent COVID-19 clinical outcomes. The observation of CD8+ T cell responses to multiple SARS-CoV-2 proteins is consistent with previous studies. Accordingly, the data presented here suggests that the incorporation of additional non-spike epitopes into a vaccine could further bolster anti-viral T cell immunity. This can be important given the emergence of several SARS-CoV-2 variants of concern (https://www.cdc.gov/coronavirus/2019-ncov/cases-updates/variant-surveillance/variant-info.html). Sequence analysis of SARS-CoV-2 epitopes found to be associated with mild symptoms in our study across variants indicates that one of the two spike protein epitopes (P17: VLNDILSRL) has mutated (S®A) in the B.1.1.7 lineage variants circulating in Europe. In contrast, none of the non-spike protein epitopes associated with mild symptoms were mutated across the analyzed variants (*59*, *60*).

Overall, this study demonstrates the potential of the spheromer technology but is limited in terms of the specificities and samples used for comparing the different pMHC-multimer platforms. Extending these results to other class I and class II HLA alleles will be important in the future, but the results shown here are consistent across different antigens complexed to HLA-A*02:01 and in our experience it would be surprising if it wasn't advantageous to use this platform for other HLA alleles as well.

MATERIALS AND METHODS

Study design

The objective of this study was to measure cross-reactive CD8+ T cell immunity between seasonal coronaviruses that cause the common cold and SARS-CoV-2. We measured the frequency of antigen-specific T cells in unexposed pre-pandemic donors and COVID-19 patients presenting mild or severe symptoms to evaluate the contribution of pre-existing immunity to seasonal coronaviruses in disease resolution. For direct, ex vivo detection of antigen-specific T cells at single epitope resolution, we developed an improved multimeric αβ T cell staining "spheromer" reagent.

Design, expression and characterization of multimeric protein scaffolds

In order to develop an optimized self-assembling protein scaffold for the multivalent presentation of peptide-MHC (pMHC) molecules, we designed and tested several (n > 30) protein constructs. All constructs were codon-optimized for expression in mammalian cells. Gene blocks (Integrated DNA Technologies) corresponding to individual constructs were cloned into a vector with a CMV/R promoter by Gibson assembly (New England Biolabs) and sequence confirmed (Elim Biopharm).

We first evaluated the heterologous recombinant expression of self-assembling proteins with different oligomeric states (n = 12, 24 and 60). The sequences corresponding to

mini-ferritin (12-mer, UniProt accession ID: P0ABT2), maxi-ferritin (24-mer, UniProt accession ID: Q8U2T8) and lumazine synthase (60-mer, UniProt accession ID: E6PLJ8) were cloned and expressed in Expi293F cells (Thermo Fisher Scientific) as per the manufacturer recommendations. Briefly, 100ml of Expi293F cells sub-cultured at a density of 3×10^6 viable cells/ml in Expi293 expression media (Thermo Fisher Scientific) were transfected with the expression plasmids complexed with ExpiFectamine 293 transfection reagent. Next day (~18-22h post-transfection), the cells were supplemented with a cocktail of enhancers. The cell cultures were further incubated for 4 days. Subsequently, the culture supernatants were harvested by centrifugation (2000×g, 30 min, 4°C) for protein purification. The supernatants were filtered (0.45mm PES membrane filters, Thermo Fisher Scientific) and diluted with 20 mM Tris-HCl, pH 8. The proteins were bound to a HiTrap Q FF anion exchange column (Cytiva) using an AKTA pure 25 L1 system (Cytiva). An NaCl gradient (in 20 mM Tris-HCl, pH 8) was used to elute the bound proteins. The yield and purity of the multimeric protein scaffolds was estimated using a NuPAGE Bis-Tris 4-12% gradient gel system (Thermo Fisher Scientific). The homogeneity of the purified proteins was assessed using size-exclusion columns (Superdex 200 Increase 10/300 GL, Superose 6 Increase 10/300 GL (Cytiva)) that were calibrated using a wide range of molecular weight standards (Bio-Rad).

On the basis of protein yield and homogeneity, we further optimized the maxi-ferritin scaffold for pMHC display by testing multiple linkers varying in length and rigidity. A list of all the evaluated linkers is given in Fig. S2A. Each construct was expressed in mammalian cells and purified as described above. The protein construct with linker $(SG_2P)_2SG_2$ (L6) was chosen for "spheromer" assembly based on yield and optimal radial projection from the scaffold. The sequence of the optimized maxi-ferritin scaffold is given in Fig. S2B. Site-directed functionalization (biotinylation) of the scaffold was performed using BirA biotin-protein ligase. The purified scaffold was incubated with components of the biotinylation reaction as per the manufacturer's recommendation (Avidity). The functionalized scaffold was subsequently separated from free biotin using a Superdex 200 Increase 10/300 GL (Cytiva) size-exclusion column. Next, the efficiency of protein biotinylation was assessed using a streptavidin gel-shift assay. Briefly, the protein was boiled at 90°C for 7 min before incubation on ice for 10 min. Subsequently, a 2-fold molar excess of streptavidin (SAv, Agilent) was added to the protein and incubated further for an additional 10 min on ice. The shift in mobility of the scaffold resulting from SAv binding was evaluated using the NuPAGE Bis-Tris 4-12% gradient gel system (Thermo Fisher Scientific).

Spheromer assembly and characterization

The spheromer assembly is a two-step process: i) Generation of a semi-saturated SAv-$pMHC_2$ complex, and ii) Conjugation of SAv-$pMHC_2$ to the functionalized maxi-ferritin scaffold. We optimized the reaction conditions for getting the maximum yield of SAv-$pMHC_2$ by varying the reactant concentrations, incubation time, agitation conditions and reaction temperature. We evaluated the formation of SAv-$pMHC_2$ by size-exclusion chromatography (Cytiva) and NuPAGE Bis-Tris 4-12% gradient gel system (Thermo Fisher Scientific). The maximum yield of SAv-$pMHC_2$ was obtained by incubating 1 μM of the pMHC (monomer) with 0.45 μM of SAv at 25°C for 30 min without agitation. Subsequently, the spheromer complex was assembled by incubating SAv-$pMHC_2$ with the functionalized scaffold for 1h at room temperature with mild rotation. The unconjugated and fluorophore-conjugated SAv were sourced from Agilent and Invitrogen, respectively. We determined the stoichiometry of pMHC saturation on the spheromer by incubating the functionalized scaffold with increasing concentrations of SAv-$pMHC_2$ and analyzing the resulting product on size-exclusion columns calibrated using a broad range of molecular weight standards. The complexes were also assessed by Blue native PAGE (BN-PAGE) as per the manufacturer recommendations (Thermo Fisher Scientific). We further purified the spheromer assembly using a size-exclusion column to mitigate the confounding effects from any unreacted SAv-$pMHC_2$.

We also validated the conjugation of pMHC onto the functionalized scaffold by negative stain electron microscopy. 5μl of the purified samples (0.005-0.5 mg/ml) was applied on glow discharged carbon-coated grids, blotted and stained with 1% uranyl formate according to standard protocols (*61*). Negative stained grids were imaged on an FEI Morgagni at 100kV.

The number of pMHC molecules conjugated to the engineered maxi-ferritin scaffold was also quantified by ELISA using standard curves generated for pMHC and SAv. Briefly, test samples were coated on 96-well Nunc plates (Thermo Fisher Scientific) at 2 mg/ml in 50 μl PBS, pH 7.4 at 37°C for 1 hour. Plates were then washed with PBS containing 0.05% Tween-20 (PBST) and blocked with 3% skim milk in PBST for 1h. The plates were washed and incubated at room temperature with 50 μl of HRP-conjugated anti-streptavidin IgG (Abcam) in blocking buffer at a predetermined dilution (1:5000) for 1h for the detection of SAv. Alternatively, MHC-I and MHC-II molecules were detected using HRP-conjugated anti-human HLA-A2 antibody (LSBio) or HRP-conjugated anti-human HLA-DR antibody (LSBio). Plates were washed with PBST and developed with 75 μl/well of the substrate 3,3′,5,5′-tetramethylbenzidine (TMB) solution (MilliporeSigma). The reaction was stopped with 100 μl/well of ELISA stop solution for TMB (Thermo Fisher Scientific). The optical density at 450 nm was measured using the FlexStation 3 Multi-Mode Microplate Reader (Molecular Devices) and

corrected for any non-specific background signal from ovalbumin coated wells.

Cloning, expression and purification of soluble TCRs

The soluble TCRs were expressed and purified as described previously (*62*). Briefly, for each TCR, the extracellular domains corresponding to the TCRα and TCRβ chains were codon-optimized for expression in insect cells and cloned independently into a baculovirus expression vector optimized for TCR expression by Gibson assembly (New England Biolabs). The sequence confirmed (Elim Biopharm) plasmids were amplified in *E.coli* (New England Biolabs). Each plasmid was co-transfected with BestBac Linearized Baculovirus DNA (Expression Systems) into Sf9 insect cells (Expression Systems) using Cellfectin II for the production of baculoviruses. The P1 stocks of TCRα and TCRβ baculoviruses of a given TCRαβ pair were titrated to ensure a 1:1 TCRαβ hetero-dimer formation and then co-transduced into High Five cells (Thermo Fisher Scientific). After 3 days, the supernatant was collected by centrifugation. A precipitation mix (50 mM Tris-HCl (pH 8), 1 mM $NiCl_2$, and 5 mM $CaCl_2$) was added to the supernatant while stirring for 15 min at 25°C. The precipitation was subsequently removed by centrifugation and the supernatant was incubated with buffer-equilibrated Ni-NTA beads (Qiagen) for 4h at 25°C under mild mixing conditions. Then, the Ni-NTA beads were collected and washed with 20 mM imidazole in HBS (pH 7.2). The bound protein was eluted using 200 mM imidazole in HBS (pH 7.2). The TCRαβ heterodimer was further purified by a size-exclusion column (Superdex 200 Increase 10/300 GL (Cytiva)) using an AKTA pure 25 L1 system (Cytiva) equilibrated with HBS (pH 7.2). The eluted fractions were analyzed for purity using SDS-PAGE and subsequently pooled.

MHC-I protein purification and peptide exchange

In order to generate HLA-A*02:01 (MHC-I) monomers, the corresponding α-chain and β2m protein constructs were overexpressed separately in *E.coli*. The protein was refolded from the inclusion bodies in the presence of a UV-cleavable peptide and biotinylated for downstream applications as described previously (*63*). After purification, the protein was concentrated and stored with 20% glycerol at −80°C. For each epitope specificity tested in this study, peptide exchange reactions were set up in a volume of 100 μl containing 0.2 mM peptide and 100 μg/ml HLA-A*02:01 protein in PBS (pH 7.4). The reaction mixture was exposed to 365nm UV-light irradiation for 20 min using a Stratagene UV Stratalinker 2400 in 96-well U-shaped-bottom microplates (Corning). The plate was then transferred to 4°C overnight to complete the exchange. The protein was subsequently buffer exchanged against PBS (pH 7.4) using Microcon centrifugal filters (10 kDa cut-off, MilliporeSigma) to remove the excess free peptide and subsequently spun at 13000×g for 15 min at 4°C to remove aggregates. The protein was filtered and stored at 4°C until further use.

Purification of MHC-II heterodimers and peptide exchange

The ectodomains of HLA-DRA, HLA-DRB1*04:01 and HLA-DRB1*15:01 were cloned into a CMV/R promoter-based vector by Gibson assembly (New England Biolabs). The gene constructs were codon-optimized for mammalian expression. The sequence confirmed (Elim Biopharm) plasmids were amplified in *E.coli*. Plasmids encoding the MHCα and MHCβ-chains of a given MHCαβ hetero-dimer were co-transfected into Expi293F cells (Thermo Fisher Scientific) following the manufacturer recommendations. The transfected cells were enhanced ~18-20h post-transfection with the ExpiFectamine 293 transfection enhancers 1 and 2 (Thermo Fisher Scientific). The supernatant was harvested 5 days post-transfection and incubated with buffer-equilibrated Ni-NTA beads (Qiagen) for 5h at 4°C. The Ni-NTA beads were then collected, washed (20 mM imidazole in HBS (pH 7.2)) and the bound protein was eluted under gravity flow with 200 mM imidazole in HBS (pH 7.2). The protein was buffer-exchanged to remove the imidazole and biotinylated using the BirA biotin-protein ligase reaction kit (Avidity) as per the manufacturer recommendations. The MHC-II heterodimer was subsequently purified by via size-exclusion chromatography (Superdex 200 Increase 10/300 GL (Cytiva)) using an AKTA pure 25 L1 system (Cytiva) equilibrated with HBS (pH 7.2). The eluted fractions were analyzed for purity and pooled, and also assessed for biotinylation efficiency using SDS-PAGE. Thrombin (Novagen) was used to cleave the invariant CLIP peptide from the purified MHC-II molecules to enable exchange with the test peptide. After 2h incubation of MHC-II molecules with thrombin at room temperature, the reaction was stopped by the addition of a protease inhibitor cocktail (MilliporeSigma). The cleaved MHC-II protein was incubated at 30°C overnight in an aqueous solution of 1% octyl β-D-glucopyranoside, 0.1 M NaCl, 50 mM citrate (pH 5.2), 1 mM EDTA, and 0.4 mg/mL test peptide for completion of exchange. Next day, the reaction was neutralized with 1M Tris-HCl (pH 8). The excess peptide was removed during buffer exchange against PBS (pH 7.4) using Microcon centrifugal filters (10 kDa cut-off, MilliporeSigma). The protein was further spun at 13000×g for 15 min at 4°C to remove aggregates and filtered before storing at 4°C until further use.

Generation of pMHC multimer reagents

Here, we generated different multivalent formulations of a given pMHC specificity to enable comparative analysis. In order to ascribe the observed differences to the multimerization scaffold, all the multivalent pMHC formulations (tetramer, dextramer and spheromer) were made using the same stock of purified MHC molecules. The pMHC-tetramers were generated as described previously (*63*). Briefly,

fluorophore-conjugated streptavidin (Invitrogen) was added to each pMHC monomer incrementally to achieve a 4:1 (pMHC:SAv) molar ratio. Next, streptavidin agarose was added to each tetramer for quenching any unbound, biotinylated pMHC. After filtration, biotinylated agarose beads were added to remove any unsaturated streptavidin molecules. The protein was filtered and stored at 4°C until further use. We also used a previously described protocol for generating the pMHC-dextramers (*19*). The biotinylated pMHC molecules were incubated with fluorophore-conjugated streptavidin (Invitrogen) at a molar ratio of ~3.5:1 (pMHC:SAv) for 30 min at room temperature. To this mixture, biotin-dextran (MW = 70 kDa, Thermo Fisher Scientific) was added at a molar ratio of ~30:1 (pMHC:Dextran) and incubated further for another 30 min at room temperature. The spheromer assembly has already been described above.

Binding affinity measurements using biolayer interferometry (BLI)

Binding affinity for the cognate TCR-pMHC pairs was determined by BLI using an Octet QK instrument (ForteBio). The purified, soluble TCRs were captured onto amine reactive second-generation (AR2G) biosensors using the amine reactive second-generation reagent kit. The ligand-bound biosensors were then dipped into a decreasing concentration series (50 μM followed by 2-fold dilutions) of the indicated analytes in PBST (PBS with 0.05% Tween-20) to determine the binding kinetics. A series of unliganded biosensors dipped into the analytes served as controls for referencing. In addition, signals from analyte binding to an irrelevant TCR was used for non-specific binding correction. The traces were processed using ForteBio Data Analysis Software.

Lentiviral transduction for generating T cell lines

The T cell lines were generated as described previously (*34*). Briefly, gene blocks (Integrated DNA Technologies) corresponding to the TCRα and TCRβ chains of a given TCRαβ pair were cloned into the EF1a-MCS-GFP-PGK-puro lentiviral vector. Each sequence confirmed (Elim Biopharm) lentiviral plasmid was separately co-transfected with the gag-pol and VSV-G envelope plasmids into Lenti-X 293T cells (Takara Bio) cultured in DMEM media (Thermo Fisher Scientific) supplemented with 10% FBS (R&D Systems) and 100U/ml of penicillin-streptomycin using FuGENE (Promega) transfection reagent. After 72h, lentiviruses for both TCRα and TCRβ constructs were harvested by collecting the culture supernatant. TCR-deficient Jurkat cells ($\alpha^-\beta^-$) (ATCC) were transduced with the viral supernatant. TCR and CD3 expression was assessed by flow cytometry after staining the cells with anti-TCR α/β (PE, clone 3C10, BioLegend) and anti-CD3 (BV421, clone OKT3, BioLegend) antibodies for 30 min on ice. The cells were washed, resuspended in FACS buffer (PBS with 1% BSA and 2 mM EDTA) and acquired on a BD LSRII flow cytometer. The data was analyzed using FlowJo (v10) software. If TCR expression after lentiviral transduction was <80%, enrichment for TCR expression was performed using anti-TCR α/β (APC, clone 3C10, BioLegend) antibody in conjunction with anti-APC microbeads (Miltenyi Biotec).

Binding of T cell lines with pMHC multimers

The binding of pMHC to T cell lines was monitored by flow cytometry. pMHC multimers with Alexa 647 conjugated streptavidin (Invitrogen) were generated as described above. Binding curves (MFI) were determined using a concentration series of the pMHC multimer reagents. The cells were stained with pMHC multimers (tetramer, dextramer and spheromer) for 1h in FACS buffer. The pMHC multimer staining was done at 4°C or 25°C for MHC-I- and MHC-II-restricted T cell specificities, respectively. The cells were washed and subsequently stained with anti-CD3 (BV421, clone OKT3, BioLegend) antibody for 20 min on ice. The cells were then washed twice, resuspended in FACS buffer and acquired on a Attune NxT Flow Cytometer (Thermo Fisher Scientific). The data was analyzed using FlowJo (v10) software.

Human biological sample collection

Peripheral blood mononuclear cells (PBMCs) from healthy donors were obtained from the Stanford Blood Center according to our IRB approved protocol. All healthy donor samples used in the current study were confirmed to be HLA-A*02:01⁺ and were collected between April 2018 – Feb 2019 before the SARS-CoV-2 pandemic. The EBV and HCMV infection status for these donors was also determined by the Stanford Blood Center.

The COVID-19 patient sample collection for this study was conducted at the Stanford Occupational Health under an IRB approved protocol (Protocol Director, Nadeau). We obtained samples from all COVID-19+ adults who had a positive-test result for the SARS-CoV-2 virus from analysis of nasopharyngeal swab specimens obtained at any point from March 2020 - June 2020. Stanford Health Care clinical laboratory developed internal testing capability with a reverse-transcriptase based polymerase-chain-reaction assay. All participants consented prior to enrolling in the study. We obtained clinical data from Stanford clinical data electronic medical record system as per consented participant permission. This database contains all the clinical data available on all inpatient and outpatient visits to Stanford facilities. The data obtained included patients' demographic details, vital signs, laboratory test results, medication administration data, historical and current medication lists, historical and current diagnoses, clinical notes, and radiological results. Participants were excluded if they were taking any experimental medications (i.e., those medications not approved by a regulatory agency for use in COVID-19). The severity of COVID-19 illness was defined based on the symptom score described by Chen et. al. (*64*).

PBMC staining and flow cytometry

PBMCs were thawed in a water bath set at 37°C and the cells were immediately transferred to warm RPMI media (Thermo Fisher Scientific) supplemented with 10% FBS (R&D Systems) and 100U/ml of penicillin-streptomycin. After washing, the cells were filtered (70 μm cell strainer) and rested for 1h at 37°C. $CD8^+$ T cells were enriched from PBMCs by negative selection using a FITC-conjugated antibody cocktail against non-$CD8^+$ T cells (anti-CD14 (Clone HCD14, BioLegend), anti-CD19 (Clone HIB19, BioLegend), anti-CD33 (Clone HIM3-4, BioLegend) and anti-γδ TCR (Clone 5A6.E9, ThermoFisher Scientific)) followed by magnetic bead depletion using anti-FITC microbeads (Miltenyi Biotec). The enriched $CD8^+$ T cells were washed and resuspended in FACS buffer for staining. All pMHC-multimer staining was done for 1h at 4°C after incubating the cells with Human TruStain FcX (BioLegend) for 15 min. In order to compare the frequency of viral (influenza and HCMV) antigen-specific T cells detected using tetramer or spheromer, each sample was divided equally after $CD8^+$ T cell enrichment and stained with M1-A*02:01 (Alexa 647) and pp65-A*02:01 (PE) formulated as tetramer or spheromer. The pMHC-multimer formulations were used at a monomeric concentration of 100nM. The gag-A*02:01 (Alexa 488) pMHC-multimer (200nM) was used as an irrelevant specificity control. The cells were subsequently stained with anti-CD19 (BV510, clone HIB19), anti-γδ TCR (BV510, clone B1), anti-CD33 (BV510, clone HIM3-4), anti-CD3 (PE/Cyanine7, clone OKT3), anti-CD8 (BUV396, clone RPA-T8, BD Biosciences), anti-CD4 (BV785, clone RPA-T4), anti-CCR7 (PE/Dazzle 594, clone G043H7), anti-CD45RA (BV711, clone HI100) and an amine-reactive viability stain (Live/dead fixable aqua dead cell stain kit; Invitrogen) for 30 min on ice, washed, resuspended in FACS buffer and acquired on a BD LSRII flow cytometer. All the antibodies for flow cytometry were purchased from BioLegend unless mentioned otherwise. The data was analyzed using FlowJo (v10) software.

For the simultaneous detection of multiple SARS-CoV-2 epitopes (described below) using the spheromer technology, we adapted a combinatorial staining approach developed previously (*49*). Briefly, each peptide was assigned a unique fluorophore-barcode that allows the simultaneous detection of 2^n-1 specificities in a sample, where n is the number of distinct fluorophore labels. The relative concentrations for pMHC monomers associated with each fluorophore label (Alexa 647, eFluor 450, PE and PE/Cyanine7) was experimentally determined. Four T cell lines with distinct antigen specificities (M1-A*02:01, pp65-A*02:01, BMLF1-A*02:01 and BHW58-A*02:01) were mixed at a pre-determined ratio with TCR-deficient Jurkat cells ($\alpha^-\beta^-$) and stained with a pool of spheromers, wherein each cognate pMHC was associated with a unique fluorescent tag. The cells were further labeled with anti-CD3 (FITC, clone OKT3, BioLegend) for 30 min, washed, resuspended in flow cytometry buffer and acquired on a BD LSRII flow cytometer. The data was analyzed to determine the optimal concentration for pMHC monomers associated with each fluorophore label (Alexa 647; 100 nM, eFluor 450; 125 nM, PE; 75 nM and PE/Cyanine7; 50 nM) that provided the maximum separation between the distinct T cell lines. The gag-A*02:01 pMHC-spheromer defined by the fluorophore-barcode (Alexa 647 + eFluor 450 + PE + PE/Cyanine7) was used as irrelevant specificity control. After staining the PBMC samples with spheromer pools displaying SARS-CoV-2 epitopes, magnetic enrichment of spheromer-positive population was performed using super-paramagnetic beads conjugated to an anti-c-myc monoclonal antibody (Miltenyi Biotec). The α-chain of HLA-A*02:01 is engineered to contain an exposed, C-terminal c-myc tag. The cells were subsequently stained with anti-CD19 (BV510, clone HIB19), anti-γδ TCR (BV510, clone B1), anti-CD33 (BV510, clone HIM3-4), anti-CD3 (FITC, clone OKT3), anti-CD8 (BUV396, clone RPA-T8, BD Biosciences), anti-CD4 (BV785, clone RPA-T4), anti-CCR7 (PE/Dazzle 594, clone G043H7), anti-CD45RA (BV711, clone HI100) and an amine-reactive viability stain (Live/dead fixable aqua dead cell stain kit; Invitrogen) for 30 min. The antigen-specific T cell enumerated as described previously (*29*, *39*). Briefly, the frequency was calculated based on the total number of pMHC $multimer^+$ cells divided by the total $CD8^+$ T cells. The absolute counts of the desired cell populations were determined using BD Trucount beads as per the manufacturer's recommendation (BD Biosciences) by measuring the number of bead events in $1/10^{th}$ of the initial staining reaction (pre-enriched) and the eluted fraction after magnetic enrichment. The % recovery after enrichment is estimated by bead count in the eluted fraction. In experiments wherein magnetic enrichment of the pMHC multimer stained cells was not performed, the entire sample was recorded, and the total cell count of the desired populations determined using BD Trucount beads (BD Biosciences) was used for calculating the frequency of antigen-specific T cells. The sensitivity of pMHC multimer staining after magnetic enrichment was determined by comparing the expected versus the actual numbers of TCR1 cells (BHW58-A*02:01 specificity) recovered from a serial dilution of TCR1 cells into TCR-deficient Jurkat cells ($\alpha^-\beta^-$). The sensitivity of multimer staining was also determined independently by calculating the recovery of TCR1 cells spiked into PBMCs from a healthy HLA-A*02:01 donor. The TCR1 cells were labeled with a viability dye before spiking them into a PBMC sample. The limit of detection after magnetic enrichment was determined to be ~2´10^{-7} (i.e., one antigen-specific T cell in several million total $CD8^+$ T cells). ~0.1´10^6 cells from each COVID-19 patient sample was also separately stained (without spheromer pools) with anti-CD19 (BV510, clone HIB19), anti-γδ TCR (BV510, clone B1),

anti-CD33 (BV510, clone HIM3-4), anti-CD3 (FITC, clone OKT3), anti-CD8 (BUV396, clone RPA-T8, BD Biosciences), anti-CD4 (BV785, clone RPA-T4), anti-CCR7 (PE/Dazzle 594, clone G043H7), anti-CD45RA (BV711, clone HI100), anti-HLA-A2 (Alexa 700, clone BB7.2) antibody and an amine-reactive viability stain (Live/dead fixable aqua dead cell stain kit; Invitrogen) for 30 min on ice. All the antibodies for flow cytometry were purchased from BioLegend unless mentioned otherwise. The cells were washed, resuspended in FACS buffer and processed using a BD LSRII flow cytometer. The data was analyzed using FlowJo (v10) software.

Selection of SARS-CoV-2 peptides and sequence conservation analysis

The complete genome sequence for SARS-CoV-2 isolate SARS-CoV-2/USA/WA-CDC-WA1/2020 (GenBank accession ID: MN985325) was obtained from the NCBI database. The binding of all possible 9-mers from SARS-CoV-2 ORF1ab, S, M and N proteins to HLA-A*02:01 was predicted following the immune epitope database and analysis resource (IEDB) recommendations (http://tools.iedb.org/mhci/) (*44*). The peptide binding predictions were cross validated using the SYFPEITHI algorithms (*45*). We further prioritized peptides based on the biochemical properties of amino acids at positions P2, P5 and P9 (*40*, *46*). The binding of selected peptides to HLA-A*02:01 was further experimentally validated by an MHC stabilization assay using the transporter associated with antigen processing (TAP) deficient T2 cell line (ATCC) expressing HLA-A*02:01. Briefly, T2 cells were incubated with a concentration series of the test peptide (GenScript) in AIM V serum free media (Thermo Fisher Scientific) for 1h at 37°C. The cells were then transferred to a lower temperature (26°C) for another 14h, before returning them to 37°C for 3h prior to antibody staining. The cells were washed free of any unbound peptide and incubated with anti-HLA-A2 (PE, clone BB7.2) antibody and an amine-reactive viability stain (Live/dead fixable aqua dead cell stain kit; Invitrogen) for 30 min on ice. Subsequently, cells were washed, resuspended in FACS buffer and acquired on a BD LSRII flow cytometer. T2 cells incubated in AIM V serum free media alone (no peptide) served as a negative control. The list of SARS-CoV-2 peptides evaluated using the spheromer technology in this study are listed in Table S1.

To perform a sequence conservation analysis of the peptides selected from SARS-CoV-2 across other seasonal hCoVs, we obtained representative whole genome sequences for 229E (HCoV_229E/Seattle/USA/SC0865/2019, GenBank accession ID: MN306046), HKU1 (HCoV_HKU1/SC2628/2017, GenBank accession ID: KY983584), NL63 (HCoV_NL63/UF-2/2015, GenBank accession ID: KX179500) and OC43 (HCoV_OC43/Seattle/USA/SC9430/2018, GenBank accession ID: MN306053) from the NCBI database. The binding of all possible 9-mers from ORF1ab, S, M and N proteins to HLA-A*02:01 for each of the seasonal hCoV reference strains listed above was predicted following the immune epitope database and analysis resource (IEDB) recommendations (http://tools.iedb.org/mhci/). We then filtered the peptides based on percentile rank (<5.0). A lower percentile rank indicates higher affinity. This was done to restrict the search for cross-reactive peptides in hCoVs that are potentially functional owing to their ability to bind HLA-A*02:01, a pre-requisite to activate T cells. We then calculated the pairwise sequence similarity score for each of the selected SARS-CoV-2 peptides against all filtered seasonal hCoV peptides using the sequence manipulation suite (*65*). The sequence similarity score was calculated allowing for amino acid substitutions (GA, VLI, FYW, ST, KR, DE and NQ) with similar biochemical properties (*47*, *48*). The list of seasonal hCoV peptides identified based on the similarity score is given in Table S1. The sequence similarity (%) and the percentile rank are also mentioned. The sequences of the SARS-CoV-2 variants of concern for conservation analysis were obtained from the GISAID database.

Single-cell paired αβ-TCR sequencing

Multiplexed αβ-TCR sequencing was done following previously established protocols (*33*). In brief, single spheromer$^+$ CD8$^+$ T cells (for influenza-M1, HCMV-pp65 and SARS-CoV-2 specificities) were sorted into 96-well plates containing 12 μl OneStep RT-PCR buffer (Qiagen). Reverse transcription was done using the OneStep RT-PCR kit (Qiagen) and the resulting cDNA was used for TCRα and TCRβ amplification using multiplex primers. DNA barcodes were also incorporated within the amplified sequences before processing the samples in a single MiSeq2 ´ 300bp sequencing run. The paired sequencing reads were joined, demultiplexed, and mapped to the human TCR reference dataset available at the international ImMunoGeneTics information system (IMGT) as reported previously (*33*).

Identification of TCR 'motifs' with shared antigen specificity using GLIPH2

We benchmarked the TCR repertoire of antigen-specific (influenza-M1 and HCMV-pp65) CD8$^+$ T cells detected using the spheromer by comparing them to tetramer or dextramer derived sequences retrieved from the VDJdb database (*28*). For each antigen specificity, we implemented the GLIPH2 algorithm to quantify the number of clusters (characterized by a distinct TCR CDR3β motif) that were unique to the spheromer or had an overlap with TCR sequences reported using the tetramer or dextramer. Briefly, the GLIPH2 algorithm compared the antigen-specific TCRs (input dataset) against a reference dataset of 273,920 distinct TCR CDR3β sequences from 12 healthy individuals to generate clusters with unique TCR CDR3β motifs that are significantly enriched (p-value ≤ 0.05, Fisher's exact test) in the input dataset as previously described (*34*).

We also analyzed the SARS-CoV-2 epitope-specific TCR sequences identified from unexposed, healthy individuals using the spheromer by implementing the GLIPH2 algorithm. The TCR sequences from COVID-19 patient samples for this analysis were obtained from a published dataset (*66*). The inclusion of multiple statistical measurements in the GLIPH2 output accounting for Vβ gene usage biases, CDR3β length distribution (relevant only for local motifs), cluster size, HLA allele usage, and clonal expansion facilitates the calling of high-confidence specificity groups.

In vitro stimulation of T cell lines

The stimulation assay was done as previously described (*62*). The assay was setup in 96-well clear round bottom microplates (Corning) with a volume of 200μl during all incubation steps. T2 cells expressing HLA-A*02:01 were plated at a density of 50,000 cells/well in IMDM media (Thermo Fisher Scientific) supplemented with 10% FBS (R&D Systems) and 100U/ml of penicillin-streptomycin and pulsed with 100 mM of the test peptide for 3h at 37°C. The cells were then washed and co-cultured with Jurkat cells expressing an exogenous TCR of interest (100,000 cells/well) in RPMI media (Thermo Fisher Scientific) supplemented with 10% FBS (R&D Systems) and 100U/ml of penicillin-streptomycin for 16h. Next day, the cells were washed with FACS buffer and stained with anti-CD3 (APC, clone OKT3) and anti-CD69 (PE, clone FN50) antibodies for 20 min at 4°C. Cells were washed, resuspended in FACS buffer and analyzed on an Attune NxT Flow Cytometer (Thermo Fisher Scientific). The data was analyzed using FlowJo (v10) software.

Statistical analysis

R statistical package was used to perform the Fisher's exact test to compute TRBV gene enrichment across different pMHC formulations using the fisher.test function. Fisher's exact test was also used to determine the significance levels of the distribution of GLIPH2 TCR motifs at different WHO scores identified using peptides either unique to SARS-CoV-2 or conserved across human coronaviruses. Next, we performed a meta-analysis to combine the p-values from individual hypothesis tests to assess the significance of the overall distribution. Dimensionality reduction analysis were also performed in R. UMAP to visualize multiparametric flow cytometry data was generated using the "umap" package. Additional data and statistical analyses were done in GraphPad Prism. The statistical details for each experiment are provided in the associated figure legends.

SUPPLEMENTARY MATERIALS

immunology.sciencemag.org/cgi/content/full/6/61/eabg5669/DC1

Figure S1. Selection and characterization of scaffold candidates.
Figure S2. Optimization of molecular tethers on the maxi-ferritin scaffold for SAv mediated conjugation of pMHC molecules.
Figure S3. Spheromer assembly is not perturbed by the inclusion of differently sized fluorophores.
Figure S4. Titration of semi-saturated SAv-$pMHC_2$ with the functionalized scaffold.
Figure S5. pMHC-TCR binding affinity measurements by biolayer interferometry.
Figure S6. Staining of T cell lines with pMHC multimers.
Figure S7. Spheromers stain better than tetramer irrespective of the conjugated fluorophore.
Figure S8. Gating strategy and representative flow cytometry dot plots comparing tetramer and spheromer staining on the same sample.
Figure S9. Comparison of pMHC multimer staining using reagents generated in-house or procured from the NIH tetramer core facility.
Figure S10. Validation of the unique antigen-specific TCR motifs identified using spheromer.
Figure S11. Experimental validation of the predicted SARS-CoV-2 peptide binding to HLA-A*02:01.
Figure S12. Combinatorial staining with spheromer pools to resolve multiple antigen specificities simultaneously was adapted from a previously described approach.
Table S1. Summary statistics of the study cohorts.
Table S2. Demographic and clinical information for COVID-19 patients.
Table S3. SARS-CoV-2 peptide panel.
Data file S1. Raw data file (Excel spreadsheet).
MDAR Checklist

REFERENCES AND NOTES

1. C. Huang, Y. Wang, X. Li, L. Ren, J. Zhao, Y. Hu, L. Zhang, G. Fan, J. Xu, X. Gu, Z. Cheng, T. Yu, J. Xia, Y. Wei, W. Wu, X. Xie, W. Yin, H. Li, M. Liu, Y. Xiao, H. Gao, L. Guo, J. Xie, G. Wang, R. Jiang, Z. Gao, Q. Jin, J. Wang, B. Cao, Clinical features of patients infected with 2019 novel coronavirus in Wuhan, China. *Lancet* **395**, 497–506 (2020). doi:10.1016/S0140-6736(20)30183-5 Medline
2. F. Krammer, SARS-CoV-2 vaccines in development. *Nature* **586**, 516–527 (2020). doi:10.1038/s41586-020-2798-3 Medline
3. K. H. D. Crawford, A. S. Dingens, R. Eguia, C. R. Wolf, N. Wilcox, J. K. Logue, K. Shuey, A. M. Casto, B. Fiala, S. Wrenn, D. Pettie, N. P. King, A. L. Greninger, H. Y. Chu, J. D. Bloom, Dynamics of neutralizing antibody titers in the months after SARS-CoV-2 infection. *J. Infect. Dis.* jiaa618 (2020). Medline
4. M. Hellerstein, What are the roles of antibodies versus a durable, high quality T-cell response in protective immunity against SARS-CoV-2? *Vaccine X* **6**, 100076 (2020). doi:10.1016/j.jvacx.2020.100076 Medline
5. J. Seow *et al.*, Longitudinal evaluation and decline of antibody responses in SARS-CoV-2 infection. *medRxiv*, 2020.2007.2009.20148429 (2020).
6. A. Wajnberg, F. Amanat, A. Firpo, D. R. Altman, M. J. Bailey, M. Mansour, M. McMahon, P. Meade, D. R. Mendu, K. Muellers, D. Stadlbauer, K. Stone, S. Strohmeier, V. Simon, J. Aberg, D. L. Reich, F. Krammer, C. Cordon-Cardo, Robust neutralizing antibodies to SARS-CoV-2 infection persist for months. *Science* **370**, 1227–1230 (2020). doi:10.1126/science.abd7728 Medline
7. A. P. Ferretti, T. Kula, Y. Wang, D. M. V. Nguyen, A. Weinheimer, G. S. Dunlap, Q. Xu, N. Nabilsi, C. R. Perullo, A. W. Cristofaro, H. J. Whitton, A. Virbasius, K. J. Olivier Jr., L. R. Buckner, A. T. Alistar, E. D. Whitman, S. A. Bertino, S. Chattopadhyay, G. MacBeath, Unbiased Screens Show $CD8^+$ T Cells of COVID-19 Patients Recognize Shared Epitopes in SARS-CoV-2 that Largely Reside outside the Spike Protein. *Immunity* **53**, 1095–1107.e3 (2020). doi:10.1016/j.immuni.2020.10.006 Medline
8. A. Grifoni, D. Weiskopf, S. I. Ramirez, J. Mateus, J. M. Dan, C. R. Moderbacher, S. A. Rawlings, A. Sutherland, L. Premkumar, R. S. Jadi, D. Marrama, A. M. de Silva, A. Frazier, A. F. Carlin, J. A. Greenbaum, B. Peters, F. Krammer, D. M. Smith, S. Crotty, A. Sette, Targets of T Cell Responses to SARS-CoV-2 Coronavirus in Humans with COVID-19 Disease and Unexposed Individuals. *Cell* **181**, 1489–1501.e15 (2020). doi:10.1016/j.cell.2020.05.015 Medline
9. N. Le Bert, A. T. Tan, K. Kunasegaran, C. Y. L. Tham, M. Hafezi, A. Chia, M. H. Y. Chng, M. Lin, N. Tan, M. Linster, W. N. Chia, M. I.-C. Chen, L.-F. Wang, E. E. Ooi, S. Kalimuddin, P. A. Tambyah, J. G.-H. Low, Y.-J. Tan, A. Bertoletti, SARS-CoV-2-specific T cell immunity in cases of COVID-19 and SARS, and uninfected controls. *Nature* **584**, 457–462 (2020). doi:10.1038/s41586-020-2550-z Medline
10. J. Mateus, A. Grifoni, A. Tarke, J. Sidney, S. I. Ramirez, J. M. Dan, Z. C. Burger, S. A. Rawlings, D. M. Smith, E. Phillips, S. Mallal, M. Lammers, P. Rubiro, L. Quiambao, A. Sutherland, E. D. Yu, R. da Silva Antunes, J. Greenbaum, A. Frazier, A. J. Markmann, L. Premkumar, A. de Silva, B. Peters, S. Crotty, A. Sette, D. Weiskopf, Selective and cross-reactive SARS-CoV-2 T cell epitopes in unexposed humans. *Science* **370**, 89–94 (2020). doi:10.1126/science.abd3871 Medline
11. A. Nelde, T. Bilich, J. S. Heitmann, Y. Maringer, H. R. Salih, M. Roerden, M. Lübke, J.

Bauer, J. Rieth, M. Wacker, A. Peter, S. Hörber, B. Traenkle, P. D. Kaiser, U. Rothbauer, M. Becker, D. Junker, G. Krause, M. Strengert, N. Schneiderhan-Marra, M. F. Templin, T. O. Joos, D. J. Kowalewski, V. Stos-Zweifel, M. Fehr, A. Rabsteyn, V. Mirakaj, J. Karbach, E. Jäger, M. Graf, L.-C. Gruber, D. Rachfalski, B. Preuß, I. Hagelstein, M. Märklin, T. Bakchoul, C. Gouttefangeas, O. Kohlbacher, R. Klein, S. Stevanović, H.-G. Rammensee, J. S. Walz, SARS-CoV-2-derived peptides define heterologous and COVID-19-induced T cell recognition. *Nat. Immunol.* **22**, 74–85 (2021). doi:10.1038/s41590-020-00808-x Medline

12. Y. Peng, A. J. Mentzer, G. Liu, X. Yao, Z. Yin, D. Dong, W. Dejnirattisai, T. Rostron, P. Supasa, C. Liu, C. Lopez-Camacho, J. Slon-Campos, Y. Zhao, D. Stuart, G. Paeson, J. Grimes, F. Antson, O. W. Bayfield, D. E. Hawkins, D. S. Ker, L. Turtle, K. Subramaniam, P. Thomson, P. Zhang, C. Dold, J. Ratcliff, P. Simmonds, T. de Silva, P. Sopp, D. Wellington, U. Rajapaksa, Y. L. Chen, M. Salio, G. Napolitani, W. Paes, P. Borrow, B. Kessler, J. W. Fry, N. F. Schwabe, M. G. Semple, K. J. Baillie, S. Moore, P. J. Openshaw, A. Ansari, S. Dunachie, E. Barnes, J. Frater, G. Kerr, P. Goulder, T. Lockett, R. Levin, R. J. Cornall, C. Conlon, P. Klenerman, A. McMichael, G. Screaton, J. Mongkolsapaya, J. C. Knight, G. Ogg, T. Dong, Broad and strong memory CD4 $^{+}$ and CD8 $^{+}$ T cells induced by SARS-CoV-2 in UK convalescent COVID-19 patients. *bioRxiv* 2020.06.05.134551 (2020). Medline
13. I. Schulien, J. Kemming, V. Oberhardt, K. Wild, L. M. Seidel, S. Killmer, F. Sagar, F. Daul, M. Salvat Lago, A. Decker, H. Luxenburger, B. Binder, D. Bettinger, O. Sogukpinar, S. Rieg, M. Panning, D. Huzly, M. Schwemmle, G. Kochs, C. F. Waller, A. Nieters, D. Duerschmied, F. Emmerich, H. E. Mei, A. R. Schulz, S. Llewellyn-Lacey, D. A. Price, T. Boettler, B. Bengsch, R. Thimme, M. Hofmann, C. Neumann-Haefelin, Characterization of pre-existing and induced SARS-CoV-2-specific CD8^{+} T cells. *Nat. Med.* **27**, 78–85 (2021). doi:10.1038/s41591-020-01143-2 Medline
14. L. C. Wu, D. S. Tuot, D. S. Lyons, K. C. Garcia, M. M. Davis, Two-step binding mechanism for T-cell receptor recognition of peptide MHC. *Nature* **418**, 552–556 (2002). doi:10.1038/nature00920 Medline
15. M. M. Davis, J. J. Boniface, Z. Reich, D. Lyons, J. Hampl, B. Arden, Y. Chien, Ligand recognition by alpha beta T cell receptors. *Annu. Rev. Immunol.* **16**, 523–544 (1998). doi:10.1146/annurev.immunol.16.1.523 Medline
16. J. D. Altman, P. A. H. Moss, P. J. R. Goulder, D. H. Barouch, M. G. McHeyzer-Williams, J. I. Bell, A. J. McMichael, M. M. Davis, Phenotypic analysis of antigen-specific T lymphocytes. *Science* **274**, 94–96 (1996). doi:10.1126/science.274.5284.94 Medline
17. P. Batard, D. A. Peterson, E. Devêvre, P. Guillaume, J.-C. Cerottini, D. Rimoldi, D. E. Speiser, L. Winther, P. Romero, Dextramers: New generation of fluorescent MHC class I/peptide multimers for visualization of antigen-specific CD8+ T cells. *J. Immunol. Methods* **310**, 136–148 (2006). doi:10.1016/j.jim.2006.01.006 Medline
18. G. Dolton, K. Tungatt, A. Lloyd, V. Bianchi, S. M. Theaker, A. Trimby, C. J. Holland, M. Donia, A. J. Godkin, D. K. Cole, P. T. Straten, M. Peakman, I. M. Svane, A. K. Sewell, More tricks with tetramers: A practical guide to staining T cells with peptide-MHC multimers. *Immunology* **146**, 11–22 (2015). doi:10.1111/imm.12499 Medline
19. J. Huang, X. Zeng, N. Sigal, P. J. Lund, L. F. Su, H. Huang, Y. H. Chien, M. M. Davis, Detection, phenotyping, and quantification of antigen-specific T cells using a peptide-MHC dodecamer. *Proc. Natl. Acad. Sci. U.S.A.* **113**, E1890–E1897 (2016). doi:10.1073/pnas.1602488113 Medline
20. G. Dolton, A. Lissina, A. Skowera, K. Ladell, K. Tungatt, E. Jones, D. Kronenberg-Versteeg, H. Akpovwa, J. M. Pentier, C. J. Holland, A. J. Godkin, D. K. Cole, M. A. Neller, J. J. Miles, D. A. Price, M. Peakman, A. K. Sewell, Comparison of peptide-major histocompatibility complex tetramers and dextramers for the identification of antigen-specific T cells. *Clin. Exp. Immunol.* **177**, 47–63 (2014). doi:10.1111/cei.12339 Medline
21. C. Rius, M. Attaf, K. Tungatt, V. Bianchi, M. Legut, A. Bovay, M. Donia, P. Thor Straten, M. Peakman, I. M. Svane, S. Ott, T. Connor, B. Szomolay, G. Dolton, A. K. Sewell, Peptide-MHC Class I Tetramers Can Fail To Detect Relevant Functional T Cell Clonotypes and Underestimate Antigen-Reactive T Cell Populations. *J. Immunol.* **200**, 2263–2279 (2018). doi:10.4049/jimmunol.1700242 Medline
22. R. E. Chen, X. Zhang, J. B. Case, E. S. Winkler, Y. Liu, L. A. VanBlargan, J. Liu, J. M. Errico, X. Xie, N. Suryadevara, P. Gilchuk, S. J. Zost, S. Tahan, L. Droit, J. S. Turner, W. Kim, A. J. Schmitz, M. Thapa, D. Wang, A. C. M. Boon, R. M. Presti, J. A. O'Halloran, A. H. J. Kim, P. Deepak, D. Pinto, D. H. Fremont, J. E. Crowe Jr., D. Corti, H. W. Virgin, A. H. Ellebedy, P.-Y. Shi, M. S. Diamond, Resistance of SARS-CoV-2 variants to neutralization by monoclonal and serum-derived polyclonal antibodies. *Nat. Med.* **27**, 717–726 (2021). doi:10.1038/s41591-021-01294-w Medline
23. L. E. Bevers, E. C. Theil, Maxi- and mini-ferritins: Minerals and protein nanocages. *Prog. Mol. Subcell. Biol.* **52**, 29–47 (2011). doi:10.1007/978-3-642-21230-7_2 Medline
24. G. Ueda, A. Antanasijevic, J. A. Fallas, W. Sheffler, J. Copps, D. Ellis, G. B. Hutchinson, A. Moyer, A. Yasmeen, Y. Tsybovsky, Y.-J. Park, M. J. Bick, B. Sankaran, R. A. Gillespie, P. J. M. Brouwer, P. H. Zwart, D. Veesler, M. Kanekiyo, B. S. Graham, R. W. Sanders, J. P. Moore, P. J. Klasse, A. B. Ward, N. P. King, D. Baker, Tailored design of protein nanoparticle scaffolds for multivalent presentation of viral glycoprotein antigens. *eLife* **9**, e57659 (2020). doi:10.7554/eLife.57659 Medline
25. A. Razvi, J. M. Scholtz, Lessons in stability from thermophilic proteins. *Protein Sci.* **15**, 1569–1578 (2006). doi:10.1110/ps.062130306 Medline
26. P. J. Schatz, Use of peptide libraries to map the substrate specificity of a peptide-modifying enzyme: A 13 residue consensus peptide specifies biotinylation in Escherichia coli. *Biotechnology (N. Y.)* **11**, 1138–1143 (1993). Medline
27. J. S. Klein, S. Jiang, R. P. Galimidi, J. R. Keeffe, P. J. Bjorkman, Design and characterization of structured protein linkers with differing flexibilities. *Protein Eng. Des. Sel.* **27**, 325–330 (2014). doi:10.1093/protein/gzu043 Medline
28. D. V. Bagaev, R. M. A. Vroomans, J. Samir, U. Stervbo, C. Rius, G. Dolton, A. Greenshields-Watson, M. Attaf, E. S. Egorov, I. V. Zvyagin, N. Babel, D. K. Cole, A. J. Godkin, A. K. Sewell, C. Kesmir, D. M. Chudakov, F. Luciani, M. Shugay, VDJdb in 2019: Database extension, new analysis infrastructure and a T-cell receptor motif compendium. *Nucleic Acids Res.* **48** (D1), D1057–D1062 (2020). doi:10.1093/nar/gkz874 Medline
29. C. Alanio, F. Lemaitre, H. K. Law, M. Hasan, M. L. Albert, Enumeration of human antigen-specific naive CD8+ T cells reveals conserved precursor frequencies. *Blood* **115**, 3718–3725 (2010). doi:10.1182/blood-2009-10-251124 Medline
30. G. M. Gillespie, M. R. Wills, V. Appay, C. O'Callaghan, M. Murphy, N. Smith, P. Sissons, S. Rowland-Jones, J. I. Bell, P. A. H. Moss, Functional heterogeneity and high frequencies of cytomegalovirus-specific CD8(+) T lymphocytes in healthy seropositive donors. *J. Virol.* **74**, 8140–8150 (2000). doi:10.1128/JVI.74.17.8140-8150.2000 Medline
31. E. J. Grant, T. M. Josephs, S. A. Valkenburg, L. Wooldridge, M. Hellard, J. Rossjohn, M. Bharadwaj, K. Kedzierska, S. Gras, Lack of Heterologous Cross-reactivity toward HLA-A*02:01 Restricted Viral Epitopes Is Underpinned by Distinct αβT Cell Receptor Signatures. *J. Biol. Chem.* **291**, 24335–24351 (2016). doi:10.1074/jbc.M116.753988 Medline
32. T. H. Nguyen, A. C. L. Tan, S. D. Xiang, A. Goubier, K. L. Harland, E. B. Clemens, M. Plebanski, K. Kedzierska, Understanding CD8^{+} T-cell responses toward the native and alternate HLA-A*02:01-restricted WT1 epitope. *Clin. Transl. Immunology* **6**, e134 (2017). doi:10.1038/cti.2017.4 Medline
33. A. Han, J. Glanville, L. Hansmann, M. M. Davis, Linking T-cell receptor sequence to functional phenotype at the single-cell level. *Nat. Biotechnol.* **32**, 684–692 (2014). doi:10.1038/nbt.2938 Medline
34. H. Huang, C. Wang, F. Rubelt, T. J. Scriba, M. M. Davis, Analyzing the Mycobacterium tuberculosis immune response by T-cell receptor clustering with GLIPH2 and genome-wide antigen screening. *Nat. Biotechnol.* **38**, 1194–1202 (2020). doi:10.1038/s41587-020-0505-4 Medline
35. W. S. DeWitt 3rd, A. Smith, G. Schoch, J. A. Hansen, F. A. Matsen 4th, P. Bradley, Human T cell receptor occurrence patterns encode immune history, genetic background, and receptor specificity. *eLife* **7**, e38358 (2018). doi:10.7554/eLife.38358 Medline
36. I. Miconnet, A. Marrau, A. Farina, P. Taffé, S. Vigano, A. Harari, G. Pantaleo, Large TCR diversity of virus-specific CD8 T cells provides the mechanistic basis for massive TCR renewal after antigen exposure. *J. Immunol.* **186**, 7039–7049 (2011). doi:10.4049/jimmunol.1003309 Medline
37. G. B. Stewart-Jones, A. J. McMichael, J. I. Bell, D. I. Stuart, E. Y. Jones, A structural basis for immunodominant human T cell receptor recognition. *Nat. Immunol.* **4**, 657–663 (2003). doi:10.1038/ni942 Medline
38. X. Yang, M. Gao, G. Chen, B. G. Pierce, J. Lu, N. P. Weng, R. A. Mariuzza, Structural Basis for Clonal Diversity of the Public T Cell Response to a Dominant Human Cytomegalovirus Epitope. *J. Biol. Chem.* **290**, 29106–29119 (2015).

doi:10.1074/jbc.M115.691311 Medline
39. L. F. Su, B. A. Kidd, A. Han, J. J. Kotzin, M. M. Davis, Virus-specific CD4(+) memory-phenotype T cells are abundant in unexposed adults. *Immunity* **38**, 373–383 (2013). doi:10.1016/j.immuni.2012.10.021 Medline
40. W. Yu, N. Jiang, P. J. R. Ebert, B. A. Kidd, S. Müller, P. J. Lund, J. Juang, K. Adachi, T. Tse, M. E. Birnbaum, E. W. Newell, D. M. Wilson, G. M. Grotenbreg, S. Valitutti, S. R. Quake, M. M. Davis, Clonal Deletion Prunes but Does Not Eliminate Self-Specific αβ CD8(+) T Lymphocytes. *Immunity* **42**, 929–941 (2015). doi:10.1016/j.immuni.2015.05.001 Medline
41. W. W. Kwok, V. Tan, L. Gillette, C. T. Littell, M. A. Soltis, R. B. LaFond, J. Yang, E. A. James, J. H. DeLong, Frequency of epitope-specific naive CD4(+) T cells correlates with immunodominance in the human memory repertoire. *J. Immunol.* **188**, 2537–2544 (2012). doi:10.4049/jimmunol.1102190 Medline
42. J. J. Moon, H. H. Chu, M. Pepper, S. J. McSorley, S. C. Jameson, R. M. Kedl, M. K. Jenkins, Naive CD4(+) T cell frequency varies for different epitopes and predicts repertoire diversity and response magnitude. *Immunity* **27**, 203–213 (2007). doi:10.1016/j.immuni.2007.07.007 Medline
43. J. J. Obar, K. M. Khanna, L. Lefrançois, Endogenous naive CD8+ T cell precursor frequency regulates primary and memory responses to infection. *Immunity* **28**, 859–869 (2008). doi:10.1016/j.immuni.2008.04.010 Medline
44. Y. Kim, J. Ponomarenko, Z. Zhu, D. Tamang, P. Wang, J. Greenbaum, C. Lundegaard, A. Sette, O. Lund, P. E. Bourne, M. Nielsen, B. Peters, Immune epitope database analysis resource. *Nucleic Acids Res.* **40** (W1), W525-30 (2012). doi:10.1093/nar/gks438 Medline
45. H. Rammensee, J. Bachmann, N. P. Emmerich, O. A. Bachor, S. Stevanović, SYFPEITHI: Database for MHC ligands and peptide motifs. *Immunogenetics* **50**, 213–219 (1999). doi:10.1007/s002510050595 Medline
46. K. Falk, O. Rötzschke, S. Stevanović, G. Jung, H. G. Rammensee, Allele-specific motifs revealed by sequencing of self-peptides eluted from MHC molecules. *Nature* **351**, 290–296 (1991). doi:10.1038/351290a0 Medline
47. S. Frankild, R. J. de Boer, O. Lund, M. Nielsen, C. Kesmir, Amino acid similarity accounts for T cell cross-reactivity and for "holes" in the T cell repertoire. *PLOS ONE* **3**, e1831 (2008). doi:10.1371/journal.pone.0001831 Medline
48. G. Petrova, A. Ferrante, J. Gorski, Cross-reactivity of T cells and its role in the immune system. *Crit. Rev. Immunol.* **32**, 349–372 (2012). doi:10.1615/CritRevImmunol.v32.i4.50 Medline
49. E. W. Newell, L. O. Klein, W. Yu, M. M. Davis, Simultaneous detection of many T-cell specificities using combinatorial tetramer staining. *Nat. Methods* **6**, 497–499 (2009). doi:10.1038/nmeth.1344 Medline
50. D. M. Altmann, R. J. Boyton, SARS-CoV-2 T cell immunity: Specificity, function, durability, and role in protection. *Sci. Immunol.* **5**, eabd6160 (2020). doi:10.1126/sciimmunol.abd6160 Medline
51. M. M. Davis, T cell analysis in vaccination. *Curr. Opin. Immunol.* **65**, 70–73 (2020). doi:10.1016/j.coi.2020.05.002 Medline
52. J. J. Melenhorst, P. Scheinberg, P. K. Chattopadhyay, A. Lissina, E. Gostick, D. K. Cole, L. Wooldridge, H. A. van den Berg, E. Bornstein, N. F. Hensel, D. C. Douek, M. Roederer, A. K. Sewell, A. J. Barrett, D. A. Price, Detection of low avidity CD8(+) T cell populations with coreceptor-enhanced peptide-major histocompatibility complex class I tetramers. *J. Immunol. Methods* **338**, 31–39 (2008). doi:10.1016/j.jim.2008.07.008 Medline
53. H. Reijonen, W. W. Kwok, Use of HLA class II tetramers in tracking antigen-specific T cells and mapping T-cell epitopes. *Methods* **29**, 282–288 (2003). doi:10.1016/S1046-2023(02)00350-X Medline
54. S. K. Saini, T. Tamhane, R. Anjanappa, A. Saikia, S. Ramskov, M. Donia, I. M. Svane, S. N. Jakobsen, M. Garcia-Alai, M. Zacharias, R. Meijers, S. Springer, S. R. Hadrup, Empty peptide-receptive MHC class I molecules for efficient detection of antigen-specific T cells. *Sci. Immunol.* **4**, eaau9039 (2019). doi:10.1126/sciimmunol.aau9039 Medline
55. T. J. Scriba, M. Purbhoo, C. L. Day, N. Robinson, S. Fidler, J. Fox, J. N. Weber, P. Klenerman, A. K. Sewell, R. E. Phillips, Ultrasensitive detection and phenotyping of CD4+ T cells with optimized HLA class II tetramer staining. *J. Immunol.* **175**, 6334–6343 (2005). doi:10.4049/jimmunol.175.10.6334 Medline
56. P. Serra, N. Garabatos, S. Singha, C. Fandos, J. Garnica, P. Solé, D. Parras, J. Yamanouchi, J. Blanco, M. Tort, M. Ortega, Y. Yang, K. K. Ellestad, P. Santamaria, Increased yields and biological potency of knob-into-hole-based soluble MHC class II molecules. *Nat. Commun.* **10**, 4917 (2019). doi:10.1038/s41467-019-12902-2 Medline
57. P. Zimmermann, N. Curtis, Coronavirus Infections in Children Including COVID-19: An Overview of the Epidemiology, Clinical Features, Diagnosis, Treatment and Prevention Options in Children. *Pediatr. Infect. Dis. J.* **39**, 355–368 (2020). doi:10.1097/INF.0000000000002660 Medline
58. N. Principi, S. Bosis, S. Esposito, Effects of coronavirus infections in children. *Emerg. Infect. Dis.* **16**, 183–188 (2010). doi:10.3201/eid1602.090469 Medline
59. B. Agerer, M. Koblischke, V. Gudipati, L. F. Montaño-Gutierrez, M. Smyth, A. Popa, J.-W. Genger, L. Endler, D. M. Florian, V. Mühlgrabner, M. Graninger, S. W. Aberle, A.-M. Husa, L. E. Shaw, A. Lercher, P. Gattinger, R. Torralba-Gombau, D. Trapin, T. Penz, D. Barreca, I. Fae, S. Wenda, M. Traugott, G. Walder, W. F. Pickl, V. Thiel, F. Allerberger, H. Stockinger, E. Puchhammer-Stöckl, W. Weninger, G. Fischer, W. Hoepler, E. Pawelka, A. Zoufaly, R. Valenta, C. Bock, W. Paster, R. Geyeregger, M. Farlik, F. Halbritter, J. B. Huppa, J. H. Aberle, A. Bergthaler, SARS-CoV-2 mutations in MHC-I-restricted epitopes evade $CD8^+$ T cell responses. *Sci. Immunol.* **6**, eabg6461 (2021). doi:10.1126/sciimmunol.abg6461 Medline
60. A. Tarke, J. Sidney, N. Methot, Y. Zhang, J. M. Dan, B. Goodwin, P. Rubiro, A. Sutherland, R. da Silva Antunes, A. Frazier, S. A. Rawlings, D. M. Smith, B. Peters, R. H. Scheuermann, D. Weiskopf, S. Crotty, A. Grifoni, A. Sette, Negligible impact of SARS-CoV-2 variants on $CD4^+$ and $CD8^+$ T cell reactivity in COVID-19 exposed donors and vaccinees. *bioRxiv* 2021.02.27.433180 (2021). Medline
61. A. Peisley, G. Skiniotis, 2D Projection Analysis of GPCR Complexes by Negative Stain Electron Microscopy. *Methods Mol. Biol.* **1335**, 29–38 (2015). doi:10.1007/978-1-4939-2914-6_3 Medline
62. M. H. Gee, A. Han, S. M. Lofgren, J. F. Beausang, J. L. Mendoza, M. E. Birnbaum, M. T. Bethune, S. Fischer, X. Yang, R. Gomez-Eerland, D. B. Bingham, L. V. Sibener, R. A. Fernandes, A. Velasco, D. Baltimore, T. N. Schumacher, P. Khatri, S. R. Quake, M. M. Davis, K. C. Garcia, Antigen Identification for Orphan T Cell Receptors Expressed on Tumor-Infiltrating Lymphocytes. *Cell* **172**, 549–563.e16 (2018). doi:10.1016/j.cell.2017.11.043 Medline
63. M. Toebes, M. Coccoris, A. Bins, B. Rodenko, R. Gomez, N. J. Nieuwkoop, W. van de Kasteele, G. F. Rimmelzwaan, J. B. A. G. Haanen, H. Ovaa, T. N. M. Schumacher, Design and use of conditional MHC class I ligands. *Nat. Med.* **12**, 246–251 (2006). doi:10.1038/nm1360 Medline
64. G. Chen, D. Wu, W. Guo, Y. Cao, D. Huang, H. Wang, T. Wang, X. Zhang, H. Chen, H. Yu, X. Zhang, M. Zhang, S. Wu, J. Song, T. Chen, M. Han, S. Li, X. Luo, J. Zhao, Q. Ning, Clinical and immunological features of severe and moderate coronavirus disease 2019. *J. Clin. Invest.* **130**, 2620–2629 (2020). doi:10.1172/JCI137244 Medline
65. P. Stothard, The sequence manipulation suite: JavaScript programs for analyzing and formatting protein and DNA sequences. *Biotechniques* **28**, 1102, 1104 (2000). doi:10.2144/00286ir01 Medline
66. Y. Su, D. Chen, D. Yuan, C. Lausted, J. Choi, C. L. Dai, V. Voillet, V. R. Duvvuri, K. Scherler, P. Troisch, P. Baloni, G. Qin, B. Smith, S. A. Kornilov, C. Rostomily, A. Xu, J. Li, S. Dong, A. Rothchild, J. Zhou, K. Murray, R. Edmark, S. Hong, J. E. Heath, J. Earls, R. Zhang, J. Xie, S. Li, R. Roper, L. Jones, Y. Zhou, L. Rowen, R. Liu, S. Mackay, D. S. O'Mahony, C. R. Dale, J. A. Wallick, H. A. Algren, M. A. Zager, W. Wei, N. D. Price, S. Huang, N. Subramanian, K. Wang, A. T. Magis, J. J. Hadlock, L. Hood, A. Aderem, J. A. Bluestone, L. L. Lanier, P. D. Greenberg, R. Gottardo, M. M. Davis, J. D. Goldman, J. R. Heath; ISB-Swedish COVID19 Biobanking Unit, Multi-Omics Resolves a Sharp Disease-State Shift between Mild and Moderate COVID-19. *Cell* **183**, 1479–1495.e20 (2020). doi:10.1016/j.cell.2020.10.037 Medline

Acknowledgments: We thank all the volunteers and patients for their participation in this study. We thank the CROWN clinic staff, Stanford Occupational Health staff, and Sean N Parker clinical research unit for enrolling volunteers. We thank Alexandra Skye Lee for maintaining the COVID-19 patient database. We thank members from the laboratories of Mark Davis and Yueh-Hsiu Chien for helpful discussions. We appreciate the feedback and suggestions from Drs. Yueh-Hsiu Chien, Roshni Roy Chowdhury, Lisa Wagar and Masaru Kanekiyo. Cell sorting and flow cytometry analysis for this project was done on instruments in the Stanford Shared FACS Facility. We thank Dr. Naresha Saligrama for providing the TCR3 cell line, and Dr. Elizabeth Mellins for providing access to the Octet QK system. We also thank Dr. Yamuna Kalyani Mathiharan for assistance with negative-stain EM. **Funding:** This work was supported by grants from the

Howard Hughes Medical Institute and NIAID grant AI057229 to MMD. Additional support was provided by the Bill and Melinda Gates Foundation to MMD (OPP1113682, Center for Human Systems Immunology) and ITI-YIA to VM. Further funds were provided by the Sean N Parker Center, the Sunshine Foundation, and U01 AI140498 to MM and KCN. **Author contributions:** Project conceptualization and study design was performed by VM and MMD. Experiments and data analyses was performed by VM and CG. SC assisted with binding experiments and data analyses. AMM and JW performed single-cell TCR sequencing. AMM assisted with antigen-specificity validation assays. AJP assisted with GLIPH2 analysis. AN, MM and KCN provided samples and reagents. VM, CG and MMD wrote the manuscript with input from all the authors. **Competing interests:** VM and MMD are inventors on a patent application on the spheromer technology described in this work. The other authors declare that they have no competing interests. **Data and materials availability:** All data needed to evaluate the conclusions in the paper are present in the paper or the Supplementary Materials. The reagents required for spheromer assembly will be made available from the corresponding author upon completion of a standard material transfer agreement (MTA) in accordance with Stanford technology transfer policy. This work is licensed under a Creative Commons Attribution 4.0 International (CC BY 4.0) license, which permits unrestricted use, distribution, and reproduction in any medium, provided the original work is properly cited. To view a copy of this license, visit https://creativecommons.org/licenses/by/4.0/. This license does not apply to figures/photos/artwork or other content included in the article that is credited to a third party; obtain authorization from the rights holder before using this material.

Submitted 14 January 2021
Accepted 28 June 2021
Published First Release 1 July 2021
10.1126/sciimmunol.abg5669

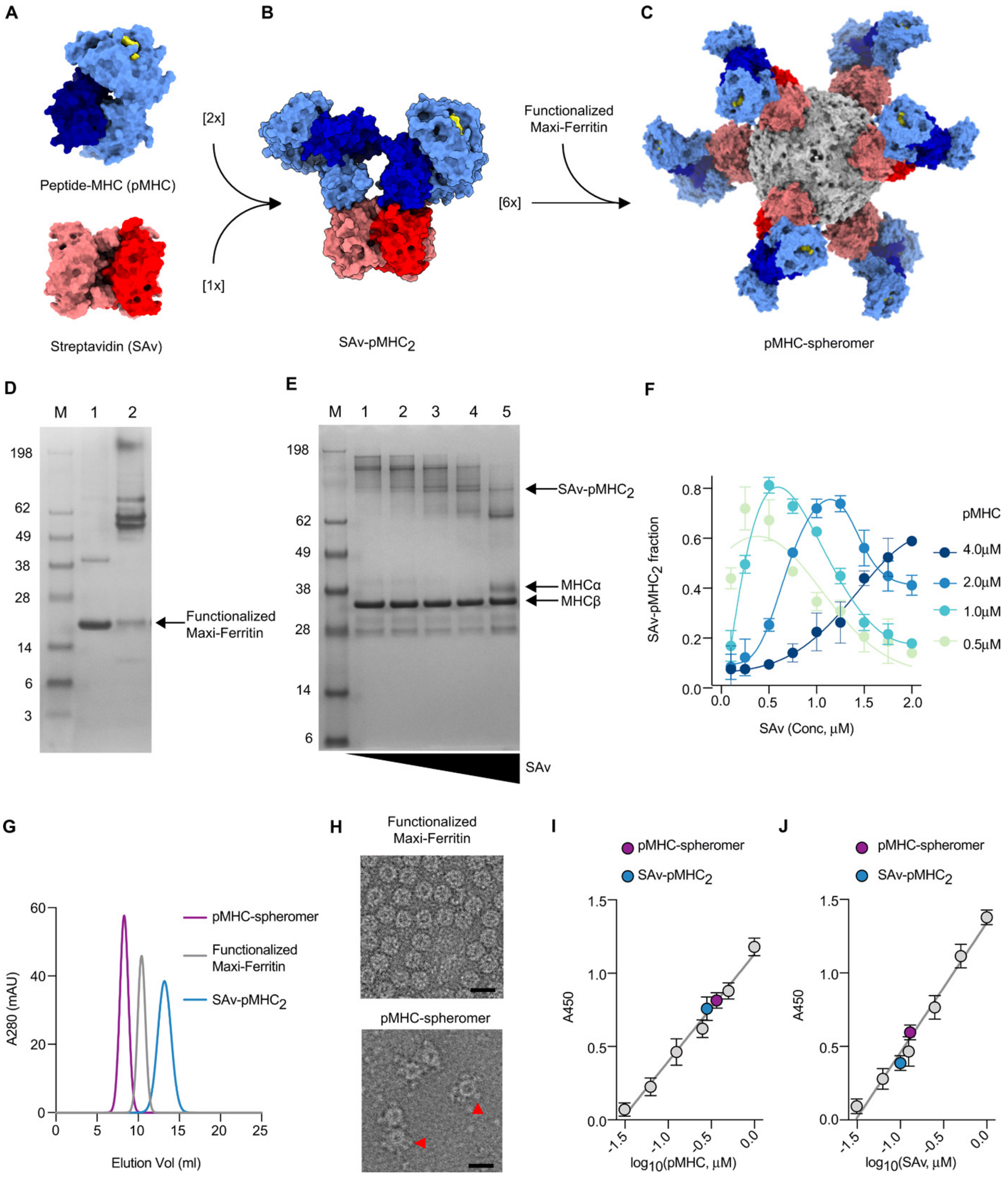
A
B
C
Peptide-MHC (pMHC)
Streptavidin (SAv)
[2x]
[1x]
SAv-pMHC2
Functionalized
Maxi-Ferritin
[6x]
pMHC-spheromer
D
M 1 2
198
62
49
38
28
14
6
3
Functionalized
Maxi-Ferritin
E
M 1 2 3 4 5
SAv-pMHC2
MHCα
MHCβ
SAv
F
SAv-pMHC2 fraction
SAv (Conc, μM)
pMHC
4.0μM
2.0μM
1.0μM
0.5μM
G
A280 (mAU)
Elution Vol (ml)
pMHC-spheromer
Functionalized
Maxi-Ferritin
SAv-pMHC2
H
Functionalized
Maxi-Ferritin
pMHC-spheromer
I
pMHC-spheromer
SAv-pMHC2
A450
log10(pMHC, μM)
J
pMHC-spheromer
SAv-pMHC2
A450
log10(SAv, μM)

Fig. 1. Assembly and characterization of the "spheromer". (A) Molecular surface representation of pMHC (PDB ID: 3TO2, α-chain in light blue, β2m in dark blue and peptide in yellow) and SAv (PDB ID: 2RTG, monomer in red and rest in coral). **(B)** Model of a semi-saturated SAv-pMHC$_2$ intermediate that has two unoccupied biotin binding sites. A single orientation is shown for simplicity. **(C)** A model of spheromer that is assembled by the conjugation of six semi-saturated SAv-pMHC$_2$ molecules onto a functionalized maxi-ferritin scaffold (PDB ID:2JD6, grey). UCSF Chimera was used for molecular graphics. **(D)** Streptavidin gel-shift assay to evaluate the functionalization of maxi-ferritin. Lane 1: Biotinylated maxi-ferritin scaffold. The protein dissociates into monomers (23.4 kDa) after boiling and migrates at the corresponding size on a denaturing gel. Lane 2: The flexible tether engineered at the N terminus of each monomer has one biotin binding site. Upon incubation with SAv, migration of the biotinylated maxi-ferritin monomers is retarded due to formation of a complex. **(E)** The formation of semi-saturated SAv-pMHC$_2$ monitored by streptavidin gel-shift assay. The MHC α-chain is biotinylated and shifts upon binding SAv. The pMHC was incubated with limiting concentrations of SAv resulting in the formation of oligomers with incremental increase in valency. SDS-PAGE is shown for the titration of an MHC-II molecule with SAv. **(F)** Quantification of SAv-pMHC$_2$ formation as a function of pMHC and SAv reactant concentrations. The mean±SD of the measurements from three experiments is shown. **(G)** Size-exclusion chromatogram of the spheromer and its components. **(H)** Representative electron micrographs of negatively stained maxi-ferritin and spheromer. The SAv-pMHC$_2$ conjugated to the surface of the functionalized scaffold are indicated by red arrows. Scale bars, 20nm. Validation of SAv-pMHC$_2$ conjugation to the spheromer using **(I)** anti-MHC **(J)** anti-streptavidin antibodies by ELISA (mean±SD). The experiment was performed with each sample in triplicates and repeated at least twice.

A

TCR features						Antigen description		
**TCR ID**	**HLA restriction**	**TRBV**	**CDR3β**	**TRAV**	**CDR3α**	**Source**	**Protein**	**Epitope**
TCR1	A*02:01	TRBV19	CASSYSISYEQYF	TRAV12-3	CAMSSGGTSYGKLTF	Azospirillum	BHW58	WLDGVTPSL
TCR2	A*02:01	TRBV6-5	CASSPVTGGIYGYTF	TRAV24	CARNTGNQFYF	HCMV	pp65	NLVPMVATV
TCR3	DRB1*15:01	TRBV14	CASSHNSYEQYF	TRAV9-2	CALTLQNRDDKIIF	Adenovirus	Protein III	ATFTSYRSWYLA
TCR4	DRB1*04:01	TRBV28	CASSSTGLPYGYTF	TRAV8-4	CAVSESPFGNEKLT	Influenza	HA	PKYVKQNTLKLAT

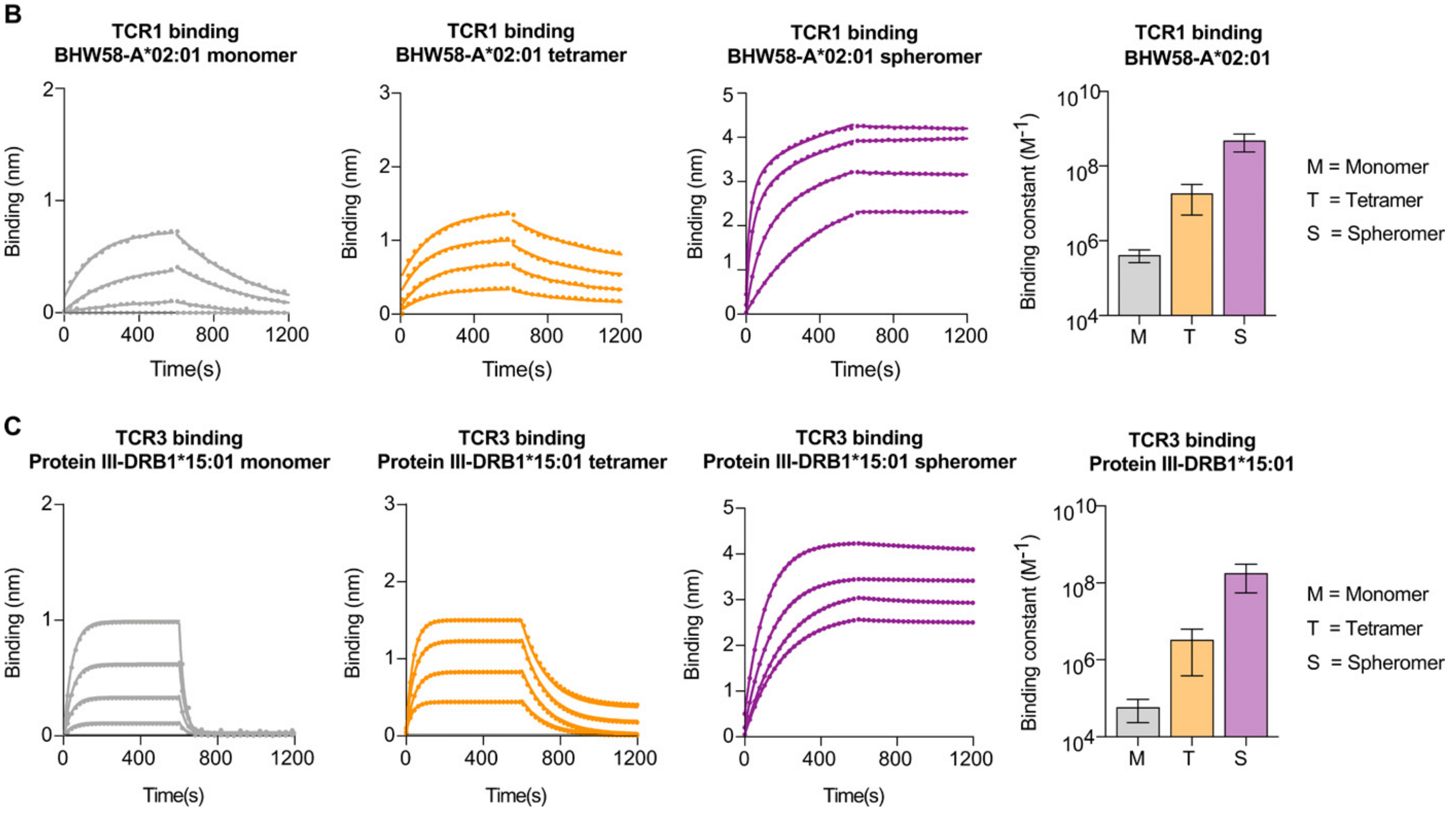

Fig. 2. Spheromer binds both MHC-I and MHC-II restricted TCRs with high avidity. (A) List of evaluated pMHC-TCR pairs. The binding of **(B)** TCR1 and **(C)** TCR3 to different formulations of BHW58-A*02:01 and Protein III-DRB1*15:01 respectively was determined by biolayer interferometry. An overlay of binding traces over a concentration series of the indicated pMHC formulation from one representative experiment is shown. Each binding experiment was repeated at least three times. The mean±SD of the binding constant has been graphed.

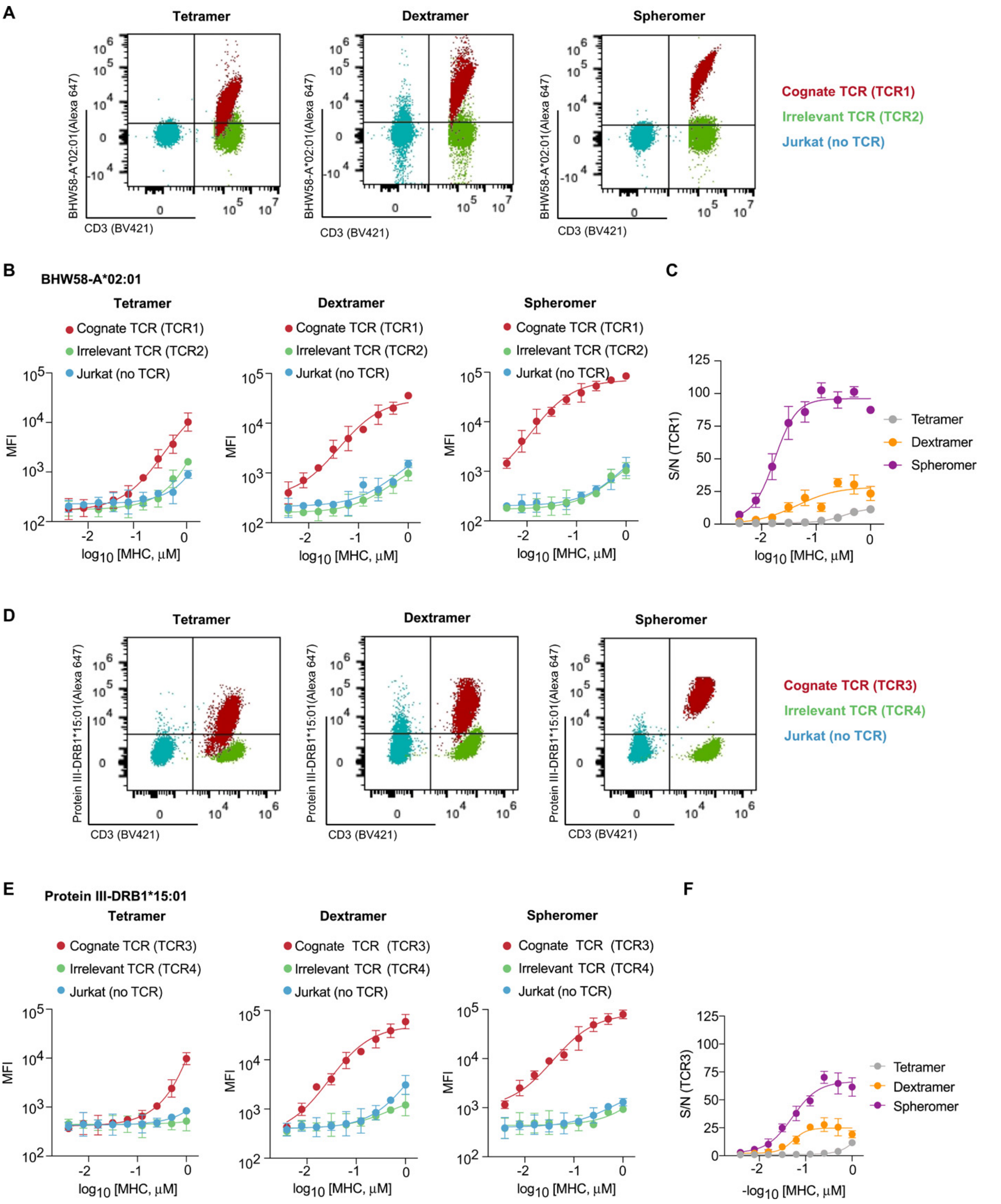
A
Tetramer
Dextramer
Spheromer
BHW58-A*02:01(Alexa 647)
CD3 (BV421)
Cognate TCR (TCR1)
Irrelevant TCR (TCR2)
Jurkat (no TCR)
B
BHW58-A*02:01
C
MFI
log10 [MHC, μM]
S/N (TCR1)
D
Protein III-DRB1*15:01(Alexa 647)
Cognate TCR (TCR3)
Irrelevant TCR (TCR4)
E
Protein III-DRB1*15:01
F
S/N (TCR3)
-log10 [MHC, μM]

Fig. 3. Spheromer binds T cell lines expressing MHC-I or MHC-II restricted TCRs with high specificity. (A) Representative flow cytometry plots showing the binding of the indicated BHW58-A*02:01 formulations with equivalent pMHC concentration to a T cell line expressing TCR1. The non-specific binding of the different formulations was measured using untransduced Jurkat cells and a cell line expressing an irrelevant TCR. CD3 was measured as proxy for TCR expression. **(B)** Quantification of BHW58-A*02:01 binding measured by flow cytometry (mean±SD). The experiment was performed with each sample processed in duplicates and repeated at least twice. **(C)** The signal-to-noise ratio (S/N) of TCR1 binding to distinct BHW58-A*02:01 multivalent formulations. Mean±SD of the measurements from two independent experiments has been plotted. **(D)** Representative flow cytometry plots showing the binding of the indicated Protein III-DRB1*15:01 formulations with equivalent pMHC concentration to a T cell line expressing TCR3. The non-specific binding to Jurkat cells and an irrelevant TCR was also measured. CD3 was measured as proxy for TCR expression. **(E)** Quantification of Protein III-DRB1*15:01 binding (mean±SD) measured by flow cytometry. **(F)** The signal-to-noise ratio (S/N) of TCR3 binding to distinct Protein III-DRB1*15:01 multivalent formulations (mean±SD).

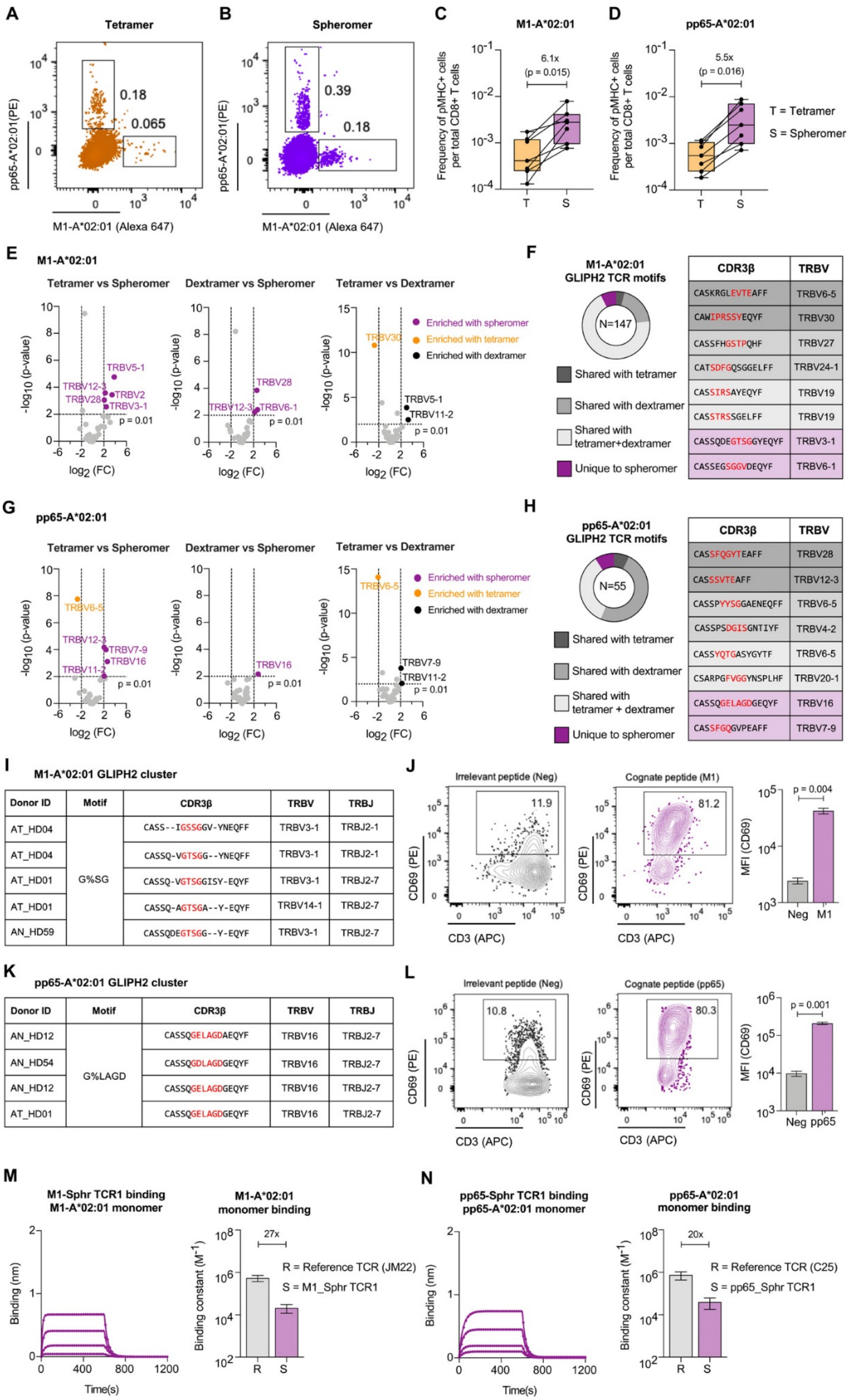

CDR3β	TRBV
CASKRGLEVTEAFF	TRBV6-5
CAWIPRSSYEQYF	TRBV30
CASSFHGSTPQHF	TRBV27
CATSDFGQSGGELFF	TRBV24-1
CASSIRSAYEQYF	TRBV19
CASSTRSSGELFF	TRBV19
CASSQDEGTSGGYEQYF	TRBV3-1
CASSEGSGGVDEQYF	TRBV6-1

CDR3β	TRBV
CASSFQGYTEAFF	TRBV28
CASSSVTEAFF	TRBV12-3
CASSPYYSGGAENEQFF	TRBV6-5
CASSPSDGISGNTIYF	TRBV4-2
CASSYQTGASYGYTF	TRBV6-5
CSARPGFVGGYNSPLHF	TRBV20-1
CASSQGELAGDGEQYF	TRBV16
CASSFGQGVPEAFF	TRBV7-9

Donor ID	Motif	CDR3β	TRBV	TRBJ
AT_HD04	G%SG	CASS--IGSSGGV-YNEQFF	TRBV3-1	TRBJ2-1
AT_HD04		CASSQ-VGTSGG--YNEQFF	TRBV3-1	TRBJ2-1
AT_HD01		CASSQ-VGTSGGISY-EQYF	TRBV3-1	TRBJ2-7
AT_HD01		CASSQ-AGTSGA--Y-EQYF	TRBV14-1	TRBJ2-7
AN_HD59		CASSQDEGTSGG--Y-EQYF	TRBV3-1	TRBJ2-7

Donor ID	Motif	CDR3β	TRBV	TRBJ
AN_HD12	G%LAGD	CASSQGELAGDAEQYF	TRBV16	TRBJ2-7
AN_HD54		CASSQGDLAGDGEQYF	TRBV16	TRBJ2-7
AN_HD12		CASSQGELAGDGEQYF	TRBV16	TRBJ2-7
AT_HD01		CASSQGELAGDGEQYF	TRBV16	TRBJ2-7

Fig. 4. Spheromer detects a higher frequency of antigen-specific T cells with a more diverse TCR repertoire. Representative flow cytometry plots of CD8$^+$ T cells isolated from HLA-A*02:01 individuals stained with influenza-M1 and HCMV-pp65 **(A)** Tetramers or **(B)** Spheromers. Enumeration of epitope-specific **(C)** M1 and **(D)** pp65 CD8$^+$ T cells detected in healthy individuals using either tetramer or spheromer. Data from each donor (n=7) is represented by a point. A two-tailed, matched-pairs Wilcoxon signed-rank test was performed to determine the significance levels. **(E)** Volcano plots showing the variance in TRBV usage of M1-A*02:01 specific CD8$^+$ T cells detected using the spheromer and other pMHC multimers. The TRBV genes enriched significantly (p-value ≤ 0.01, Fisher's exact test) with the spheromer are highlighted in purple. **(F)** The distribution of spheromer derived, influenza-M1 specific TCR motifs identified by GLIPH2 and representative examples from each category. **(G)** Volcano plots representing the variance in TRBV usage of pp65-A*02:01 specific CD8$^+$ T cells detected with distinct pMHC multimers. The TRBV genes enriched significantly (p-value ≤ 0.01, Fisher's exact test) with the spheromer are highlighted in plum. **(H)** The distribution of spheromer derived, HCMV-pp65 specific TCR motifs identified by GLIPH2 and representative examples from each category. **(I)** A representative GLIPH2 cluster with specificity for influenza-M1 composed of TCR sequences identified exclusively using the spheromer. **(J)** Representative flow cytometry plots showing the activation of a T cell line (expressing a TCR with "G%SG" motif) stimulated with an irrelevant or cognate (influenza-M1) peptide. The activation was measured by CD69 expression. The significance level was determined by a two-tailed, paired *t* test. **(K)** A representative GLIPH2 cluster with specificity for HCMV-pp65 that is comprised of spheromer derived TCR sequences exclusively. **(L)** Representative flow cytometry plots showing the activation of a T cell line (expressing a TCR with "G%LAGD" motif) stimulated with an irrelevant or cognate (HCMV-pp65) peptide. The activation was measured by CD69 expression. A two-tailed, paired *t* test was performed to determine significance. The binding of TCR corresponding to clones from GLIPH2 clusters comprised exclusively of spheromer derived sequences to their cognate pMHC monomers **(M)** M1-A*02:01 and **(N)** pp65-A*02:01 determined by biolayer interferometry. Each binding experiment was repeated at least thrice. The mean±SD of the binding constant has been graphed and compared to a reference influenza-M1 (JM22) and HCMV-pp65 (C25) specific TCR.

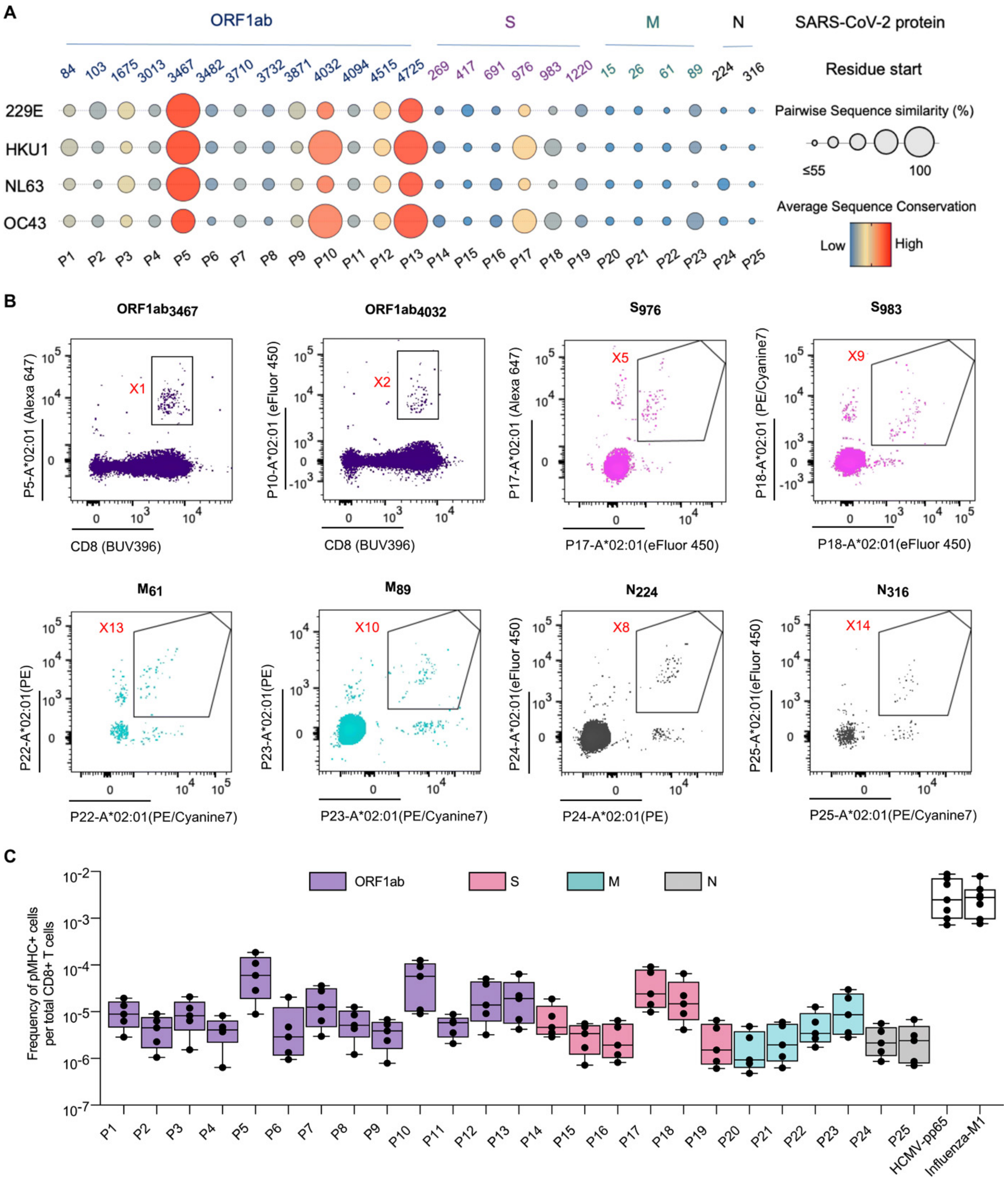
A
ORF1ab
S
M
N
SARS-CoV-2 protein
Residue start
84 103 1675 3013 3467 3482 3710 3732 3871 4032 4094 4515 4725 269 417 691 976 983 1220 15 26 61 89 224 316
229E
HKU1
NL63
OC43
P1 P2 P3 P4 P5 P6 P7 P8 P9 P10 P11 P12 P13 P14 P15 P16 P17 P18 P19 P20 P21 P22 P23 P24 P25
Pairwise Sequence similarity (%)
≤55
100
Average Sequence Conservation
Low
High
B
ORF1ab3467
ORF1ab4032
S976
S983
X1
X2
X5
X9
P5-A*02:01 (Alexa 647)
P10-A*02:01 (eFluor 450)
P17-A*02:01 (Alexa 647)
P18-A*02:01 (PE/Cyanine7)
CD8 (BUV396)
CD8 (BUV396)
P17-A*02:01(eFluor 450)
P18-A*02:01(eFluor 450)
M61
M89
N224
N316
X13
X10
X8
X14
P22-A*02:01(PE)
P23-A*02:01(PE)
P24-A*02:01(eFluor 450)
P25-A*02:01(eFluor 450)
P22-A*02:01(PE/Cyanine7)
P23-A*02:01(PE/Cyanine7)
P24-A*02:01(PE)
P25-A*02:01(PE/Cyanine7)
C
ORF1ab
S
M
N
Frequency of pMHC+ cells per total CD8+ T cells
P1 P2 P3 P4 P5 P6 P7 P8 P9 P10 P11 P12 P13 P14 P15 P16 P17 P18 P19 P20 P21 P22 P23 P24 P25 HCMV-pp65 Influenza-M1

Fig. 5. The frequency of CD8$^+$ T cells against SARS-CoV-2 epitopes conserved across seasonal hCoVs are elevated in unexposed individuals. (A) The sequence conservation of SARS-CoV-2 epitopes across seasonal hCoVs. The epitopes were selected based on their biochemical properties and binding to HLA-A*02:01. These peptides span multiple SARS-CoV-2 coding regions (ORF1ab, S, M and N) and display varying degrees of sequence similarity. The pairwise conservation score between SARS-CoV-2 and any given hCoV is indicated by the size of the bubble. The color represents the average conservation score across all hCoVs. **(B)** Representative flow cytometry plots of combinatorial, antigen-specific staining of PBMCs from an unexposed individual using HLA-A*02:01 spheromer pools after magnetic enrichment. The fluorophore barcode as shown in the supplementary information used to determine antigen specificity is labeled in red next to the gated population. **(C)** The enumeration of SARS-CoV-2 epitope specific CD8$^+$ T cells in unexposed, pre-pandemic PBMC samples collected between April 2018 – Feb 2019. Data from each donor (n=5) is represented by a dot. The frequency of SARS-CoV-2 specific T cells in unexposed individuals is lower than HCMV-pp65 and influenza-M1 specific T cells.

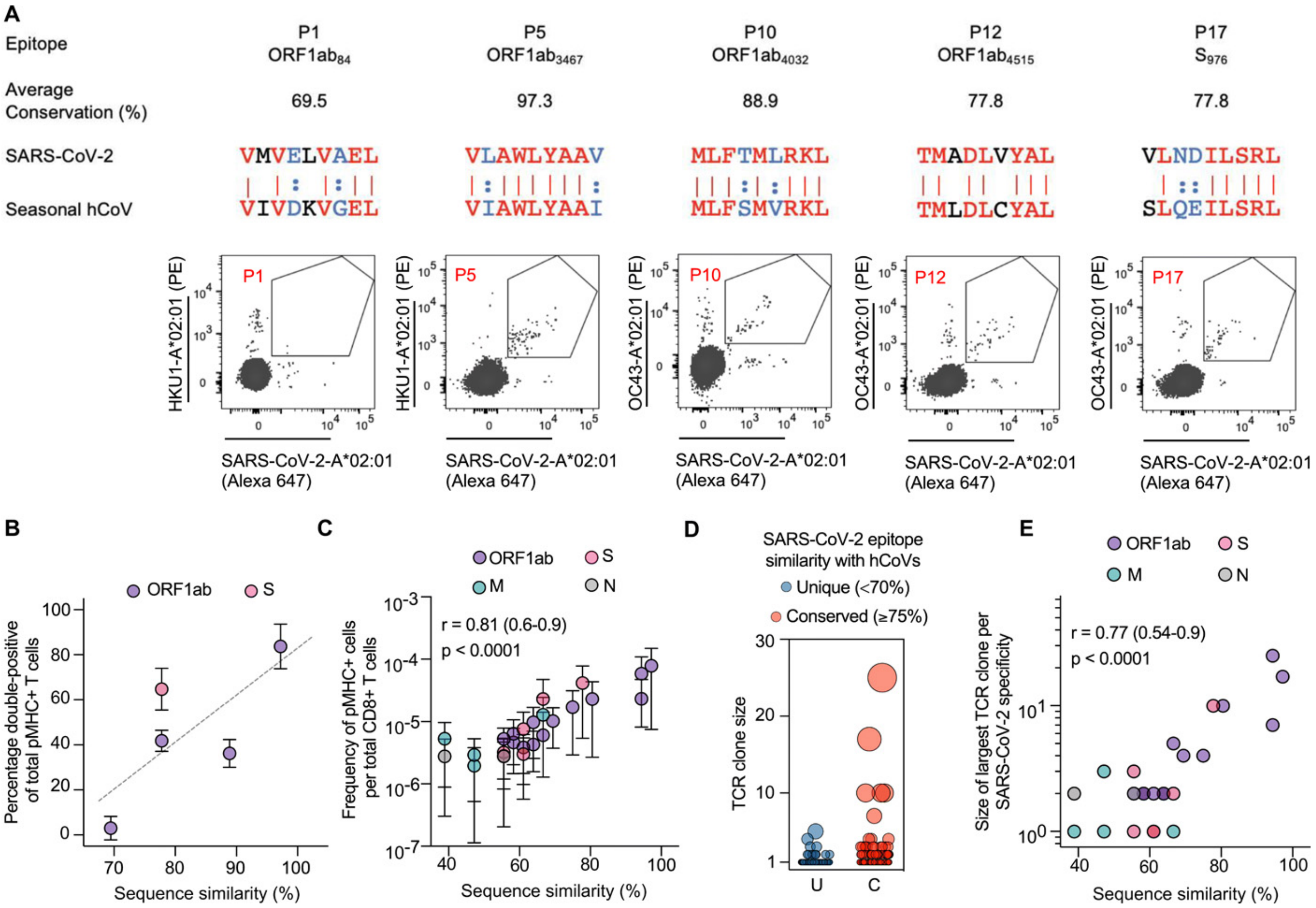

Fig. 6. Cross-reactivity between SARS-CoV-2 and seasonal hCoV CD8⁺ T cell epitopes. (A) Representative flow cytometry plots showing the co-staining of CD8⁺ T cells from unexposed individuals using spheromers displaying the indicated SARS-CoV-2 and seasonal hCoV A*02:01 bound peptides after magnetic enrichment. The average conservation score across all hCoVs for each epitope is listed. For the pairwise sequence comparison: identical residues (red), synonymous residues defined in our substitution matrix (blue), rest (black). **(B)** Correlation between the fraction of co-stained CD8⁺ T cells and the average sequence similarity of SARS-CoV-2 epitopes with hCoVs in healthy, unexposed individuals (n=3). **(C)** A positive correlation was observed between the average sequence similarity of SARS-CoV-2 epitopes with hCoVs and the baseline frequency of SARS-CoV-2 epitope specific CD8⁺ T cells in healthy, unexposed individuals. **(D)** Evaluation of clonal expansion in unexposed individuals using single-cell TCR sequencing of SARS-CoV-2 specific CD8⁺ T cells identified using spheromer. A summary plot of TCR clonality across all SARS-CoV-2 epitopes tested in this study. The data was divided into 2 groups (unique or conserved) based on a threshold of ≥75% (allowing for 2 mismatches in a given 9-mer). Each individual dot represents a distinct TCR clone. **(E)** Correlation between the average sequence similarity of SARS-CoV-2 epitopes with hCoVs and size of the largest TCR clone of the corresponding specificity.

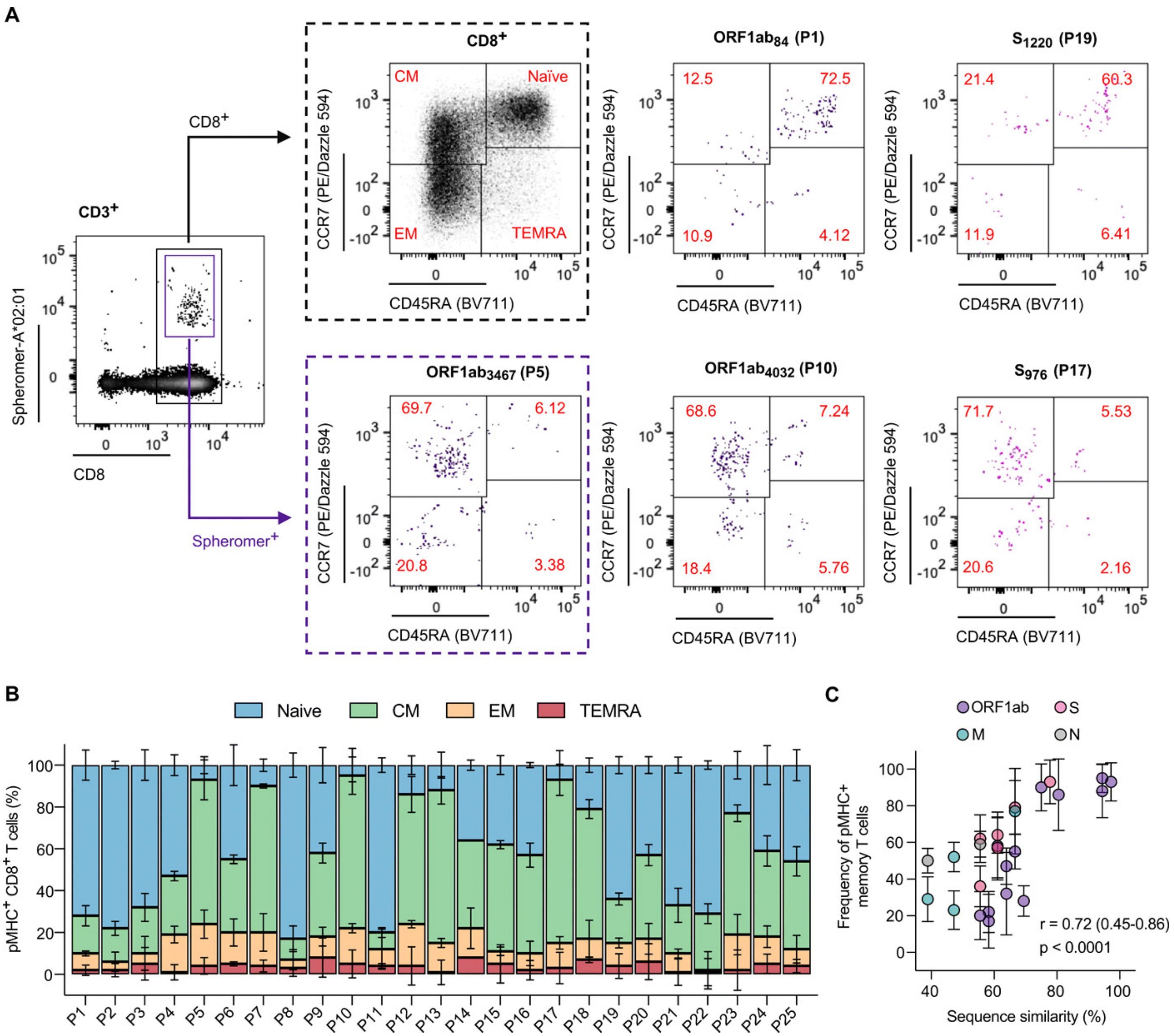

Fig. 7. CD8⁺ T cells in unexposed individuals against conserved SARS-CoV-2 epitopes exhibit a predominant memory phenotype. (A) Representative flow cytometry plots showing the distribution of SARS-CoV-2 specific CD8⁺ T cells across the naïve and memory subsets defined based on the expression of CD45RA and CCR7 markers; naïve (CD45RA⁺CCR7⁺), central memory (CM, CD45RA⁻CCR7⁺), effector memory (EM, CD45RA⁻CCR7⁻), and effector memory expressing CD45RA (TEMRA, CD45RA⁺CCR7⁻) in healthy, unexposed individuals. The antigen-specific CD8⁺ T cells were enriched using magnetic beads. **(B)** Quantification of SARS-CoV-2 specific CD8⁺ T cells across the naïve and memory subsets in healthy, unexposed individuals. **(C)** Correlation between the average sequence similarity of SARS-CoV-2 epitopes across hCoVs and the frequency of memory (non-naïve) CD8⁺ T cells.

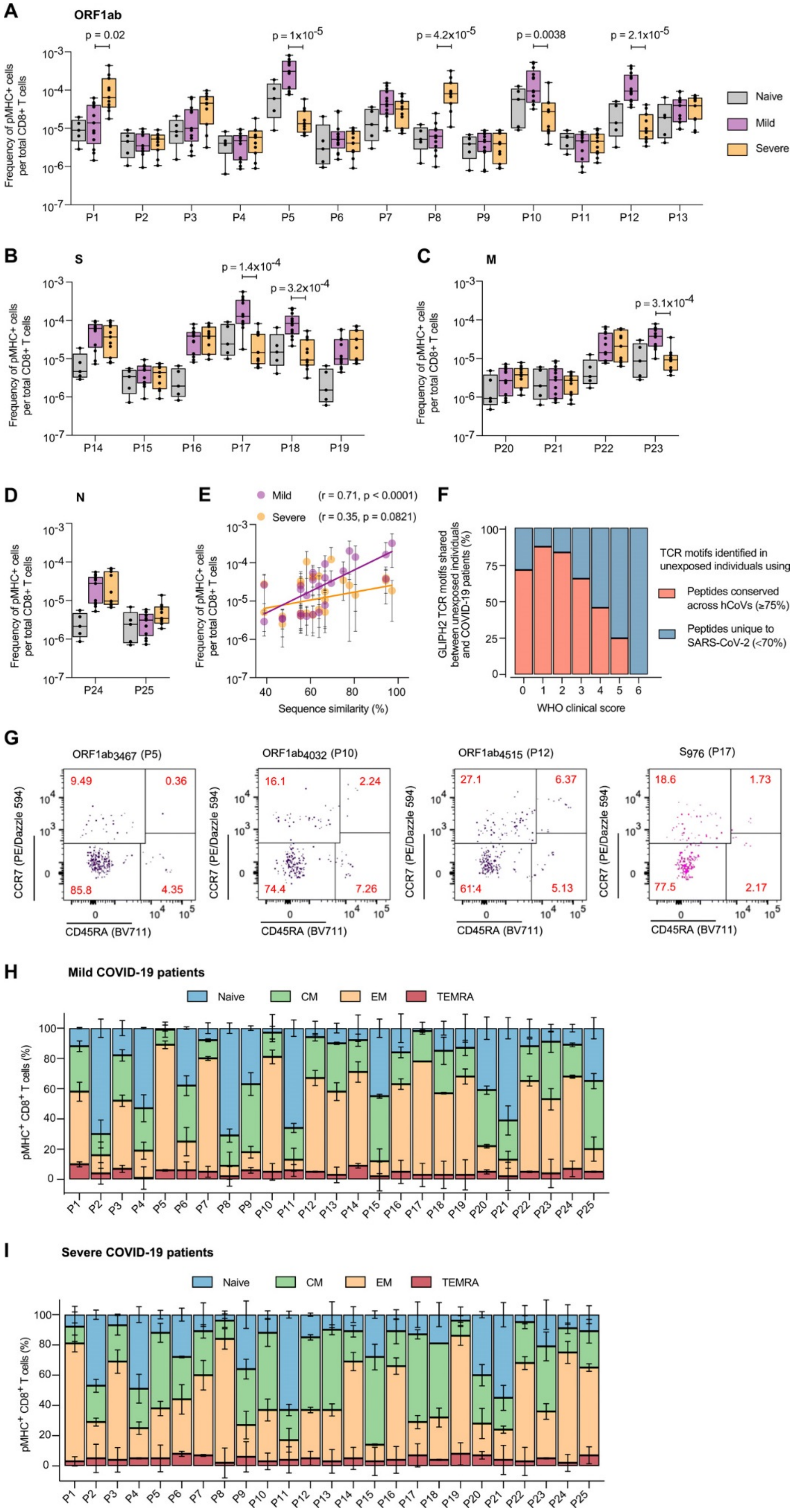

Fig. 8. COVID-19 patients with divergent clinical outcomes exhibit distinct SARS-CoV-2 epitope specific CD8⁺ T cell responses. The frequency of SARS-CoV-2 epitope specific CD8⁺ T cells across unexposed individuals and COVID-19 patients with mild (n=13) and severe (n=11) infections: **(A)** ORF1ab, **(B)** S, **(C)** M and **(D)** N. The adjusted p-value as determined by Dunn's test corrected for multiple comparisons is reported for specificities with a significant difference between mild and severe COVID-19 patients. **(E)** Correlation between the average sequence similarity of SARS-CoV-2 epitopes across hCoVs and the frequency of antigen-specific CD8⁺ T cells in COVID-19 patients. **(F)** The distribution of SARS-CoV-2 specific TCR motifs shared between unexposed individuals and COVID-19 patients. TCR motifs were identified using GLIPH2. A lower WHO score indicates milder symptoms. TCR motifs shared between unexposed individuals and mild COVID-19 patients were identified by conserved SARS-CoV-2 epitopes. In contrast, TCR motifs characterizing severe COVID-19 patients were detected in unexposed individuals using peptides that were primarily unique to SARS-CoV-2 (adjusted p-value = 0.00019, Fisher's test). **(G)** Representative flow cytometry plots showing the distribution of SARS-CoV-2 specific CD8⁺ T cells across the naïve and memory subsets in COVID-19 patients. The antigen-specific CD8⁺ T cells were enriched using magnetic beads. Quantification of SARS-CoV-2 specific CD8⁺ T cells across the naïve and memory subsets in **(H)** mild and **(I)** severe COVID-19 patients.
