# Supplementary material for: CD8+ T cells specific for conserved coronavirus epitopes correlate with milder disease in patients with COVID-19
Source: Sci Immunol. 2021 Jul 1;6(61):eabg5669. doi: 10.1126/sciimmunol.abg5669 (PMC8975171; doi:10.1126/sciimmunol.abg5669)
Supplement: Supplementary file 2 — Figs. S1 to S12 Tables S1 to S3 [file sciimmunol.abg5669_sm.pdf]

Science Immunology
AAAS

Supplementary Materials for

CD8$^+$ T cells specific for conserved coronavirus epitopes correlate with milder disease in patients with COVID-19

Vamsee Mallajosyula *et al*.

Corresponding author: Mark M. Davis, mmdavis@stanford.edu

Sci. Immunol. **6**, eabg5669 (2021)
DOI: 10.1126/sciimmunol.abg5669

The PDF file includes:

Figs. S1 to S12
Tables S1 to S3

Other Supplementary Material for this manuscript includes the following:

Data file S1
MDAR Checklist

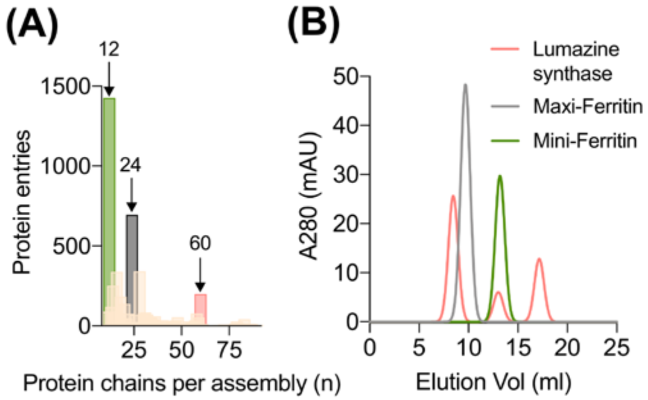

(C)

Protein	n	Yield (mg/L)	Homogeneity
DNA Protection during Stress Protein (Mini-Ferritin)	12	3.8	≥95%
Nonheme ferritin (Maxi-Ferritin)	24	7.5	≥95%
Lumazine synthase	60	1.2	58%

Figure S1: Selection and characterization of scaffold candidates. (A) The distribution of higher-order (n>10) oligomeric assemblies across all non-redundant entries (N=145,181) in the protein data bank. A preference for specific oligomeric states (n=12, 24 and 60) was observed. **(B)** Size-exclusion profile of purified proteins selected for developing a scaffold for the multivalent display of pMHC. **(C)** Summary table listing the yield and homogeneity for the scaffold candidates.

(A)

Linker ID	Sequence
L1	G_3S
L2	$(G_3S)_3$
L3	$(G_3S)_6$
L4	S_2G
L5	$(S_2G)_3$
L6	$(SG_2P)_2SG_2$
L7	$(S_2G)_3SPVG_2$
L8	$(G_2S)_2SPV(STP_2TPSP)_2G_2S$
L9	$(G_2S)_2SPV(STP_2TPSP)_4G_2S$
L11	$G_2S(GSP)_3G_2S$
L12	$S_2G(EA_3K)_3S_2G$
L13	$S_2GEA_3KALEAEA_3KS_2G$
L14	$(GS_2P)_3G_3S$
L15	$GA_2PA_3PAKQEA_3PAPA_2KAEAPA_3PA_2KA$
L16	$S_2G(G_2PQ)_3S_2G$
L17	$G_2S_2PG_2S_2$
L18	$G_2S_2PG_2S_2PG_2S_2PG_2S_2$
L19	$KLSG_4SG_4SG_4SAEAWYNLGNAY_2KQGDYQ$ $KAIEY_2QKALELDPN_2LQRSAG_4SG_4SG_4AS$

(B) MPMGSLQPLATLYLLGMLVASCLGGLNDIFEAQKIEWHESGGPSGGPSGGSERMLKALNDQLNRELYS
AYLYFAMAAYFEDLGLEGFANWMKAQAEEEIGHALRFYNYIYDRNGRVELDEIPKPPKEWESPLKAFEA
AYEHEKFISKSIYELAALAEEEKDYSTRAFLEWFINEQVEEEASVKKILDKLKFAKDSPQILFMLDKELSAR
APKLPG-

Figure S2: Optimization of molecular tethers on the maxi-ferritin scaffold for SAv mediated conjugation of pMHC molecules. **(A)** 19 (L1-L19) unique linkers that varied in length and rigidity were tested as molecular tethers at the N-terminus of each maxi-ferritin subunit. The linker L6 (highlighted in green) was chosen for further characterization based on protein yield, homogenity and SAv loading onto the functionalized scaffold. **(B)** Sequence of optimized maxi-ferritin scaffold for multivalent pMHC display. Sequence features: CD5 signal peptide (blue),

biotinylation signal sequence (purple), linker (green), residues 3-167 of the ferritin homolog from *Pyrococcus furiosus* (black). The stop codon is indicated by a hyphen (red).

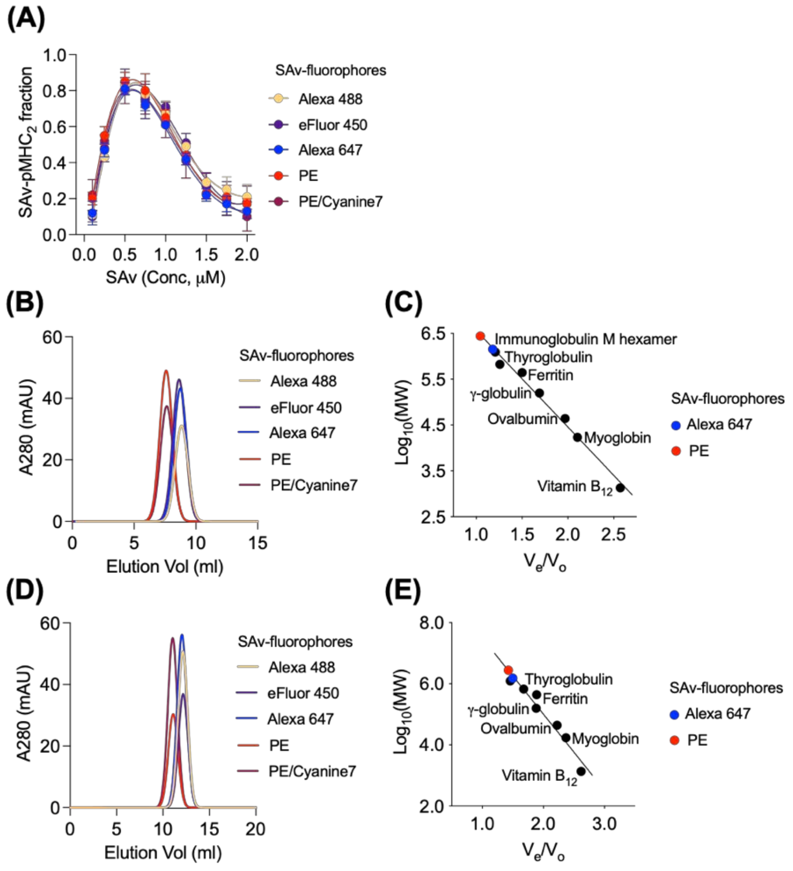

Figure S3: Spheromer assembly is not perturbed by the inclusion of differently sized fluorophores. (A) Comparison of SAv-pMHC2 dimer formation for different fluorophore labelled SAv. **(B)** The size-exclusion profile of spheromers assembled with multiple SAv-fluorophore conjugates. **(C)** Molecular weight estimation of different spheromer complexes (as indicated) using the calibration curve generated using a wide range of standard molecular weight markers. **(D)** The elution profile of spheromer complexes assembled with different SAv-fluorophore conjugates analyzed on a Superose 6 Increase 10/300 GL size-exclusion column. **(E)** The size estimation of different spheromer complexes (as indicated). The Superose 6 Increase 10/300 GL column was calibrated using a wide range of standard molecular weight markers.

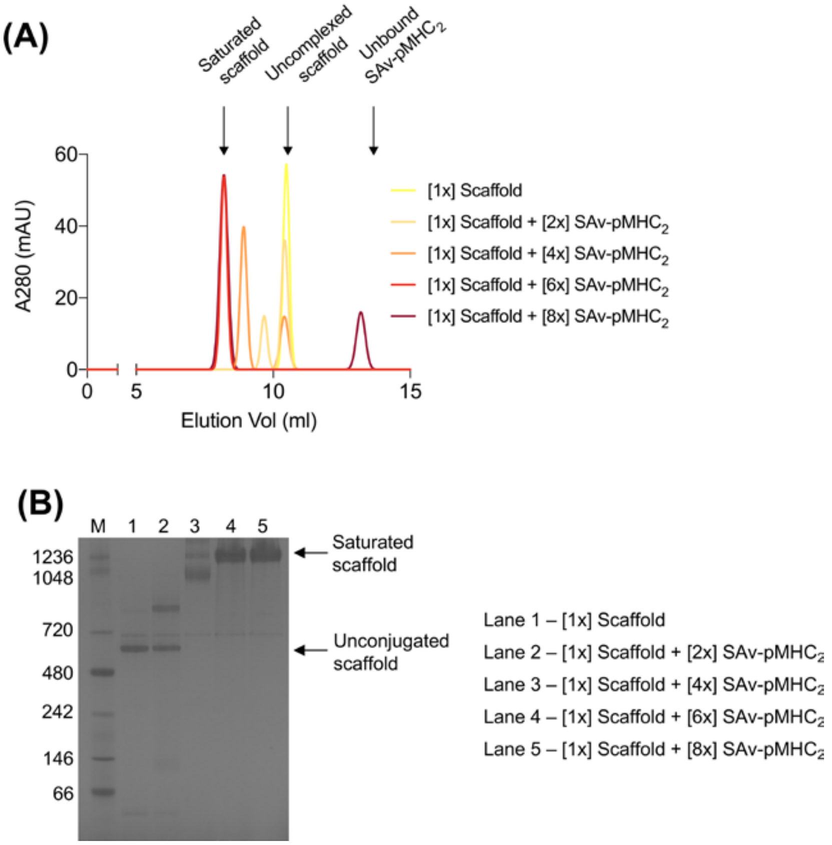

Figure S4: Titration of semi-saturated SAv-pMHC$_2$ with the functionalized scaffold. (A) Size-exclusion chromatography was used to determine the number of SAv-pMHC$_2$ molecules conjugated to the maxi-ferritin scaffold upon saturation. No free reactants were detectable when the scaffold was incubated with SAv-pMHC$_2$ at a molar ratio of 1:6. The scaffold is not saturated at lower (<6) molar ratios of SAv-pMHC$_2$. Also, at a higher molar ratio of SAv-pMHC$_2$ (>6), no further shift in the elution volume of the assembled spheromer was observed. (B) The saturation of the functionalized scaffold was also probed by analyzing the mobility of maxi-ferritin incubated with increasing molar ratios of SAv-pMHC$_2$ using Blue native PAGE (BN-PAGE). The size of the assembled complex does not change after the addition of 6 SA-pMHC$_2$ molecules per maxi-ferritin scaffold, indicating that 12 pMHC molecules are displayed at saturation.

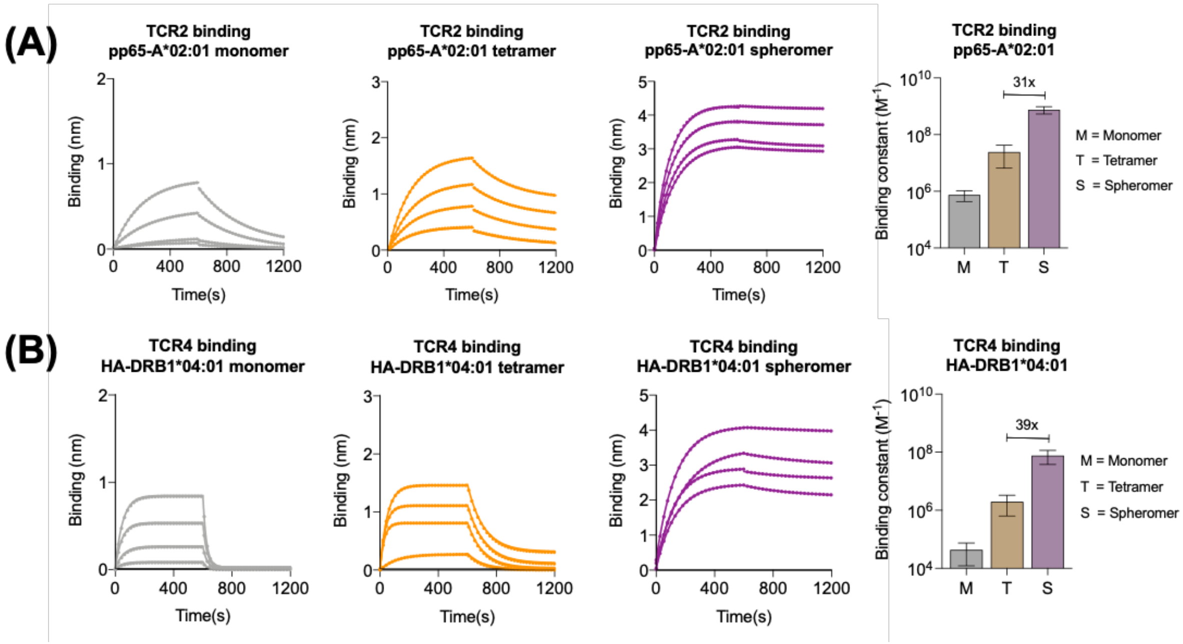

Figure S5: pMHC-TCR binding affinity measurements by biolayer interferometry. The binding of **(A)** TCR2 and **(B)** TCR4 to different formulations of pp65-A*02:01 and HA-DRB1*04:01 respectively are shown. An overlay of binding traces from one representative experimet is shown. Each binding experiment was repeated at least thrice. The mean±SD of the binding constant has been plotted.

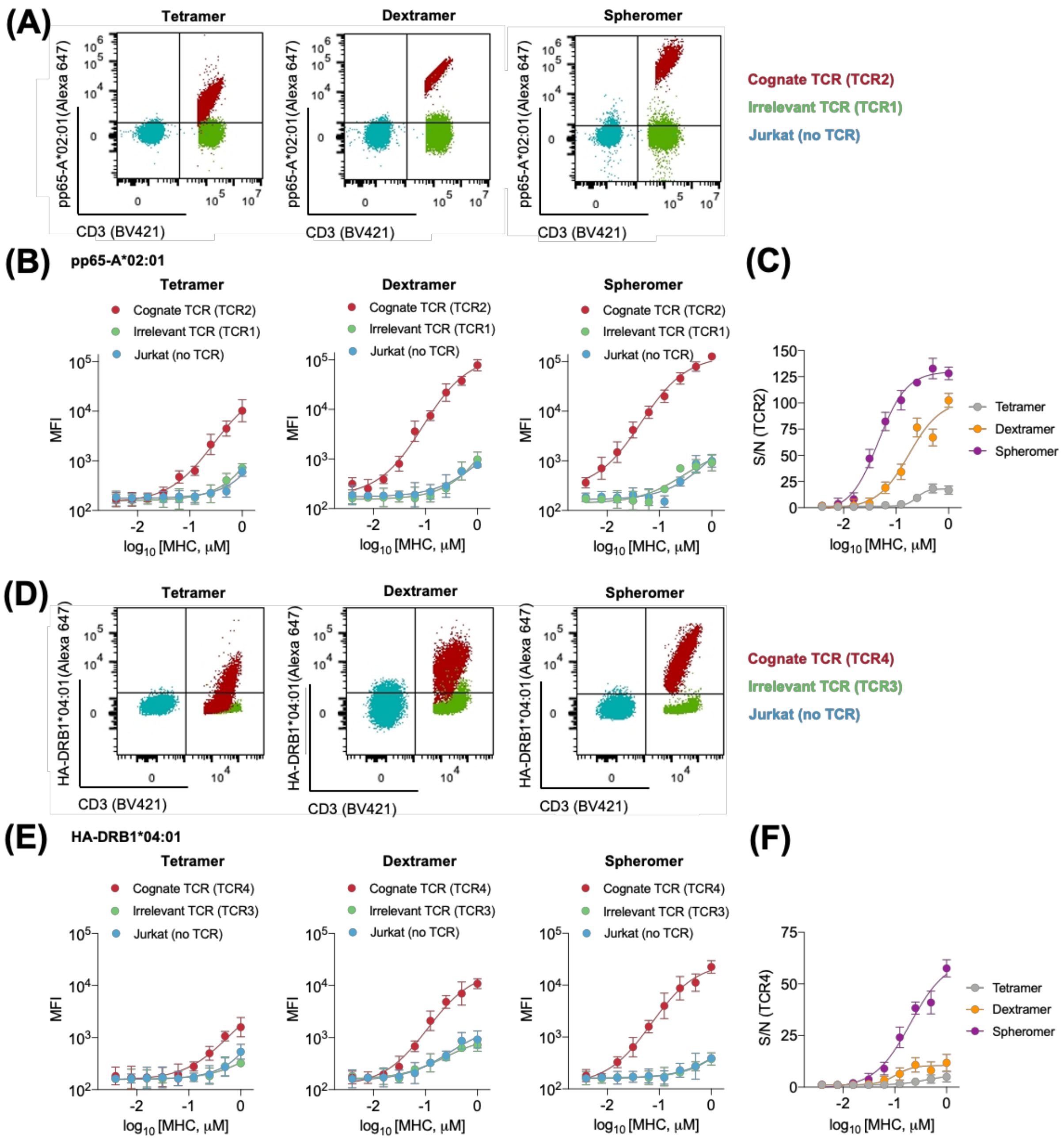

Figure S6: Staining of T cell lines with pMHC multimers. (A) Representative flow cytometry plots showing the binding of the indicated pp65-A*02:01 multimers at an equivalent pMHC concentration to a T cell line expressing TCR2. The non-specific binding to untransduced Jurkat cells and a cell line expressing an irrelevant TCR was also measured. CD3 expression was measured as a proxy for TCR. **(B)** Quantification of pp65-A*02:01 binding measured by flow

cytometry (mean±SD). The experiment was performed with each sample processed in duplicates and repeated at least twice. **(C)** The signal to noise ratio (S/N) of TCR2 binding to distinct pp65-A*02:01 multivalent formulations. Mean±SD of the measurements from two independent experiments has been plotted. **(D)** Representative flow cytometry plots showing the binding of the indicated HA-DRB1*04:01 multimers formulated with equivalent pMHC concentration to cells expressing the cognate TCR (TCR4). The non-specific binding to Jurkat cells and an irrelevant TCR was also measured. CD3 was used as a proxy for TCR expression. **(E)** Quantification of HA-DRB1*04:01 binding (mean±SD) measured by flow cytometry. **(F)** The signal to noise ratio (S/N) of TCR4 binding to distinct HA-DRB1*04:01 multivalent formulations (mean±SD).

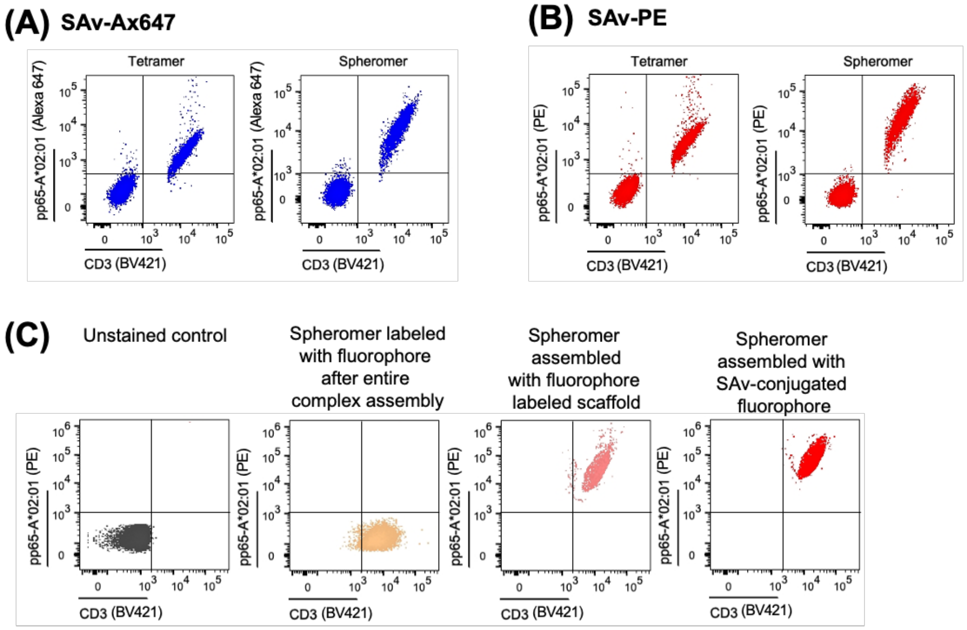

Figure S7: Spheromers stain better than tetramer irrespective of the conjugated fluorophore. Representative flow cytometry plots showing the staining of TCR2 cell line (described in Fig. 2) mixed with TCR$^-$ Jurkat cells with the cognate pp65-A*02:01 tetramer or spheromer labeled with **(A)** Alexa 647 or **(B)** PE. **(C)** Representative flow cytometry plots comparing the staining of TCR2 cell line (described in Fig. 2) with different preparations (as indicated) of the pp65-A*02:01 spheromer.

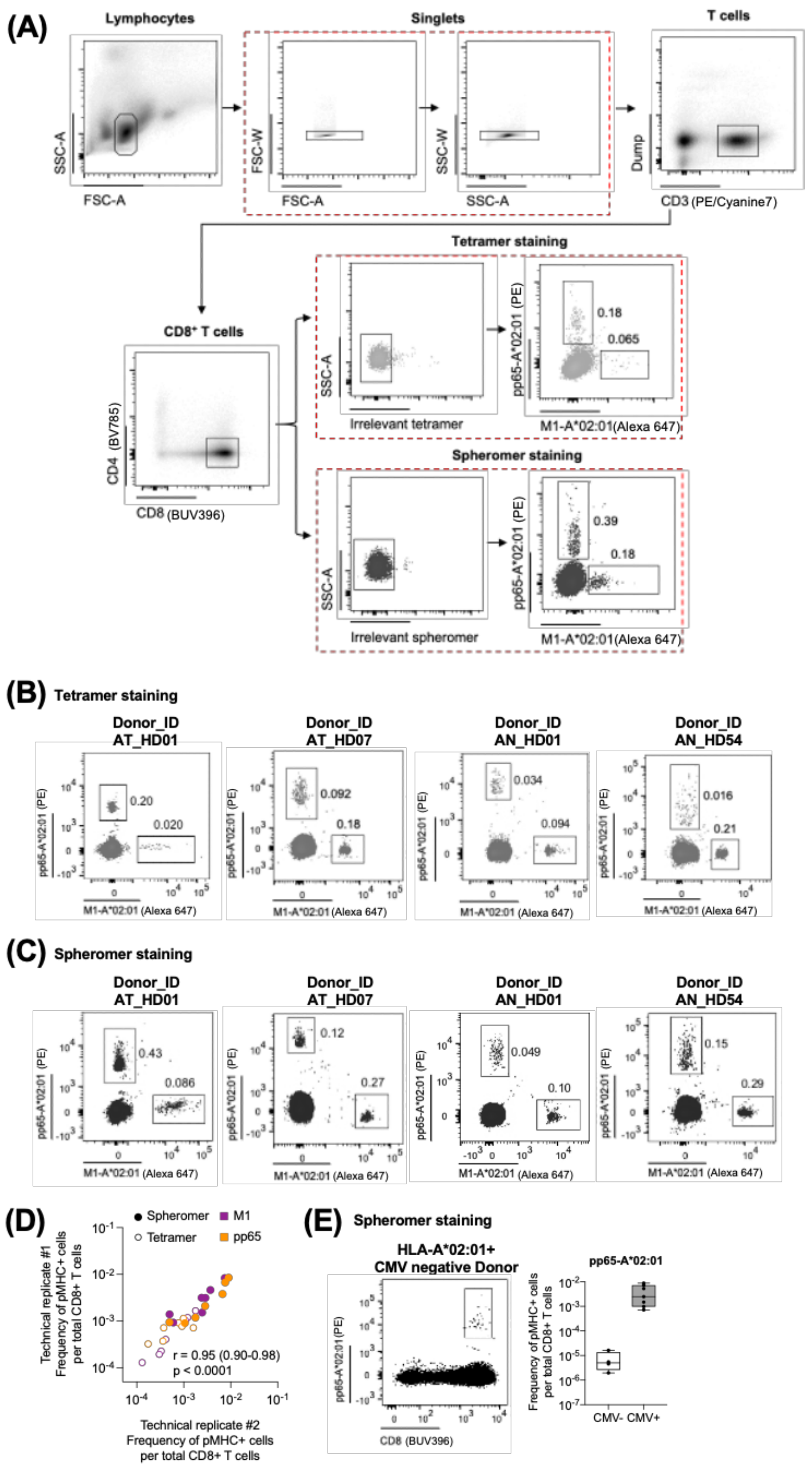

Figure S8: Gating strategy and representative flow cytometry dot plots comparing tetramer and spheromer staining on the same sample. (A) CD8^{+} T cells enriched from PBMCs by

negative selection were equally distributed and stained with tetramers or spheromers (M1-A*02:01 and pp65-A*02:01). Cells were also stained with the gag-A*02:01 pMHC-multimer as an ‘irrelevant’ specificity control. Flow cytometry dot plots comparing **(B)** Tetramer and **(C)** Spheromer (M1-A*02:01 and pp65-A*02:01) staining of PBMC samples across different donors. The PBMCs from each donor were split equally for either tetramer or spheromer staining. **(D)** Correlation between the frequency of antigen specific $CD8^+$ T cells estimated using the tetramer or spheromer on the same donor samples in two independent experiments. **(E)** Representative flow cytometry plot showing the staining of $CD8^+$ T cells isolated from a HCMV- donor with pp65-A*02:01 spheromer after magnetic enrichment. The precursor frequencies determined in naïve, unexposed individuals (n=4) is much lower than the frequencies observed for the HCMV-pp65 epitope in HCMV+ donors.

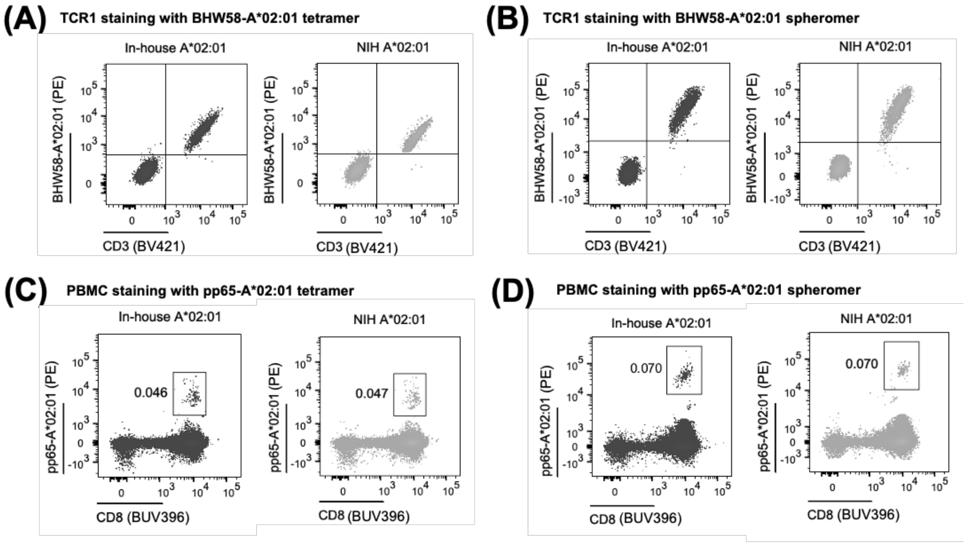

Figure S9: Comparison of pMHC multimer staining using reagents generated in-house or procured from the NIH tetramer core facility. The staining of TCR1 cell line (described in Fig. 2) mixed with TCR^- Jurkat cells with BHW58-A*02:01 **(A)** Tetramer and **(B)** Spheromer. PBMCs from an HLA-A*02:01+ donor was divided equally and stained with pp65-A*02:01 **(C)** Tetramer and **(D)** Spheromer assembled using in-house or NIH reagents.

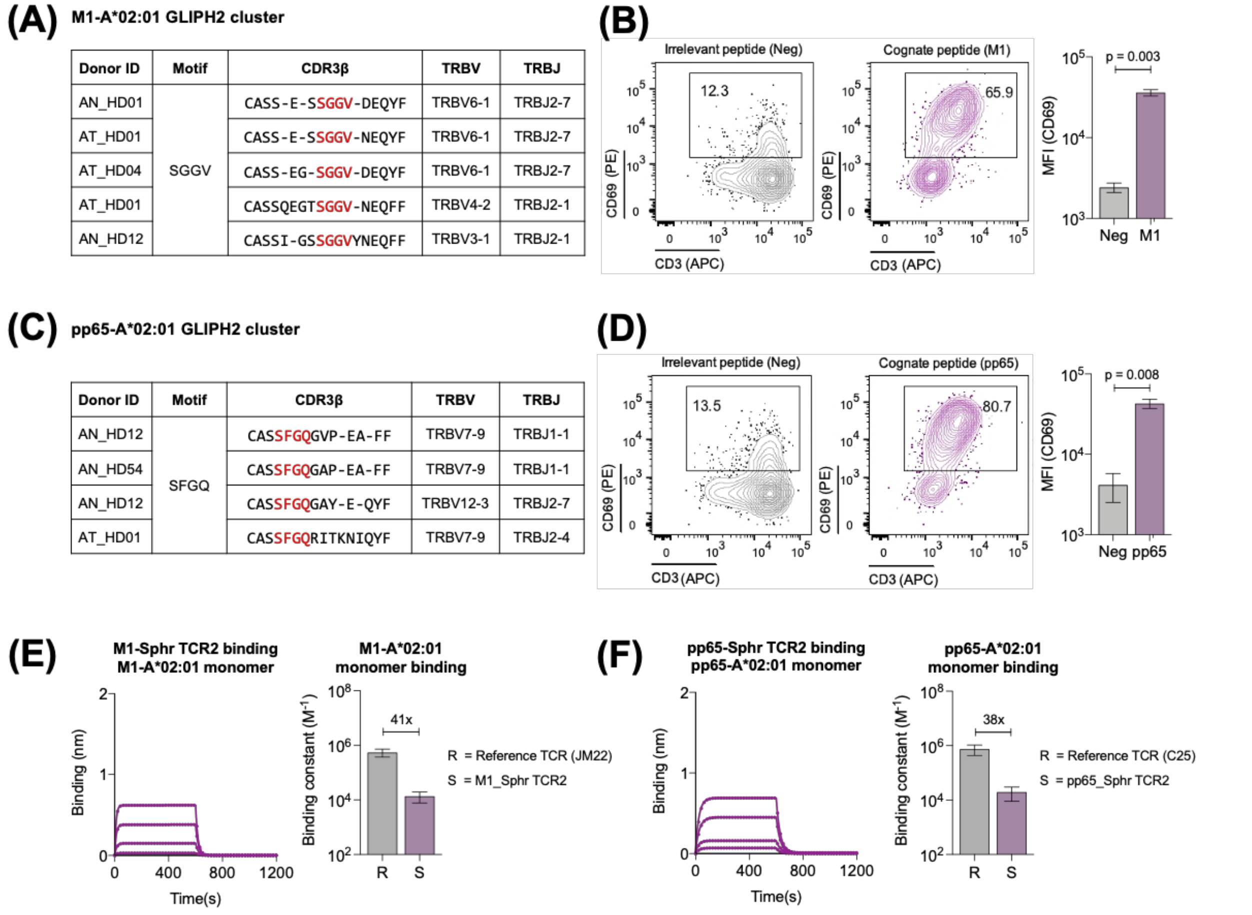

Figure S10: Validation of the unique antigen-specific TCR motifs identified using spheromer. (A) Representative GLIPH2 cluster with influenza-M1 specificity that is composed exclusively of spheromer derived TCR sequences. **(B)** Representative flow cytometry plots showing the activation of a T cell line (expressing a TCR with the "SGGV" motif) stimulated with an irrelevant or cognate (influenza-M1) peptide. The activation was measured by CD69 expression. The significance level was determined by a two-tailed, paired t-test. **(C)** GLIPH2 cluster with HCMV-pp65 specifcity. The cluster was composed of TCR sequences identified exclusively using the spheromer. **(D)** Representative flow cytometry plots showing the activation of a T cell line (expressing a TCR with "SFGQ" motif) stimulated with an irrelevant or cognate (HCMV-pp65) peptide. The activation was measured by CD69 expression. The significance level

was determined by a two-tailed, paired t-test. The binding of TCR corresponding to clones from GLIPH2 clusters comprised exclusively of spheromer derived sequences to their cognate pMHC monomers **(E)** M1-A*02:01 and **(F)** pp65-A*02:01 determined by biolayer interferometry. Each binding experiment was repeated at least three times. The mean±SD of the binding constant has been graphed and compared to a reference influenza-M1 (JM22) and HCMV-pp65 (C25) specific TCR.

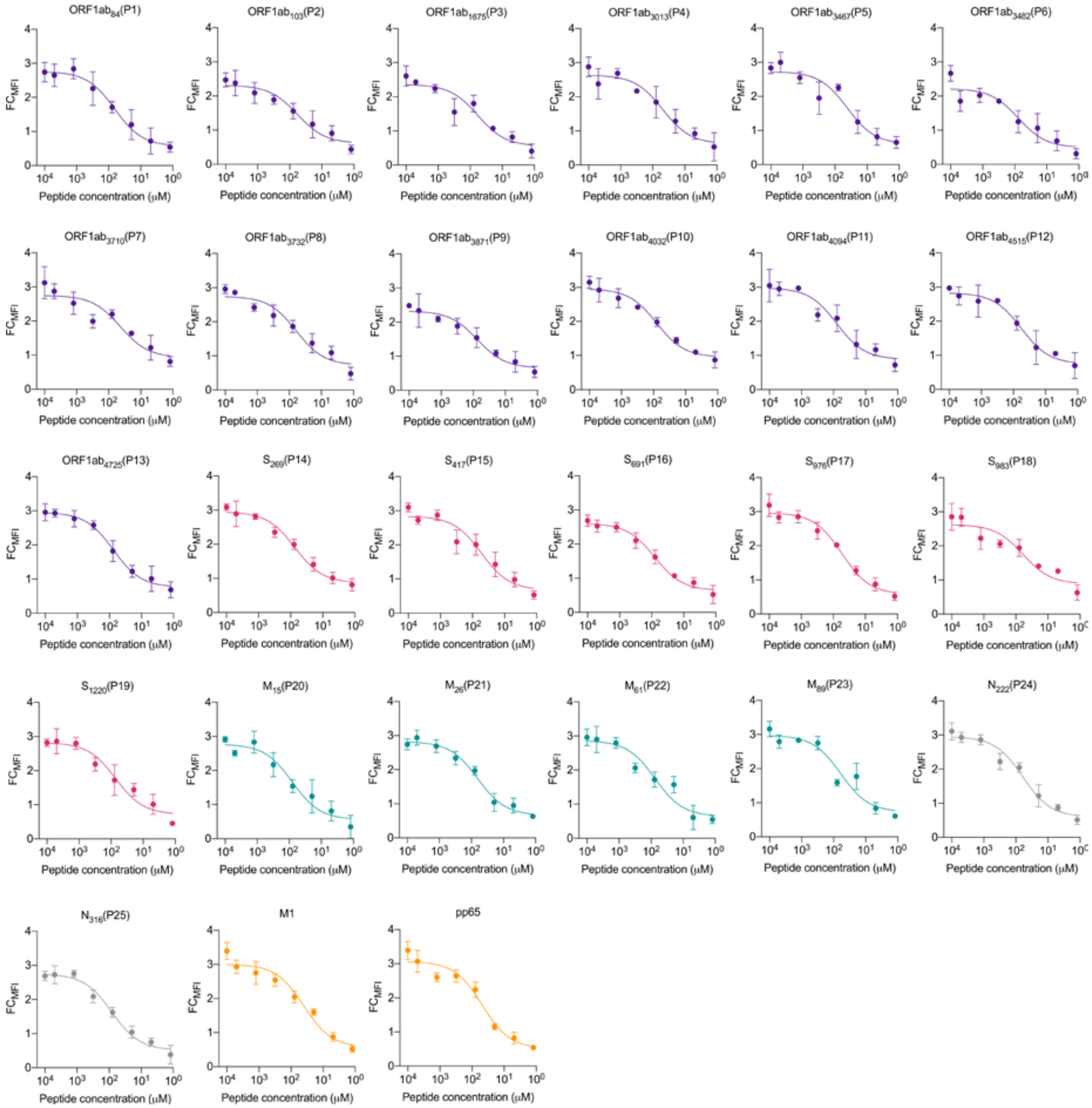

Figure S11: Experimental validation of the predicted SARS-CoV-2 peptide binding to HLA-A*02:01. The binding of test peptides was monitored by an MHC stabilization assay using the TAP-deficient T2 cell line expressing HLA-A*02:01. A productive peptide binding event leads to the stabilization of MHC molecules on the surface of T2 cells that was monitored by flow cytometry. Fold-change (mean±SD) in MFI (test peptide/negative control) has been graphed. The experiment was performed with duplicates and repeated twice. The well characterized HLA-

A*02:01 binding peptides (influenza-M1 and HCMV-pp65) were included as positive controls. The sequences corresponding to SARS-CoV-2 peptides P1-P25 are listed in Table S1.

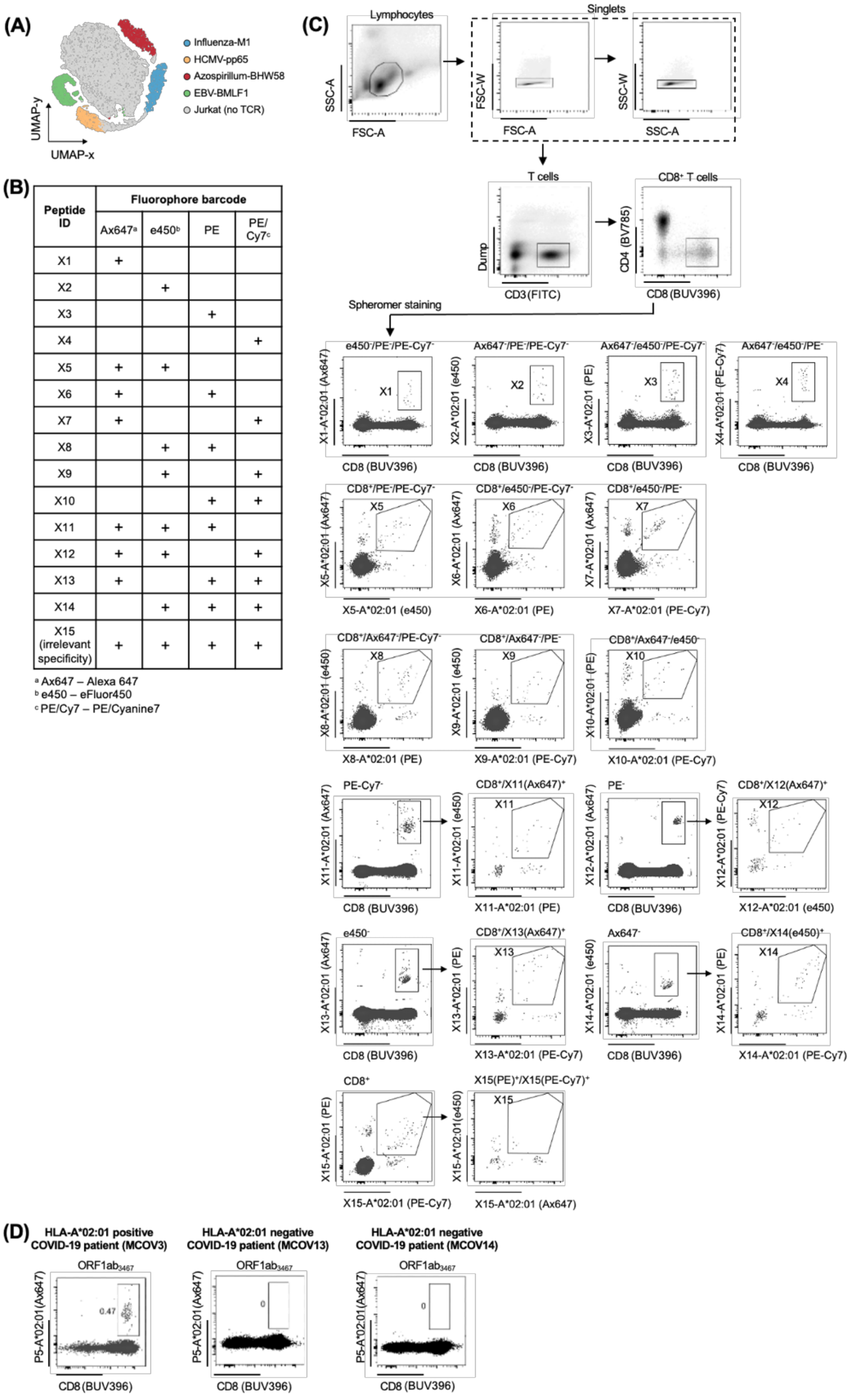

Peptide ID	Fluorophore barcode			
	Ax647[a]	e450[b]	PE	PE/Cy7[c]
X1	+			
X2		+		
X3			+	
X4				+
X5	+	+		
X6	+		+	
X7	+			+
X8		+	+	
X9		+		+
X10			+	+
X11	+	+	+	
X12	+	+		+
X13	+		+	+
X14		+	+	+
X15 (irrelevant specificity)	+	+	+	+

[a] Ax647 – Alexa 647
[b] e450 – eFluor450
[c] PE/Cy7 – PE/Cyanine7

Figure S12: Combinatorial staining with spheromer pools to resolve multiple antigen specificities simultaneously was adapted from a previously described approach. (A) The

relative pMHC monomer concentrations for each flourophore label (Alexa 647, eFluor 450, PE and PE/Cyanine7) to detect multiple epitope specificities simultaneously was experimentally determined using a mixture of untransduced Jurkat cells with four T cell lines that had specificities to influenza-M1, HCMV-pp65, Azospirillum-BHW58 and EBV-BMLF1. UMAP embedding of the cell mixture stained with optimized pMHC concentrations for each fluorophore tag shows the resolution of distinct antigen specificties with almost no overlap. **(B)** Table illustrating the assignment of unique flourophore barcodes for the simultaneous detection of fifteen unique antigen specificties (P1-P15), wherein the P15 combination is assigned to an irrelevant gag-A*02:01 specifictity. **(C)** The gating strategy and representative flow cytometry dot plots to characterize antigen-specific $CD8^+$ T cells using combinatorial spheromer staining. Representative plots showing the deconvolution of antigen specificties based on the unique fluorophore barcodes after staining $CD8^+$ T cells with a pool of spheromers followed by magnetic bead enrichment. **(D)** Staining of T cells with the spheromer is highly specific. Representative flow cytometry plots comparing the staining for SARS-CoV-2 $ORF1ab_{3467}$-A*02:01 specific $CD8^+$ T cells across HLA-A*02:01 positive and HLA-A*02:01 negative COVID-19 patients with mild symptoms. No antigen-specific $CD8^+$ T cells could be detected in HLA-A*02:01 negative COVID-19 patients even post magnetic enrichment.

Table S1: Summary statistics of the study cohorts. Demographic and clinical information of the study participants with age, sex, and CMV infection status.

		Pre-pandemic samples (SARS-CoV-2 naïve)	COVID-19 patients	
			**Mild**	**Severe**
**Number of individuals**		11	15	11
**Age (mean±SD, years)**		50.7±13.9	43.7±18.8	50.3±16.1
**Sex**	**Male (N,%)**	5 (45.5%)	9 (60.0%)	3 (27.3%)
	**Female (N,%)**	6 (54.5%)	6 (40.0%)	8 (72.7%)
**CMV+ (N,%)**		7 (63.6%)	n.d.[1]	n.d.[1]

[1]n.d. = not determined.

Table S2: Demographic and clinical information for COVID-19 patients. All COVID-19+ samples analyzed in this study were collected from adults who tested positive in an RT-PCR assay for the SARS-CoV-2 virus from analysis of nasopharyngeal swab specimens.

Patient ID	Case severity[1]	WHO score	HLA-A*02 expression	Age	Sex	Race/Ethnicity
MCOV1	Mild	n.a.[2]	+	27	Male	Black
MCOV2	Mild	n.a.	+	61	Male	White
MCOV3	Mild	n.a.	+	64	Male	Hispanic/Latino
MCOV4	Mild	3	+	78	Male	Asian
MCOV5	Mild	2	+	26	Male	Hispanic/Latino
MCOV6	Mild	n.a.	+	24	Female	Hispanic/Latino
MCOV7	Mild	2	+	28	Female	Other
MCOV8	Mild	2	+	56	Female	Other
MCOV9	Mild	2	+	22	Female	Asian
MCOV10	Mild	1	+	35	Female	White
MCOV11	Mild	2	+	34	Female	Asian
MCOV12	Mild	n.a.	-	72	Male	White
MCOV13	Mild	n.a.	-	53	Male	White
MCOV14	Mild	n.a.	-	31	Male	Asian
MCOV15	Mild	n.a.	-	45	Male	White
SCOV1	Severe	6	+	77	Male	White
SCOV2	Severe	n.a.	+	37	Male	Asian
SCOV3	Severe	n.a.	+	41	Male	Hispanic/Latino
SCOV4	Severe	5	+	35	Female	Native

SCOV5	Severe	n.a.	+	31	Female	Unknown
SCOV6	Severe	6	+	55	Female	Other
SCOV7	Severe	n.a.	+	44	Female	White
SCOV8	Severe	5	+	60	Female	White
SCOV9	Severe	7	+	68	Female	Black
SCOV10	Severe	n.a.	+	36	Female	Hispanic/Latino
SCOV11	Severe	n.a.	+	69	Female	Hispanic/Latino

[1]The severity of COVID-19 illness at blood-draw was defined based on the symptom score described by Chen et. al. *(63)*.

[2]n.a. = not available. The clinical staff could not assign WHO scores for all participants due to limited data.

Table S3: SARS-CoV-2 peptide panel. Spheromer displaying these peptides in the context of HLA-A*02:01 were used to assess the $CD8^+$ T cell response in unexposed individuals and COVID-19 patients. The sequence similarity (%) with the closest hCoV peptides predicted as strong HLA-A*02:01 binders (percentile rank <5.0) for each seasonal strain is also given.

SARS-CoV-2					Seasonal hCoVs			
**ID**	**Protein**	**Start**	**Sequence**	**Average conservation (%)**	**Sequence**	**Strain**	**Pairwise Similarity[1] (%)**	**Percentile rank[2]**
P1	ORF1ab	84	VMVELVAEL	69.5	GMLLLVGMV	OC43	66.7	1.9
					LMLDKQGQL	NL63	66.7	0.73
					VIVDKVGEL	HKU1	77.8	0.8
					LMCDIVCTI	229E	66.7	1.3
P2	ORF1ab	103	TLGVLVPHV	66.7	ILAVIVVLV	OC43	66.7	1.9
					TLGGLHLLI	NL63	55.6	0.73
					ILAIVVVLV	HKU1	66.7	0.8
					VVAVLLNNV	229E	77.8	1.3
P3	ORF1ab	1675	YLATALLTL	72.3	ILATSDLSV	OC43	66.7	0.09
					SIASTVLTV	NL63	77.8	0.16
					YTWTTILSL	HKU1	66.7	0.33
					QVASGVIDI	229E	77.8	2.6
P4	ORF1ab	3013	SLPGVFCGV	66.7	SVAAITSGV	OC43	66.7	0.31
					SYNGVFLGV	NL63	66.7	4.2
					TINGLVCIV	HKU1	66.7	2.6
					SVFNIFQAV	229E	66.7	0.21
P5	ORF1ab	3467	VLAWLYAAV	97.3	FLAWLYAAI	OC43	88.9	0.42
					VVAFLYAAL	NL63	100	3.1
					VIAWLYAAI	HKU1	100	2.5
					VVAFLYAAI	229E	100	3.3
P6	ORF1ab	3482	FLNRFTTTL	63.9	ALQFTTLTL	OC43	55.6	0.4

					YLNELSSKV	NL63	66.7	0.01
					GLQLFTTWL	HKU1	66.7	0.76
					FLNTVVSTV	229E	66.7	0.03
P7	ORF1ab	3710	TLMNVLTLV	66.7	TVFNVPTKV	OC43	66.7	0.32
					ELAQVLTEV	NL63	66.7	0.19
					SQGNVVTSV	HKU1	66.7	0.67
					SLLSVTSVV	229E	66.7	0.2
P8	ORF1ab	3732	SMWALIISV	63.9	SLNALIATA	OC43	55.6	0.2
					SMFSLLRTS	NL63	66.7	3.2
					SIFGAILAI	HKU1	66.7	0.1
					TMHGVTLKI	229E	66.7	0.56
P9	ORF1ab	3871	VLLSVLQQL	69.4	VLLNCLQHL	OC43	66.7	0.4
					VILTPIFCL	NL63	66.7	1.2
					VLLNCLQHL	HKU1	66.7	0.4
					VVAVLLNNV	229E	77.8	0.86
P10	ORF1ab	4032	MLFTMLRKL	88.9	MLFSMVRKL	OC43	100	0.35
					LLFGMLRRL	NL63	77.8	0.38
					MLFSMVRKL	HKU1	100	0.35
					LLFGMLRRL	229E	77.8	0.38
P11	ORF1ab	4094	ALWEIQQVV	66.7	GVFEVDDTV	OC43	66.7	0.2
					GVSERINVV	NL63	66.7	0.8
					TLFYVNYLV	HKU1	66.7	0.19
					GVSEIVNTV	229E	66.7	0.08
P12	ORF1ab	4515	TMADLVYAL	77.8	TMLDLCYAL	OC43	77.8	0.29
					TMMDLVYAM	NL63	77.8	0.03
					TMLDLCYAL	HKU1	77.8	0.29
					TMMDLCFAL	229E	77.8	0.17
P13	ORF1ab	4725	IFVDGVPFV	94.4	IFVDGVPFV	OC43	100	0.28
					VFIDGVPLV	NL63	88.9	0.3

					IFVDGVPFV	HKU1	100	0.28
					VFIDGVPVV	229E	88.9	0.49
P14	S	269	YLQPRTFLL	61.1	PLTPRQYLL	OC43	66.7	3.5
					YNVTRTFYV	NL63	55.6	4.1
					FVTNKTYNI	HKU1	66.7	0.65
					TLQELSYKL	229E	55.6	0.06
P15	S	417	KIADYNYKL	58.3	VIGDLNCTL	OC43	55.6	1.3
					RLAALNAFV	NL63	55.6	0.08
					KLSDFESEL	HKU1	55.6	0.04
					KSAELNYTV	229E	66.7	0.62
P16	S	691	SIIAYTMSL	61.1	TLAATSASL	OC43	66.7	0.36
					SVVNATVTV	NL63	66.7	0.28
					SALAKIQSV	HKU1	55.6	1.1
					FLLTNTSSV	229E	55.6	0.03
P17	S	976	VLNDILSRL	77.8	SLQEILSRL	OC43	88.9	0.04
					TLVDGVSRL	NL63	66.7	0.04
					SLQEILSRL	HKU1	88.9	0.04
					VLIFVVSML	229E	66.7	0.74
P18	S	983	RLDKVEAEV	66.7	RLDALEAEA	OC43	77.8	0.63
					RLAALNAFV	NL63	55.6	0.08
					RLDALEAQV	HKU1	77.8	0.08
					WLNRVETYI	229E	55.6	0.46
P19	S	1220	FIAGLIAIV	63.9	FINGIFAKV	OC43	66.7	0.05
					RLAALNAFV	NL63	66.7	0.08
					RLDALEAQV	HKU1	55.6	0.08
					FVGALPKTV	229E	66.7	0.69
P20	M	15	KLLEQWNLV	55.6	IILTTFNCV	OC43	55.6	2.2
					LILTVFIVV	NL63	55.6	2.3
					PVIEDYHTL	HKU1	55.6	2.9

					VILTIFIVI	229E	55.6	2.9
P21	M	26	FLFLTWICL	55.6	HLYIQGIKL	OC43	55.6	0.31
					WVFFGFSIL	NL63	55.6	4
					HLYIQGVKL	HKU1	55.6	0.36
					VLWLLWPLV	229E	55.6	0.8
P22	M	61	TLACFVLAA	52.8	SLGIILLFI	OC43	55.6	0.92
					QLPKYVIVA	NL63	55.6	0.86
					SLGVILLFI	HKU1	55.6	0.83
					TLLSGVLYV	229E	44.5	0.01
P23	M	89	GLMWLSYFI	63.9	IIMWIVYFV	OC43	77.8	0.41
					TLTLLSGVL	NL63	44.5	4.5
					IVIWILYFV	HKU1	66.7	0.89
					LVMWVMYFA	229E	66.7	2.3
P24	N	222	LLLDRLNQL	52.8	VLSENLNAY	OC43	55.6	3.6
					AIIEIVNEV	NL63	66.7	0.01
					ELFDSLNLS	HKU1	44.5	2.9
					VVLTFTTRV	229E	44.5	0.38
P25	N	316	GMSRIGMEV	38.9	GTSDPQFPI	OC43	33.4	1.4
					AIIEIVNEV	NL63	44.5	0.01
					MADEIANLV	HKU1	33.4	0.56
					IMKAVAAAL	229E	44.5	0.99

[1]Pairwise sequence similarity was calculated using the sequence manipulation suite(*53*) allowing for amino acid substitutions (GA, VLI, FYW, ST, KR, DE and NQ) with similar biochemical properties.

[2]Percentile rank was calculated using the immune epitope database and analysis resource (IEDB) recommendations (http://tools.iedb.org/mhci/)(*36*).
