# Supplementary material for: CD8+ T cells specific for conserved coronavirus epitopes correlate with milder disease in patients with COVID-19
Source: Sci Immunol. 2021 Jul 1;6(61):eabg5669. doi: 10.1126/sciimmunol.abg5669 (PMC8975171; doi:10.1126/sciimmunol.abg5669)
Supplement: Supplementary file 4 — MDAR Checklist [file sciimmunol.abg5669_mdar_checklist.zip › sciimmunol.abg5669_MDAR_Checklist.docx]

Materials Design Analysis Reporting (MDAR)

Checklist for Authors

The MDAR framework establishes a minimum set of requirements in transparent reporting applicable to studies in the life sciences (see Statement of Task: doi:10.31222/osf.io/9sm4x.). The MDAR checklist is a tool for authors, editors and others seeking to adopt the MDAR framework for transparent reporting in manuscripts and other outputs. Please refer to the MDAR Elaboration Document for additional context for the MDAR framework.

Materials

| **Antibodies** | **Yes (indicate where provided: page no/section/legend)** | **n/a** |
| --- | --- | --- |
| For commercial reagents, provide supplier name, catalogue number and RRID, if available. | The antibody clone and supplier name for all reagents used in this study are given in the methods section.  Pg. No. 25/Lentiviral transduction for generating T cell lines  Pg. Nos. 27-30/ PBMC staining and flow cytometry  Pg. No. 34/ In vitro stimulation of T cell lines |  |
|  |  |  |
| **Cell materials** | **Yes (indicate where provided: page no/section/legend)** | **n/a** |
| **Cell lines:** Provide species information, strain. Provide accession number in repository **OR** supplier name, catalog number, clone number, **OR** RRID | Details for all the cell lines used in this study are provided in the methods section.  Expi293F (Thermo Fisher Scientific)  E.coli (New England BioLabs)  Lenti-X 293T cells (Takara Bio)  TCR-deficient Jurkat cells (α^-^β^-^) (ATCC)  Sf9 insect cells (Expression Systems)  High Five cells (Thermo Fisher Scientific) |  |
| **Primary cultures:** Provide species, strain, sex of origin, genetic modification status. |  | n/a |
|  |  |  |
| **Experimental animals** | **Yes (indicate where provided: page no/section/legend)** | **n/a** |
| **Laboratory animals:** Provide species, strain, sex, age, genetic modification status. Provide accession number in repository **OR** supplier name, catalog number, clone number, **OR** RRID |  | n/a |
| **Animal observed in or captured from the field:** Provide species, sex and age where possible |  | n/a |
| **Model organisms:** Provide Accession number in repository (where relevant) **OR** RRID |  | n/a |
|  |  |  |
| **Plants and microbes** | **Yes (indicate where provided: page no/section/legend)** | **n/a** |
| **Plants:** provide species and strain, unique accession number if available, and source (including location for collected wild specimens) |  | n/a |
| **Microbes:** provide species and strain, unique accession number if available, and source |  | n/a |
|  |  |  |
| **Human research participants** | **Yes (indicate where provided: page no/section/legend)** | **n/a** |
| Identify authority granting ethics approval (IRB or equivalent committee(s), provide reference number for approval. | All human biological samples were collected according to protocols approved by Stanford’s IRB.  Pg. No. 26/ Human Biological Sample Collection |  |
| Provide statement confirming informed consent obtained from study participants. | All participants consented prior to enrolling in the study. |  |
| Report on age and sex for all study participants. | Summary level statistics provided for all study cohorts in Supplementary Table S1.  Demographic and clinical information for COVID-19 patients are provided in Supplementary Table S2. |  |

Design

| **Study protocol** | **Yes (indicate where provided: page no/section/legend)** | **n/a** |
| --- | --- | --- |
| For clinical trials, provide the trial registration number **OR** cite DOI in manuscript. |  | n/a |
|  |  |  |
| **Laboratory protocol** | **Yes (indicate where provided: page no/section/legend)** | **n/a** |
| Provide DOI or other citation details if detailed step-by-step protocols are available. | All experimental protocols are described in detail in the methods section with relevant references as applicable. Detailed step-by-step protocol are available for the following methods and have been cited accordingly.  Pg. No. 32/Single-cell paired αβ-TCR sequencing/ A. Han, J. Glanville, L. Hansmann, M. M. Davis, Linking T-cell receptor sequence to functional phenotype at the single-cell level. Nat Biotechnol 32, 684-692 (2014).  Pg. No. 33/ Identification of TCR ‘motifs’ with shared antigen specificity using GLIPH2/ H. Huang, C. Wang, F. Rubelt, T. J. Scriba, M. M. Davis, Analyzing the Mycobacterium tuberculosis immune response by T-cell receptor clustering with GLIPH2 and genome-wide antigen screening. Nat Biotechnol 38, 1194-1202 (2020). |  |
|  |  |  |
| **Experimental study design (statistics details)** | **Yes (indicate where provided: page no/section/legend)** | **n/a** |
| State whether and how the following have been done**, or** if they were not carried out. |  |  |
| Sample size determination | No statistical tests were performed to predetermine the sample size of the unexposed, healthy individual cohort. Sample sizes for COVID-19 patient groups were determined based on sample availability. |  |
| Randomisation | SARS-CoV-2 infection was confirmed by PCR test results of the nasopharyngeal swab specimens and allocated to the different groups (mild or severe COVID-19) based on their clinical status. |  |
| Blinding | No blinding was done for this study. |  |
| Inclusion/exclusion criteria | Participants were excluded if they were taking any experimental medications (i.e., those medications not approved by a regulatory agency for use in COVID-19.) |  |
|  |  |  |
| Sample definition and in-laboratory replication | **Yes (indicate where provided: page no/section/legend)** | **n/a** |
| State number of times the experiment was replicated in laboratory | All results were reliably reproduced, and replicate information are provided in each associated figure legend. |  |
| Define whether data describe technical or biological replicates | The details for biological or technical replicates are also provided in the methods section and figure legends. |  |
|  |  |  |
| Ethics | **Yes (indicate where provided: page no/section/legend)** | **n/a** |
| Studies involving human participants: State details of authority granting ethics approval (IRB or equivalent committee(s), provide reference number for approval. | Studies were performed in compliance with the protocols approved by the Institutional Review Board of Stanford University. Written informed consent was obtained from all study participants.  Pg. No. 26/ Human Biological Sample Collection |  |
| Studies involving experimental animals: State details of authority granting ethics approval (IRB or equivalent committee(s), provide reference number for approval. |  | n/a |
| Studies involving specimen and field samples: State if relevant permits obtained, provide details of authority approving study; if none were required, explain why. |  | n/a |
|  |  |  |
| Dual Use Research of Concern (DURC) | **Yes (indicate where provided: page no/section/legend)** | **n/a** |
| If study is subject to dual use research of concern, state the authority granting approval and reference number for the regulatory approval |  | n/a |

Analysis

| **Attrition** | **Yes (indicate where provided: page no/section/legend)** | **n/a** |
| --- | --- | --- |
| State if sample or data point from the analysis is excluded, and whether the criteria for exclusion were determined and specified in advance. | No data was excluded from analysis. |  |
|  |  |  |
| **Statistics** | **Yes (indicate where provided: page no/section/legend)** | **n/a** |
| Describe statistical tests used and justify choice of tests. | All statistical tests and the software used for analysis are provided in the methods sections and the corresponding figure legends.  Pg. No. 34/ Statistical analysis |  |
|  |  |  |
| **Data Availability** | **Yes (indicate where provided: page no/section/legend)** | **n/a** |
| State whether newly created datasets are available, including protocols for access or restriction on access. | The study describes the generation of an improved αβ T cell staining reagent called “spheromer”. The details for the purification and assembly are provided in the methods section.  Pg. No. 17/ Design, expression and characterization of multimeric protein scaffolds  Pg. No. 19/ Spheromer assembly and characterization  We have also included a statement for sharing the spheromer reagent as follows:  Pg. No. 41/ Data and materials availability  The reagents required for spheromer assembly will be made available from the corresponding author upon completion of a standard material transfer agreement (MTA) in accordance with Stanford technology transfer policy. |  |
| If data are publicly available, provide accession number in repository or DOI or URL. | Pg. No. 17/ Design, expression and characterization of multimeric protein scaffolds (UniProt accession IDs)  Pg. Nos. 30-32/ Selection of SARS-CoV-2 peptides and sequence conservation analysis (GenBank accession ID) |  |
| If publicly available data are reused, provide accession number in repository or DOI or URL, where possible. | Pg. No. 33/ Identification of TCR ‘motifs’ with shared antigen specificity using GLIPH2 (VDJdb database)  D. V. Bagaev et al., VDJdb in 2019: database extension, new analysis infrastructure and a T-cell receptor motif compendium. Nucleic Acids Res 48, D1057-D1062 (2020). |  |
|  |  |  |
| **Code Availability** | **Yes (indicate where provided: page no/section/legend)** | **n/a** |
| For all newly generated code and software essential for replicating the main findings of the study: |  |  |
| State whether the code or software is available. | No new code or software was generated in this study. |  |
| If code is publicly available, provide accession number in repository, or DOI or URL. |  | n/a |

Reporting

| **Adherence to community standards** | **Yes (indicate where provided: page no/section/legend)** | **n/a** |
| --- | --- | --- |
| MDAR framework recommends adoption of discipline-specific guidelines, established and endorsed through community initiatives. Journals have their own policy about requiring specific guidelines and recommendations to complement MDAR. |  |  |
| State if relevant guidelines (eg., ICMJE, MIBBI, ARRIVE) have been followed, and whether a checklist (eg., CONSORT, PRISMA, ARRIVE) is provided with the manuscript. |  | n/a |
